# Asymmetric attrition and secondary chromosome destabilization after double-strand breaks in human embryonic development

**DOI:** 10.1038/s41467-026-73891-7

**Published:** 2026-06-03

**Authors:** Jenna Turocy, Stepan Jerabek, Woonyung Hur, Jimin Kim, Shuangyi Xu, Qiaojin Zhao, Jia Xu, Alex Robles, Xiangyi Liu, Nathan Treff, Diego Marin, Anna-Katerina Hadjantonakis, Dieter Egli

**Affiliations:** 1 Columbia University Fertility Center, Department of Obstetrics and Gynecology, Columbia University, New York, NY USA; 2 Division of Molecular Genetics, Department of Pediatrics and Naomi Berrie Diabetes Center, Columbia Stem Cell Initiative, Columbia University, New York, NY USA; 3 Developmental Biology Program, Sloan Kettering Institute, Memorial Sloan Kettering Cancer Center, New York, NY USA; 4 Masters of Biotechnology Program, Columbia University, New York, NY USA; 5 Genomic Prediction Inc., Brunswick, NJ USA; 6 Department of Human Genetics. Rutgers University, Piscataway, NJ USA; 7 Present Address: Nucleus Genomics, New York, NY USA

**Keywords:** Centromeres, Embryology, Double-strand DNA breaks, Genomic instability

## Abstract

DNA repair in human embryos is poorly understood, and double-strand breaks (DSBs) can cause chromosome loss. We show that chromosomal alterations relative to an induced DSB are asymmetric: acentric arms show complementary gains and losses, while centric arms are biased toward losses. Centromeric to the cut site secondary breakage and attrition is extensive. In contrast, break sites at acentric arms are conserved with no secondary breakage. These differences reflect differential forces at the mitotic spindle. Telomeric arms detach from the pro-metaphase spindle while centric truncated chromosomes lag during anaphase, suggesting that the DSB impedes sister chromatid separation. Secondary breakage near the centromere concordant with extensive attrition at the DSB site indicates a DSB can destabilize a chromosome without end-joining of sister chromatids. These results highlight the risks of chromosomal-scale changes in CRISPR-Cas9 genome editing and show that a single DSB can destabilize a human embryo chromosome independent of fusion-breakage cycles.

## Abstract (web summary)

Here the authors use induced DNA double strand breaks to investigate mechanisms leading to genome instability in human embryos. They find that acentric chromosome fragments fail to segregate, while centric fragments show attrition at the break site, and secondary breakage near the centromere thus explaining the loss of entire chromosomes.

## Introduction

DNA double-strand breaks (DSBs) occur spontaneously and frequently in the human embryo^1^, but the mechanisms of repair and the genetic consequences are challenging to assess, as they occur at variable locations in the genome. CRISPR-Cas9 systems allow the precise introduction of a defined genetic lesion to interrogate the mechanisms of repair and the genetic consequences.

Recent studies in human embryos have shown that a single DSB can result in extensive genetic changes, including frequent chromosome loss in early human development^2,3^. In a prior study using a single guide RNA (gRNA) targeting a mutation in the *EYS* gene on chromosome 6, DNA breaks resulted in partial or whole chromosome loss in approximately half of the embryos^3^. Alanis-Lobato and colleagues have likewise reported chromosome change at the *POU5F1* locus after CRISPR-Cas9 injection^2^. Whether failure to reseal the DSB or abnormal repair results in chromosomal change in human embryos is not currently known. Not all CRISPR-Cas9-mediated human embryo studies, however, have examined karyotypes after CRISPR-Cas9 injection (reviewed in ref. ^4^), and thus it is unknown how broadly applicable these findings are to other sites in the genome. Interhomolog repair has been proposed as a possible interpretation of the loss of heterozygosity (LOH) in humans at the *MYBPC3* locus^5^ and has been demonstrated in mice^6^. Though any path to LOH can be an adverse genetic outcome, distinguishing loss from a copy-neutral LOH is critical, as they convey different insight into the DNA repair mechanisms active in the early embryo. Copy-neutral LOH suggests proficiency in DSB repair by homologous recombination, while chromosome loss suggests a deficiency. The developmental outcomes also differ: chromosomal changes are often lethal, while copy-neutral LOH can result in imprinting disorders. Given the detrimental possible consequences of DSBs, including duplications and deletions, studies to understand DNA repair pathway choice and the frequency of chromosomal events increase our understanding of developmental failure and genetic disease upon spontaneous breakage. For instance, DSBs can result in deletions and duplications and disorders such as 16p11.2 microdeletion or 16p11.2 duplication syndrome, which have been associated with intellectual disability and psychiatric disorders^7^. Such deletions and duplications may arise through abnormal repair of DNA breaks introduced during either meiosis or during cell cycle progression in the early embryo. CRISPR-Cas9 provides an experimental system to study DSB repair outcomes in the early human embryo, which occur physiologically and spontaneously, enriched near pericentromeric regions as well as at fragile sites on chromosomal arms^8^.

Here, we investigate DNA repair outcomes of CRISPR-Cas9 targeting in the pericentromeric region of chromosome 16 and chromosome X, chosen because they show frequent meiotic aneuploidies in human oocytes^9^, providing an in vitro model for spontaneous chromosome loss and a potential target for ploidy correction in the embryo. Chromosomal changes were frequent at all examined locations and highly asymmetric relative to centromere position. Acentric arms showed both gains and losses in sister cells (with a bias toward losses), while centric arms showed primarily losses with rare gains. Both acentric arms and truncated centric fragments localized to micronuclei prone to embryonic loss. DNA attrition at the cut site was likewise asymmetric: acentric arms showed none or only limited attrition (several kb), suggesting end protection, while centromeric to the break, attrition and secondary breakage caused losses of hundreds of kb to megabases without corresponding gains in sister cells. Live-cell imaging revealed that Cas9 cleavage induces anaphase lag and spindle-dissociated chromosome fragments, reflecting the distinct forces acting on centric versus acentric arms. Together, centromere absence on the acentric side and sister chromatid linkage with anaphase lagging on the centric fragment allow a single DSB to destabilize an entire chromosome.

## Results

### Cas9-induced double-strand breaks result in aneuploidies at different chromosomal locations

**Fig. 1:**
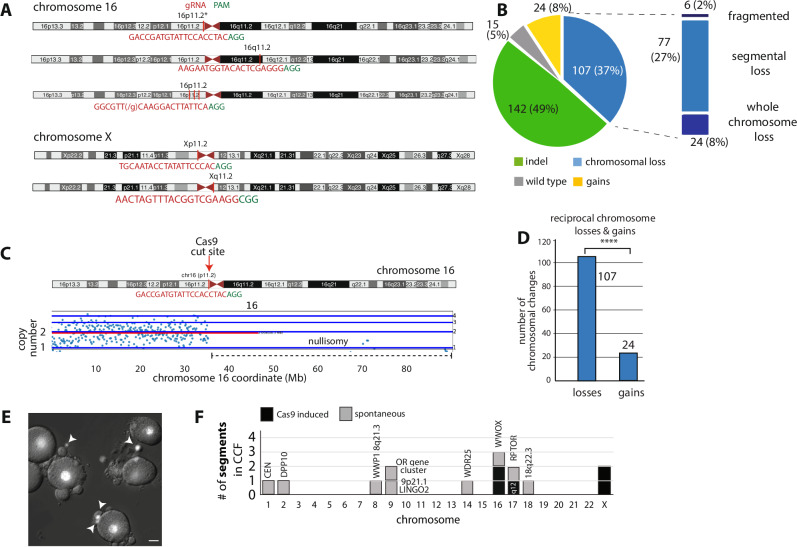
CRISPR-Cas9 double-stand breaks frequently result in chromosome loss. **A** Locations of single guide RNAs (gRNAs) used for CRISPR-Caas9 targeting and generation of DNA double-strand breaks (DSBs) on chromosome 16 and chromosome X. Protospacer sequences with protospacer adjacent motifs (PAM) are shown in red and green, respectively. **B** Quantification of DNA repair outcomes after CRISPR-Cas9-induced pericentromeric DSBs, combining results from both targeted chromosomes X and 16. Each chromosome loss was further categorized as segmental, whole, or fragmented chromosome loss. Segmental losses are defined as those encompassing a chromosomal arm from the Cas9 cleavage site to the telomere. **C** Example of Cas9-induced segmental chromosome loss using SNP array. Shown is a nullisomic loss of the q arm of chromosome 16. **D** Quantification of chromosome gains reciprocal to chromosome losses shown in (**B**), combining results from both targeted chromosomes X and 16 (**** *p* = 0.00001, Fisher’s exact test, two-sided). **E** A representative dissociated Day 3 embryo (one of 7) stained with Hoechst to identify chromosome-containing cellular fragments (CCF). Scale bars, 10 μm. **F** Chromosomal segments excluded from the embryo within cellular fragments (CCF). Combined data from all embryos of the study with spontaneous break sites indicated with coordinates in Supplementary Data 4. For chromosome ideograms, UCSC genome browser was used http://genome.ucsc.edu.

CRISPR-Cas9 gRNAs were designed to target genomic sites in the pericentromeric region of chromosome 16 and chromosome X (Fig. 1A and Supplementary Data 1**)**. One of the gRNAs had three target sites and an off-target site on chromosome 17. We conducted our analysis after injections of Cas9 ribonucleoprotein (RNP) with one to up to three gRNAs into two-pronuclear (2PN) embryos. Embryos were then analyzed at the single-cell level at the cleavage stage and examined for insertions or deletions (indels) to confirm Cas9 activity and for segmental and/or whole chromosome losses. Thirty in vitro fertilized zygotes previously frozen at the 2PN stage were thawed and injected with Cas9 RNP with gRNA into the cytoplasm immediately after thawing. Survival rate post-injection was 96.7% (29/30). Embryos were then cultured to the cleavage stage. One hundred and sixty-nine individual cells from 29 embryos were harvested and individually studied (Supplementary Data 2). Embryos were analyzed at the cleavage stage on day 2 or on day 3. Cellular fragments, defined as having no nucleus in bright field microscopy and being at least a factor of two smaller than nucleated blastomeres in the same embryo, were analyzed as well (Supplementary Data 3), as previous studies in human embryos and in nonhuman primates showed that they could contain excluded chromosomal material^3,10^. DNA from each sample was amplified and analyzed using a high-density single-nucleotide polymorphism (SNP) array, which probes over 800,000 genomic loci to determine copy number and allele heterozygosity along chromosomal arms^11^. Indels and large deletions were evaluated through PCR and Sanger sequencing. Of the 169 samples, ten samples from two embryos revealed LOH along all chromosomes, which is indicative of abnormal fertilization or a lack of genome unification at the first mitosis, and were excluded from further analysis (Supplementary Data 3). Twenty samples showed no detectable genomic DNA, which may be due to being a cellular fragment without genomic material or due to technical causes (Supplementary Data 3). Eight cells, including two with LOH, showed complex chromosomal changes and were reclassified as chromosome-containing cellular fragments. In total, 109 cells and six chromosome-containing cellular fragments from 29 embryos allowed analysis of the consequences of Cas9 activity.

Since blastomeres are diploid, each blastomere contains two homologous chromosome 16 and either one X chromosome in male embryos (XY) or two homologous X chromosomes in female embryos (XX). The gRNAs for chromosome 16 and X were not designed to be allele-specific, thus targeting both homologous chromosomes, and were injected either alone or in combination as indicated in Supplementary Data 2. Such targeting can result in a cell carrying heterozygous or homozygous indels, an indel combined with a chromosomal change, or no chromosomal material at all. The combined number of autosomal and X chromosomal targets in 109 embryo cells is 394 chromosomes, of which 288 molecularly distinguishable Cas9-induced outcomes were detected by PCR and Sanger sequencing (157/288) or chromosomal changes detected in SNP array analysis (131, composed of 107 losses and 24 gains) (Supplementary Data 2).

Indels were found in 49% (142/288) of detectable and distinguishable outcomes, of which 67 were hemizygous or homozygous, representing either two identical indel events or LOH. 5% (15/288) were found to be wild-type (Fig. 1B). Thus, the pericentromeric gRNAs used demonstrated a high on-target efficiency. By far the most frequent indel among all samples in our experiment using human embryos was a −1 base pair (bp) deletion and *a* + 1 bp insertion, which may be the result of processing the stagger created by Cas9 cleavage^12^ (Supplementary Fig. 1A). Gel electrophoresis showed smaller PCR products (Supplementary Fig. 1B), which were confirmed to be the result of CRISPR-Cas9-induced deletions by Sanger sequencing. Indels ranged from −358 to +2 bp (Supplementary Fig. 1A). Using primer pairs of up to 1.3 kilobase (kb) PCR in size, only two indels were larger than 100 bp (Supplementary Fig. 1A). Deletions of 6 bp or larger in human embryos occurred primarily through microhomology-mediated end joining (Supplementary Fig. 1C). These studies confirm the high level of DSB induction and genetic modification at the evaluated sites and point to unrepaired or misrepaired chromosomes as a frequent outcome. Because both chromosomes were targeted, we observed indels with LOH due to a segmental loss on the other chromosome (Supplementary Fig. 1D).

Lack of a PCR product was also common: 60 evaluated sites showed no specific product. The lack of a PCR product can be caused by several events, either technical or biological, including unrepaired chromosomes and aneuploidy. Lack of a PCR product and hemi- or homozygous indels account for the difference between 394 chromosomal targets, and the 288 molecularly identifiable genetic outcomes.

Chromosomal abnormalities were very common: 45% (131/288) among a total of 288 molecularly identifiable Cas9-induced outcomes, demonstrated a gain (8%, 24/288) or a loss (37%, 107/288). Losses were composed of whole chromosome loss 24/288 (8%); segmental chromosome loss 77/288 (27%); and 6/288 (2%) fragmented chromosome loss, resembling chromothripsis (Fig. 1B). Chromosomal changes are apparent as reduced copy number and LOH on SNP-array-based chromosome screening. Reduced copy number and altered allelic ratios flanking the targeted locus are segmental chromosome abnormalities (e.g., Fig. 1C and Supplementary Fig. 2). Figure 1C shows the presence of an acentric fragment and the loss of a centromere-containing chromosome segment. The break points occurred at the Cas9 cut site; thus, segmental losses can readily be attributed to CRISPR-Cas9 activity. (Supplementary Data 2).

Spontaneous versus Cas9-induced whole chromosome losses are molecularly indistinguishable. However, Cas9 may increase overall frequencies of aneuploidy for the targeted chromosome. Chromosome 16 losses were compared among embryos injected with gRNA targeting chromosome 16 versus embryos injected with gRNA targeting chromosome X. However, when it occurs in combination with Cas9-induced segmental errors, it is a plausible outcome of Cas9 activity. Interestingly, one embryo, consisting of four cells (Supplementary Fig. 2A), showed mosaic correction of meiotic trisomy 16 in SNP array analysis. This Supplementary Fig. also explains SNP array analysis of embryo karyotypes: SNP signal intensity can be quantified as chromosomal copy number bar plots for the entire genome, for balanced signal intensity across a single chromosome, for allelic ratio, and for heterozygosity (Supplementary Fig. 2B). Such analysis captures most relevant information regarding Mb-scale chromosomal changes, with on-target analysis being performed by PCR and Sanger sequencing. The combination of polar body analysis and blastomere analysis shows gain of chromosome 16 at the second meiosis (Supplementary Fig. 2C), followed by mosaic Cas9-mediated correction in blastomere 4 (Supplementary Fig. 2D, E). A gRNA targeting 3 sites on chromosome 16p11.2 was used for targeting the trisomy. Targeting a chromosome at three sites simultaneously resulted in efficient chromosome changes (53%: 28 chromosomal changes of 52 target chr16 in 24 cells analyzed, of 8 diploid and 1 trisomy embryos, Supplementary Data 2). Nevertheless, only half of all targeted chromosomes showed altered copy number frequency. Thus, Cas9-induced whole chromosome loss is a consequence of on-target cleavage in human embryos and may potentially be useful for the correction of trisomies, but these will be mosaic.

### Loss of genomic DNA from the embryo

The mechanisms of these chromosomal changes are not well understood. Cas9-induced chromosomal changes in blastomeres were heavily biased towards losses by a factor of ~4.5, suggesting loss of genomic material from the developing embryo. Complementing the 96 losses, there were merely 22 reciprocal segmental gains in another cell of the same embryo (Fig. 1D). We stained seven embryos treated with Cas9 with Hoechst and found that 6/7 (86%) showed excluded cytoplasmic fragments containing DNA (Fig. 1E). Whenever possible, we karyotyped cytoplasmic fragments separately from blastomeres. For six of 27 embryos, we amplified and sequenced chromosomal segments contained in cytoplasmic cellular fragments (Supplementary Data 3). These showed genomic coordinates of breakpoints consistent with Cas9 cleavage on chromosomes 16 or X (Fig. 1F and Supplementary Data 4). Chromosomal segments from one site on chromosome 17 were also seen, which we found to be due to a secondary target site of the gRNA at 17q12 (Supplementary Fig. 3). No other genomic DNA was detected in these cytoplasmic fragments, suggesting that a cleaved arm was entirely disjointed from the rest of the genome and excluded from the embryo.

Chromosomal segments resulting from spontaneous breakages were also observed in cellular fragments. Breakpoints at fragile sites are located at *WWOX*, an olfactory receptor (OR) gene cluster, *WDR25*, *DPP10*, and *RPTOR* (Fig. 1F). These sites are consistent with the patterns of breakage in human embryos^8^: *WWOX* and *DPP10* are known fragile sites, RPTOR is a long gene of an almost 500 kb transcript, and OR gene clusters were shown to be fragile in human embryos. None of these sites were in proximity to a predicted off-target site. These results show that both spontaneous and Cas9-induced chromosome fragments can be excluded from the embryo proper in small cellular fragments.

Overall, among the 27 embryos injected with CRISPR-Cas9 and gRNA targeting the pericentromeric region of a chromosome 16 or chromosome X, 24 (24/27; 89%) demonstrated an unrepaired or misrepaired chromosomal loss in at least one blastomere. These results add to the growing body of literature demonstrating frequent and specific chromosome loss after Cas9-induced DSBs.

### Acentric gains and centric losses—asymmetric outcomes relative to Cas9-induced DSBs

**Fig. 2:**
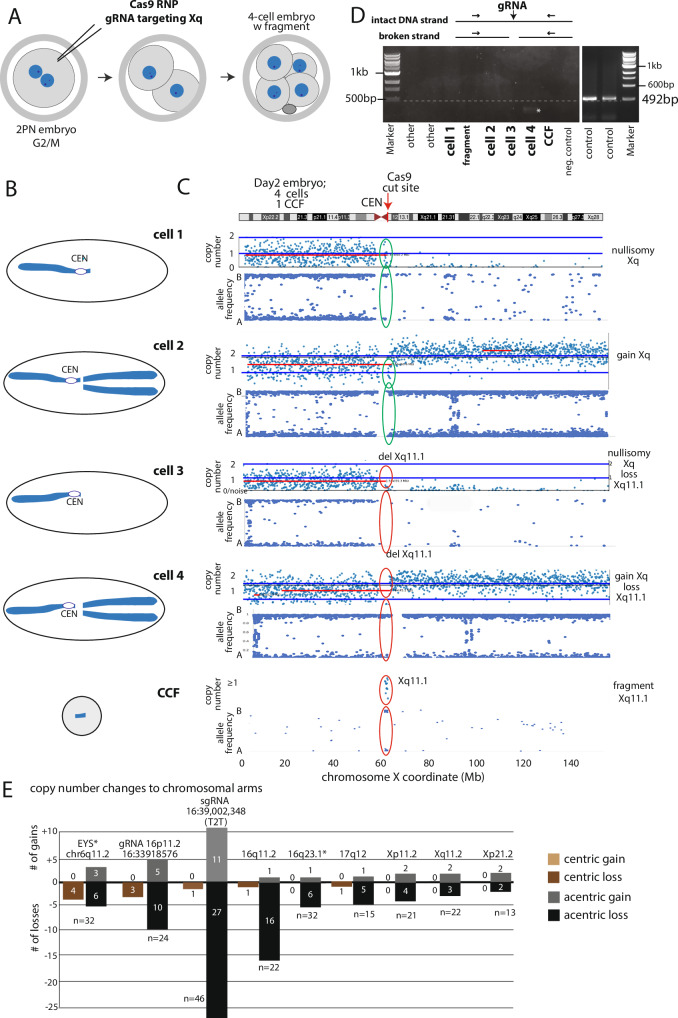
Asymmetric outcomes of Cas9 cleavage relative to the centromere. **A** Schematic of the embryo being analyzed. CRISPR-Cas9 RNP injection was done in G2/M phase. Induction of DNA DSBs due to Cas9 activity expected at the two-cell stage. Products of a single mitosis after Cas9 cleavage are being analyzed. **B** Schematic of chromosomal outcomes in individual blastomeres and one fragment collected from the CRSPR-Cas9-injected human embryo. **C** SNP array data analysis, including copy number plot with allelic ratio for each blastomere. The embryo (D3_16qXq) is male, and the chromosome X is of uniparental origin, resulting in only (**A** and **B**) alleles and no heterozygosity. Note the copy number changes of the acentric q arm, with reciprocal losses and gains. Ovals indicate the presence (green) or absence (red) of genetic material on the targeted chromosome X, and separation of DNA to a CCF shown at the bottom. **D** PCR using primers flanking the Cas9 cut site on chromosome X of the blastomeres of embryo D3_16qXq (treated with gRNAs targeting 16qXq, see Supplementary Data 2 for all on target amplifications *n* = 344). (*) the sequence of this faint band does not map to the human genome and is considered an artifact. Samples with bolded text are from the embryo shown in (**A**–**C**). “Other” refers to cells from different embryos. PCR results for all blastomeres and embryos are shown in Supplementary Data S2. **E** Quantification of segmental chromosome gains and losses relative to the centromere location. Number of gains and losses and total number of blastomeres analyzed are indicated. Data based on Supplementary Data 2, (*) data from ref. ^3^. Source data are provided as a Source Data file.

Most segmental chromosome changes occurred on acentric chromosome arms. Segmental chromosomal gains occurred in reciprocity with segmental or whole chromosome losses: for example, four cells from one male embryo injected with gRNA targeting the pericentromeric chromosome Xq arm were collected on day 2 of development (Fig. 2A). Two of the four blastomeres showed nullisomy for chromosome Xq; the other two blastomeres had a gain of the acentric arm Xq (schematic in Fig. 2B, data in Fig. 2C). Both segmental gains were acentric chromosomal arms. This is consistent with studies in yeast, which show that a single DSB results in co-segregation of sister chromatids consisting of acentric chromosome arms^13^. Both cells with acentric gains showed no amplification of the Cas9 target site using flanking primers (Fig. 2D). Even as these cells contained an apparently full chromosome X complement in the SNP array assay, there was no on-target PCR amplification, indicating that the chromosome was not intact. Such target dropout was common: 60 targets failed to amplify while 288 amplified (Supplementary Data S2).

Chromosomal changes at other sites followed this pattern: at a total of nine sites—including data from a previous study at *EYS* and chromosome 16q23.1^3^ and an additional site on the p arm of chromosome X (Supplementary Data 1)—segmental chromosomal changes were more common for acentric versus centric fragments (Fig. 2E). Though not all sites displayed equal frequency in causing segmental errors, they all showed the same pattern. While acentric segments showed both losses and gains, centric fragments were all infrequent losses. This suggests that segregation of truncated chromosomes containing a centromere does occur, albeit it is error-prone.

The pattern of chromosomal changes can validate genome annotation and the genomic position relative to the centromere. One gRNA site was located on the p arm of chromosome 16 according to the hg38 genome assembly (chr16:35126545 at 16p11.1) and to the q arm according to the T2T genome assembly (chr16:39002348 at 16q11.2). The acentric q arm was primarily affected by Cas9 cleavage at that site, congruent with the location relative to the centromere according to T2T (Supplementary Fig. 4). Other gRNAs on the q arm similarly affected primarily the q arm of chromosome 16 (Supplementary Fig. 5).

At the time of gRNA design, the T2T assembly was not yet available—what appeared to be frequent centric q arm changes did not fit the pattern of the other gRNA targets, which resulted primarily in acentric changes (Fig. 2E). This discrepancy pointed to an error in the genome annotation of hg19 and hg38^14^ and are validating T2T annotation. Others have likewise seen differences in the location of centromeric histones when comparing hg38 and T2T^15^.

### Asymmetric attrition of the chromosome end centromeric but not telomeric of the double-strand break

**Fig. 3:**
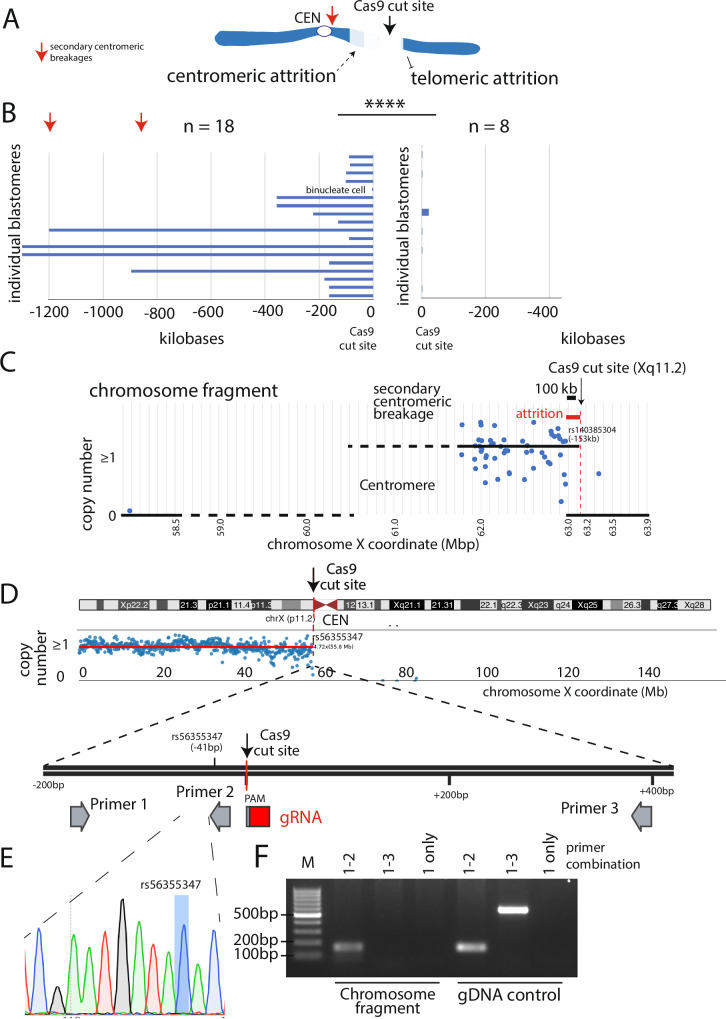
Asymmetric loss of DNA centromeric and telomeric to the DSB. **A** Schematic of a Cas9-induced DNA DSB generating an acentric and a centric fragment relative to the cut site. The faded area (grey) adjacent to the cut site represents attrition of the chromosome. CEN centromere. **B** Chromosomal breaks resulting from Cas9 cleavage at unique target sites on chromosomes 16, 17, X, and 6^3^ were analyzed. The length of each genomic DNA attrition was calculated based on specific gRNA position, and the genomic coordinate of chromosome copy number transition annotated in Supplementary Data 4 (**** *p* = 0.0001, *T* test, two-sided, *n* = 24). **C** Representative example of a chromosome fragment with attrition of genomic DNA centromeric to the Cas9 cut site and a secondary breakage at the centromere of targeted chromosome X, from a total sample number of 24. **D–F** Conservation of the break site telomeric of the Cas9 cut site on the p arm of targeted chromosome X. **D** SNP array signal, location of PCR primers used to assess attrition and potential sister chromatid fusion (one sample from *n* = 8, Supplementary Data 4). **E** Sanger sequence of PCR product using primers 1–2. **F** PCR using primer use (#1) is intended to detect head-to-head end joining of sister chromatids from specific blastomere. Specific assay for this single sample. Genomic DNA is from a control cell line. Source data are provided as a Source Data file.

In addition to segmental chromosome changes, the embryo in Fig. 2C showed a fragment with genetic material between the cut site and the centromere. This genetic material was missing from two sister cells (red circles in Fig. 2C) but was not attritted in the other pair. Loss of genomic material between the Cas9 cut site and the centromere was also seen on chromosome 16 (Supplementary Fig. 4B). To better understand these events and how they might be related to the loss of truncated, centric chromosome fragments, we mapped loss of genomic DNA on either side of the DSB with an accuracy of 41 bp–22 kb using SNP intensity array data (Fig. 3A). We used only nullisomic copy number transitions because of the greater signal difference allowing more accurate mapping, with a resolution determined by the local SNP density on the array (Supplementary Data 4). Loss of genomic DNA was apparent in 17/18 samples centromeric to the Cas9 cut site (Fig. 3B). Loss of genomic DNA centric to the break site occurred in two forms: breakage at the centromere, resulting in the loss of over a Mb, or attrition of several hundred kb adjacent to the cut site. For instance, for one embryo containing a chromosome X fragment, the closest detecData SNP was rs140385304, 153 kb centromeric to the Cas9 cut site (Fig. 3C). In addition, this fragment also had a secondary break at the centromere. The only cell with a centromeric fragment but without detectable attrition towards the centromere was binucleate (sample I1-4, Data S4) due to cytokinesis failure.

In stark contrast, attrition telomeric to the Cas9 cut site was within the resolution of several kb of the SNP array: in 8/8 sites telomeric to the cut site, there was no attrition, or attrition was less than a few kb (Fig. 3B and Supplementary Data 4). We also used PCR primers adjacent to the cut site to evaluate attrition of DNA telomeric to the cut site. In a cell containing an acentric chromosome Xp arm, the closest SNP on the array, rs56355347 at a mere 41 bp telomeric of the Cas9 cut site, showed a detectable signal on the SNP array (Fig. 3D). And a primer pair 20 bp distal to the cut site resulted in specific amplification, verified by Sanger sequencing (Fig. 3E). No PCR product was obtained using primers flanking the cut site, and no PCR product was obtained using primer 1 alone, suggesting the absence of head-to-head end joining of sister chromatids (Fig. 3F). In this male embryo (H1), all eight cells showed reciprocal whole and segmental chromosome changes, demonstrating that the cut site remained conserved through two or three cell divisions, for a total of three days (Supplementary Data 2).

### Micronucleation and lagging chromosomes—consequences of Cas9-induced DSBs

Our karyotyping results thus far suggest that Cas9-induced DSB ends often fail to be rejoined with the appropriate repair partner, resulting in segmental loss through physical separation. Furthermore, our data suggest that sister chromatids are linked at the break site, impeding or preventing segregation of centric truncated chromosomes and resulting in secondary breakages. These events should be apparent through microscopic analysis.

**Fig. 4:**
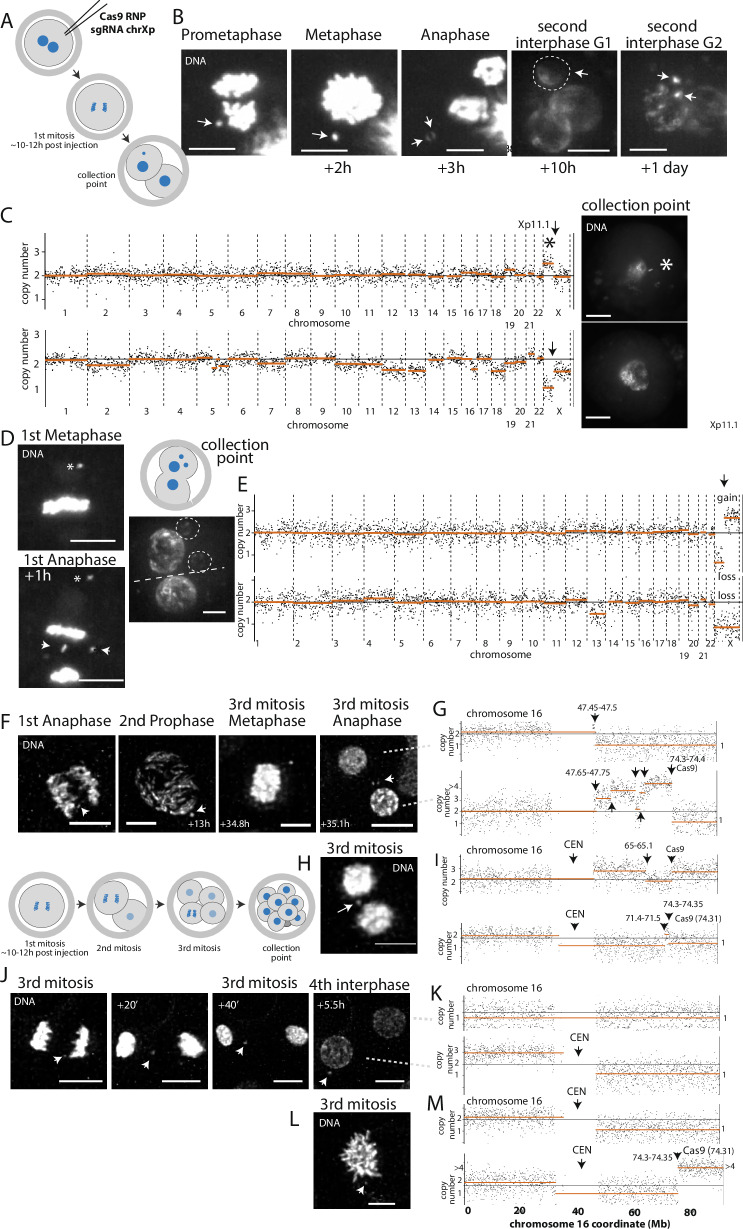
Live-cell imaging of DNA double- strand break-induced aneuploidies. **A** Schematic of the experiment illustrating the Cas9 RNP injection into 2PN human zygote on day 1. Representative images from *n* = 10 imaged embryos are shown. **B** Frames from live-cell imaging of the first mitosis of human embryo after Cas9-induced DNA DSB. Cas9 cleavage occurred >10 h post-injection. Time from the first frame is indicated. Note the dissociated chromosome fragment (arrows in individual frames). Dashed circle indicates micronucleus formed from the chromosome fragment. Related to Supplementary Movie S1. **C** Chromosomal content in individual cells collected at the two-cell stage. (*) indicates the acentric micronucleus fragment visible through microscopy. **D**, **E** Frames from live-cell imaging of the first mitosis (**D**) and corresponding chromosomal content at the two-cell stage (**E**). The cell failed cytokinesis and was dissected in two different parts (indicated by dashed line), each containing one main nucleus. Related to Supplementary Movie 2. **F–M** Frames from live-cell imaging starting at the first mitosis to the eight-cell stage with corresponding chromosome content for each cell. A poorly aligned chromosome was observed at the first mitosis, followed by a micronucleus (**F**). At the third mitosis, two dissociated chromosome fragments were apparent. Corresponding chromosomal content of the cell with the additional micronucleus (**G**). Lagging chromosomes (**H**, **J**) and corresponding chromosome content in two other cell divisions (**I**,** K**). Micronucleus at third mitosis (**L**) and corresponding acentric gain (**M**). Related to Supplementary Movie 3. Scale bars, 10 μm.

To directly observe chromosomal segregation errors in mitosis caused by induced DSBs, we used light sheet live-cell microscopy. The same gRNA targeting Xp as previously used was injected into 2PN embryos (Fig. 4A). We also used a gRNA targeting the arm of chromosome 16q23.1 with off-target sites on chromosomes 10 and 18 (Supplementary Data 1). Targeting the chromosome 16 arm may facilitate visualizing deletions and duplications between gRNA and centromere using microscopy and copy number analysis, with chromosomes 10 and 18 providing additional targets. Using the live cell DNA dye SPY650, we were able to image human embryos from the one-cell stage on day 1 after fertilization to the eight-cell stage on day 3 after Cas9 injection. Ten embryos were successfully imaged, stopped at the indicated stage, and karyotyped, and eight embryos contained Cas9-induced aneuploidies (Supplementary Data 5). In a total of four embryos, spontaneous aneuploidies were absent or limited to one, to allow distinction of Cas9-induced from spontaneous aneuploidies in the imaging data.

In the first cell division, ~10–12 h after Cas9 injection, we observed a chromosome fragment separated from paternal and maternal genomes (Fig. 4B). The fragment failed to assemble on the metaphase plate, segregated separately from the genome in one of the daughter cells, and turned into a micronucleus at interphase (Fig. 4B). By connecting microscopy to karyotypes, it was found to be an acentric chromosome X fragment, a gain in one cell, and a complementary loss in the sister cell (Fig. 4C). Therefore, the micronucleus consisted of two separate sister chromatids. Interestingly, sister chromatids appeared as separate visual signals in mitosis and again towards the end of the second interphase, suggesting they were not a covalently linked and continuous DNA strand (Fig. 4B and Supplementary Movie 1). This is consistent with earlier PCR results; acentric fragments were not covalently joined head-to-head to each other (PCR reaction with primer “1 only” in Fig. 3D).

The second type of abnormality was lagging chromosomes in between two groups of normally segregating chromosomes at anaphase. In one embryo (Fig. 4D), a chromosomal fragment segregating from the spindle at the first metaphase and two lagging chromosomes at anaphase were observed. Cell division was delayed to 24 h post-injection, with asynchronous condensation of paternal and maternal nuclei, cytokinesis failed, and the resulting cell was binucleate (Fig. 4D and Supplementary Movie 2). Mis-segregating chromosomes were packaged into micronuclei during interphase (Fig. 4D). The cell was mechanically split between the two nuclei for genome amplification of arbitrary individual halves (dashed line), revealing a centric gain Xq, complemented with a whole chromosome X loss in the sister cell (Fig. 4E). The loss of an acentric Xp fragment, as well as untargeted chromosomes 13, might have been due to the exclusion from the cell or a consequence of mechanical cell splitting.

Another embryo followed from the one-cell to the eight-cell stage and showed abnormalities on chromosomes 1 and 16 in all cells. Chromosome 1 loss was meiotic in origin, while the remaining cells were chromosomally mosaic with break points on chromosome 16 characteristic of Cas9 cleavage and with secondary attrition between the cut site and the centromere (embryo 3; Fig. 4F–M and Supplementary Data 5). Six blastomeres showed secondary centromeric breakages, and two showed pericentromeric breakage on chromosome 16 (Fig. 4G, I, K, M). This attrition followed anaphase lagging, suggesting difficulties in separation of sister chromatids, either due to sister chromatid end joining or another mechanism (Fig. 4H). Lagging also resulted in whole chromosome changes: a centric gain (Fig. 4K, bottom) was complemented by a whole chromosome loss (Fig. 4J, top nucleus Fig. 4K, top) after mitosis with a lagging chromosome (Fig. 4J). Micronuclei propagating through multiple cell divisions were also observed (Fig. 4F, L). Corresponding karyotypes showed multiple gains (Fig. 4G, M). This is consistent with an acentric fragment in a micronucleus undergoing replication and passage to daughter cells without segregation, resulting in accumulation of multiple copies in a single cell (Fig. 4G, M). As DNA replication in micronuclei is compromised^16^, it can result in fragmentation at mitosis (Fig. 4G). Duplication due to sister chromatid end joining and subsequent breakage of a dicentric chromosome would result in three copies of a duplicated segment, proportional to the linked chromatid. Instead, the data are consistent with secondary pericentromeric breakage and propagation and replication of acentric segments as micronuclei. Such unilateral propagation of micronuclei separate from the main nucleus has previously been described in mice^17^. Remarkably, all blastomeres progressed with normal kinetics through the cell cycle, with ~13 h for the second and ~22 h for the third cell cycle (Fig. 4F and Supplementary Movie 3), indicating that a single or even multiple broken chromatids do not impede cell cycle progression at this stage.

Spontaneous chromosome losses were also observed. In one 8-cell embryo, two blastomeres showed a loss of chromosome 18, while others were euploid (Supplementary Data 5). The error originated at the first mitosis, with the separation of genomic material from the main chromosome mass at anaphase, resulting in a micronucleus (Supplementary Fig. 6A). At the second mitosis, two chromatids were observed, and in the third interphase, a micronucleus was apparent in one of the daughter cells. At the third mitosis, two chromosomal entities were again apparent, dissipating from the main nucleus and apparently being lost from the embryo (Supplementary Fig. 6A). Though the abnormality was most evident at the third mitosis, it originated in the first. Nevertheless, only two of eight blastomeres showed a chromosome loss (Supplementary Fig. 6B, C). The unstable genetic material in micronuclei is lost, contributing to the bias of spontaneous mitotic abnormalities towards chromosome losses^8^.

Complex chromosome segregation errors following multinucleation after the first cell division were also observed (Supplementary Fig. 6D–F and Supplementary Movie 5). These complex abnormalities arose due to three spontaneous segmental errors and two Cas9-induced segmental errors, resulting in a blob of lagging chromatin at anaphase (arrow in Supplementary Fig. 6D). Thus, lagging chromosomes as well as dissociated acentric fragments are a consequence of chromosome breakage in the human embryo.

### Double-strand break ends separate; sister chromatids remain in proximity

**Fig. 5:**
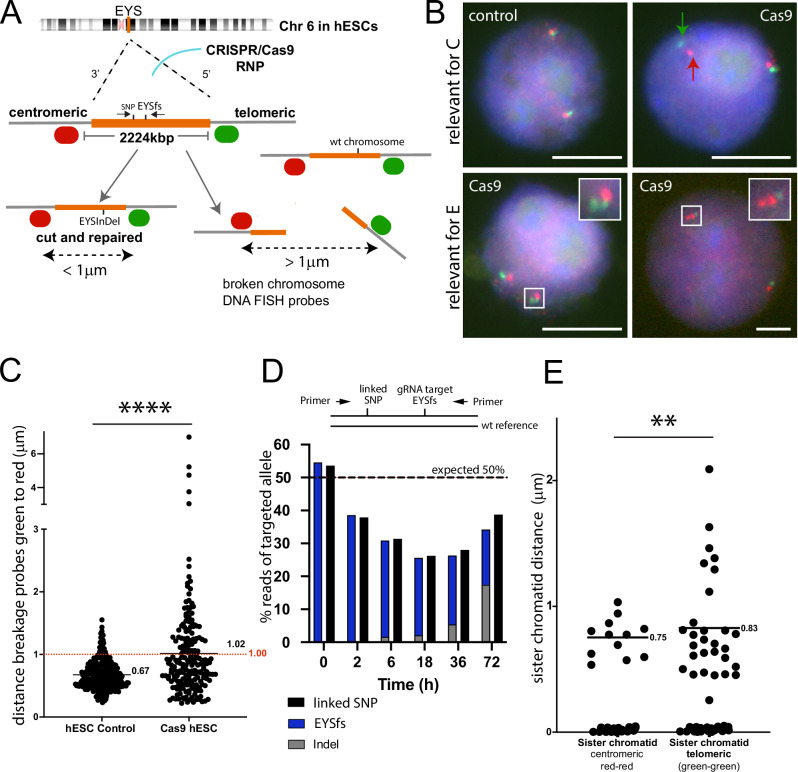
Separation of cleaved chromosome ends after Cas9-induced DNA double-strand breaks in human embryonic stem cells. **A** Schematic depicting separation of DNA break-apart fluorescence in situ hybridization (FISH) probes dependent on the Cas9 cleavage at the *EYS* target site. On an intact human chromosome 6, flanking red and green fluorescent probes are held in proximity to each other. A Cas9-induced DNA DSB at the target locus facilitates physical separation of cleaved chromosome ends. Arrows indicate flanking primers used to amplify the targeted region. **B** Stained individual interphase cells depicting different outcomes following allele-specific CRISPR-Cas9 targeting and induction of DSBs 18 h post-transfection. Scale bars, 10 μm. **C** Quantification of distances between telomeric and centromeric FISH probes flanking the targeted *EYS* locus in untreated controls and Cas9-treated, heterozygous *EYS* cells at 18 h post-transfection. The red dotted line indicates 1.00 μm separation. Mean distances for conditions are listed to the right. Total number of treated cells assessed *n* = 85 pairs. **D** Allele frequency determined after Sanger sequencing analysis of PCR products amplified using primers flanking the Cas9 cut site indicated in (**A**). **E** Analysis of distances (in μm) between duplet signals of FISH probes (green-green) and centric FISH probes (red-red) at Cas9-targeted *EYS* locus on duplicated sister chromatid pairs. Data points represent both single foci (0 μm) distances and non-zero distances. 12/85 cells contained non-zero distances for centric probes (red-red signals). 29/85 cells contained non-zero distances for telomeric probes (green-green signals). Total number of cells assessed *n* = 85.

To directly observe physical separation of broken chromatid ends, we used human embryonic stem cells (hESCs). The use of hESCs allowed us to analyze a larger population of DSB sites while also providing greater experimental accessibility. We used DNA break-apart FISH probes designed to flank the Cas9 site at the *EYS* locus to study nuclear localization following a targeted DSB by measuring the distance between fluorescent signals (Fig. 5A). In untreated control cells, the two signals were in proximity (Fig. 5B). Heterozygous *EYS*^fs/+^ mutant ESCs were used to induce an allele-specific cut on the mutant chromosomes at rs758109813, while the wild-type chromosome remained intact^3^. Three different configurations were observed and quantified, marked by the following criteria: (1) proximity, (2) distant breakage probes indicative of a broken *EYS* locus, and (3) replicated sister chromatids with two distinct dots centromeric or telomeric of the cut site. Due to the heterozygous nature of *EYS*^fs/+^ hESCs and the allele-specificity of the gRNA^3^, one chromosome would remain as intact (as uncut control) while the other is cut. Among 85 cells containing two pairs of green-red foci, the mean distance was markedly increased relative to controls (0.67 μm in controls vs. 1.02 μm in Cas9) (Fig. 5C). In 12 cells, separation was greater than 2 μm and up to 7 μm. Thus, for at least 12/85 cut alleles (14%), DNA DSBs were not re-sealed at the time point analyzed (18 h post-transfection).

To evaluate kinetics of cleavage and repair, we used PCR combined with next-generation sequencing to quantify the targeted *EYS*^fs^ allele relative to the wild-type intact allele. A heterozygous SNP, rs66502009, 99 bp linked to *EYS*^fs^ and centromeric of the Cas9 cut site, was used as an additional readout (Fig. 5D). Across 0–72 h, intact *EYS*^fs^ reads declined relative to the *EYS*^wt^ allele from 54 to 17%, while indel-containing repair products accumulated from 0 to 17.5% (Fig. 5D). At 18 h, intact *EYS*^fs^ and indel alleles together accounted for only 26% of the expected 50% signal, indicating that nearly half of all chromosomes originating from the *EYS*^fs^ allele were no longer amplifiable by PCR. This missing fraction reflects targeted alleles rendered undetectable due to unrepaired DSBs, large deletions, or chromosomal changes. By 72 h, reads from the targeted chromosome partially recovered through accumulation of indel alleles, setting a lower bound on unresolved DSB of 17% at 18 and 36 h. The allelic frequency of the linked SNPs paralleled depression and recovery of a combined *EYS*^fs^ and indel carrying reads (Fig. 5D). Together, these orthogonal assays of FISH and qPCR demonstrate that Cas9 editing at *EYS* generates a substantial fraction of persistent or catastrophic DSB outcomes involving physical separation.

We also measured the distance between sister chromatids when sister chromatids were apparent as two identically colored signals (Fig. 5B). We observed significantly more duplex sister signals telomeric of *EYS* (29/85) than centromeric of *EYS* (12/85), *p* < 0.01, consistent with greater potential for separation of chromosome arms than centromeres prior to mitosis. However, no pair was separated beyond a 2 μm distance (Fig. 5E). This contrasts significantly with the separation seen in cleaved red-green pairs (*p* = 0.0003, Fisher’s exact test, two-sided). Thus, sister chromatids remain in proximity, which allows these ends to become mutual repair partners, potentially resulting in head-to-head fusion of sister chromatids and breakage-fusion-bridge cycles.

### Spontaneous breakages destabilize a chromosome in human blastocysts

**Fig. 6:**
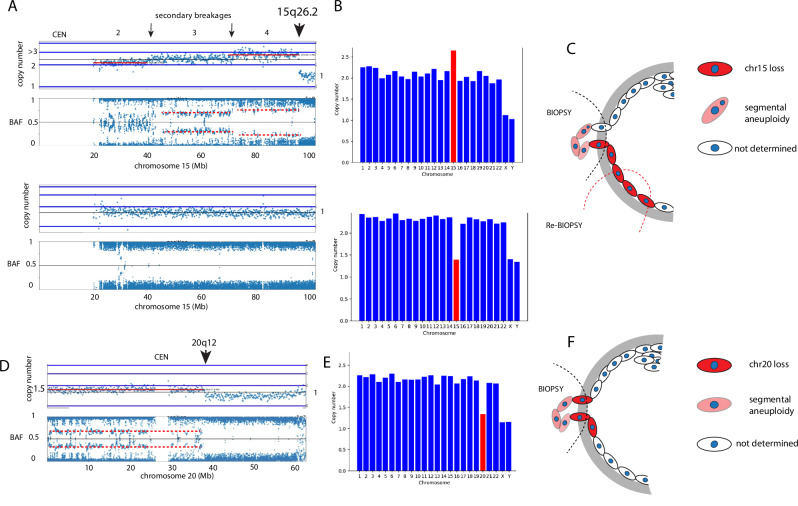
Spontaneous chromosome breakages with attrition centromeric to DNA break site and whole chromosome loss. **A** SNP array data for biopsies from a human blastocyst. Upper panel: A copy number plot for the first biopsy showing a stepwise increase in genetic content of chromosome 15 proximal-to-distal relative to the centromere (CEN), toward the indicated fragile site at 15q26.2. This stepwise increase is reflected with a shift of allelic frequencies, from 0.5 for heterozygous loci to ~2/3 and ~1/3, and ~¼ and ~¾. Lower panel: A copy number plot for the second biopsy of the same embryo shows whole chromosome 15 loss, and a complete loss of heterozygosity throughout the chromosome. **B** The overall copy number for all chromosomes analyzed in two embryo biopsies from the same human blastocyst. Note the gain of chromosome 15 in one biopsy (upper panel), and the loss in the other (lower panel). **C** Model for the human embryo biopsied at the blastocyst stage. Cell color represents chromosome 15 loss (red), segmental abnormality and accumulation due to asymmetric segregation of chromosome fragments as a micronucleus (pink), and unknown genomic content in cells that were not analyzed (white). **D–F** Analysis of a blastocyst biopsy of an embryo mosaic for segmental and whole chromosome 20 loss. A copy number plot showing intermediate copy number for the q and p arms until 20q12, with allelic ratios skewed from the 0.5 due to mosaicism (**D**), and a copy number plot for all chromosomes (**E**). **F** A model of the analyzed embryo, with an indicated biopsy consisting of cells with either segmental or whole chromosome 20 loss.

Human embryos show frequent spontaneous breakages at fragile sites. To understand the consequence of these breakages on the stability of the affected chromosome, we evaluated SNP array data from 711 clinical biopsies obtained from blastocysts (day 5–day 7). These biopsies submitted for preimplantation genetic testing typically consist of 5–10 cells and can thus constitute a group of cells with mosaic chromosomal constitution. Five blastocysts showed segmental loss and a concurrent LOH. The truncated chromosome segment was present as a mosaic, resulting in an intermediate copy number and a shift of the allelic ratio. In one embryo, two different biopsies revealed mosaicism for a segmental and a whole chromosome 15 loss (Fig. 6A–C). A primary break site at chr15:96278873–96283059 was found to be gene poor, characteristic of spontaneous fragile sites (Supplementary Data 6). Secondary breakages between the fragile site and the centromere resulted in distinct portions of accumulated chromosome segments and shifted allelic ratios. A re-biopsy revealed that other cells of the same embryo were monosomic for chromosome 15, suggesting that the primary break at the fragile site had destabilized the chromosome, resulting in whole chromosome loss (Fig. 6B, C). This embryo resembles the Cas9-induced events in Fig. 4F–M. Another embryo with a break site at 20q12 (chr20:38267563-38501640)—another gene-poor region characteristic of embryo fragile sites (Supplementary Data 6)—likewise showed an intermediate copy number and a shift in allelic ratio (Fig. 6D–F). Therefore, spontaneous breakages at fragile sites destabilize the affected chromosome, resulting in both segmental and whole chromosome loss similar to Cas9-induced breakages.

## Discussion

Studying CRISPR-Cas9-induced DNA DSBs in human embryos is relevant for several reasons: understanding the repair of a DSB, which can occur spontaneously; it helps explain the cause of frequent aneuploidies in human embryos. It reveals how DNA breakage can give rise to copy number variants, which can have lasting phenotypic consequences ^18,19^.

Here we find that Cas9-induced pericentromeric DNA DSBs on chromosomes 16 and X result in chromosomal loss in over one-third of chromosomes targeted. Whole chromosome aneuploidy or deletion of chromosome arms are typically incompatible with life. A mitotically acquired aneuploidy compatible with development to term is the mosaic loss of the X-chromosome associated with Turner syndrome^20^. Thus, targeting X-chromosomal genes to correct a mutation using Cas9 could readily result in Turner syndrome. Without even addressing considerations related to ethics and regulation, the genotoxicity alone makes CRISPR-Cas9 an unsuitable tool for heritable editing. Genotoxicity of Cas9 is also well documented in other cell types, ranging from large deletions to segmental chromosome truncations^12,21–26^.

In a basic research context, the genotoxic effects of Cas9-induced DSBs are highly relevant. Because genetic change occurring in the beginning of human life is highly consequential, gene editors provide an extraordinary tool to interrogate the consequences of a defined DNA lesion and thereby gain a better understanding of the repair and the genetic consequences of spontaneous lesions as well. Most such studies are conducted in somatic cells or cancer cells, but these insights do not necessarily translate to the human embryo. The genetic outcomes can be cell-type specific because of cell-type-specific preferences in DNA repair pathway choice. While human embryos are not a traditional experimental context, they have already provided insight into basic cell biology, such as the chromosomal consequence of a single DSB^2,3^, and replication stress in the mammalian embryo^8^.

This study reveals mechanisms of chromosome loss after Cas9 cleavage in human embryos. First, we find that a Cas9-induced DSB has no detecable impact on the kinetics of cell cycle progression in the first three cell divisions (see Supplementary Movies 3–5). The absence of a delay points to weak DNA damage checkpoint control, allowing the cell to enter mitosis with one or even several broken chromosomes. Failure to repair prior to mitotic entry is supported by the observation of dissociated chromosome fragments in live cell microscopy and the corresponding loss of chromosome segments from the embryo in karyotype assays. Because the DNA damage response functions to both delay the cell cycle and promote repair, inefficient repair is the most parsimonious interpretation of the findings presented here. Chromosomal aneuploidies after a DSB also occur in other systems, including in mouse embryos^27^ and in somatic cells^26^. Though these referenced datasets are not directly comparable, using different gRNAs and delivery systems, the frequency of aneuploidy in human embryos described here is much higher. In somatic cells, DSBs inhibit or delay cell cycle progression, but when ATR checkpoint kinase is inhibited, chromosomal aneuploidies also become common^28^. Though ATR kinase is active in the early human embryo^8^, the degree of activation from a single or even several DSBs is apparently insufficient to delay and prevent mitotic entry. Interestingly, the slow kinetics and/or inefficiency of DSB repair is not just applicable to the human embryo but is also seen in stem cell-derived neurons^29^. Also, in human pluripotent stem cells, the closest cultured cell proximate to a human embryo, repair lags DSB break induction by many hours (this study and ref. ^29^). The slow kinetics of repair and poor G2 checkpoint control allow for frequent chromosomal errors.

DSBs result in both whole chromosome and segmental chromosome losses. Loss of acentric chromosome segments is the most common abnormality, as there is no mechanism to attach acentric sister chromatids to a spindle. Acentric segments physically separate from the main chromosome mass during mitosis, and the disjointed segments form micronuclei and are lost from the embryo in subsequent cell divisions. Centric fragments are also prone to undergo segregation errors, albeit less frequently. Lagging chromosomes caused by Cas9 cleavage as observed through live cell microscopy point to difficulties in segregating sister chromatids to daughter cells. The nature of this linkage may be end-joining of sister chromatids, resulting in dicentric chromosomes. The physical proximity of sister chromatids as seen in FISH lend sister chromatid ends to illicit repair, resulting in head-to-head fusion, dicentric chromosomes, and breakage-fusion-bridge cycles. Alternatively, it may simply be reversed forks or unreplicated DNA at the DSB end. These unresolved replication mediates may be unstable during chromosome segregation. The chromosomal consequences of these two models are distinct.

**Fig. 7:**
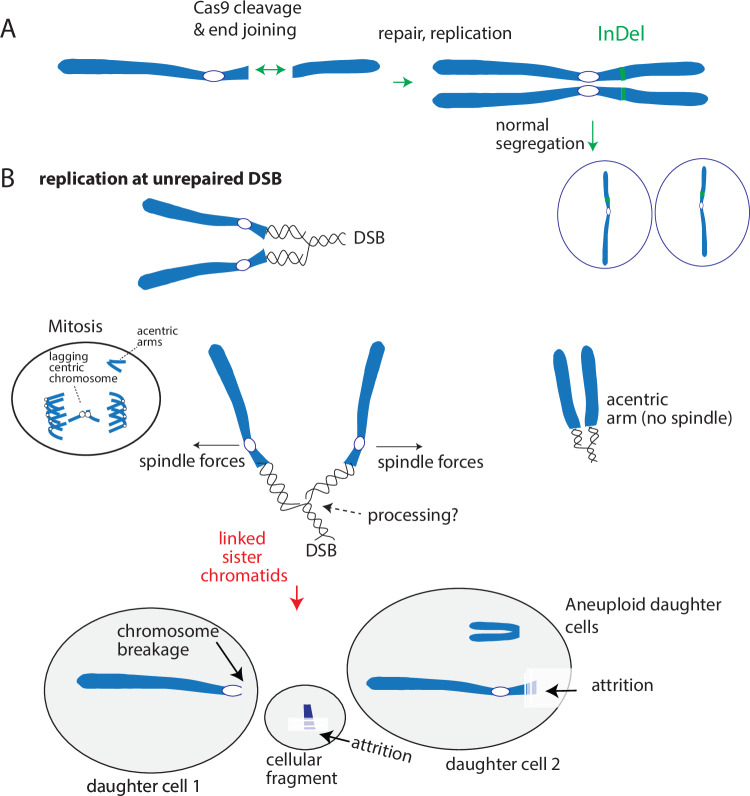
End joining failure results in asymmetric attrition and secondary breakages during cell cycle progression. Model on how an unrepaired DSB results in chromosomal-scale changes centromeric and telomeric to the Cas9-induced cut site. A DNA DSB may be repaired by rejoining the appropriate repair partners centromeric and telomeric of the break, potentially creating a small indel, while restoring the integrity of the chromosome and allowing normal segregation during mitosis (**A**). Alternatively, an inefficient or slow repair leaves some DNA DSBs unrepaired (**B**). Chromosomes with unrepaired breaks cannot be fully replicated and attempts to repair the broken chromatids using the sister will be unsuccessful and may interfere with chromosome segregation. The asymmetric attrition of DNA centromeric but not telomeric to the break site, as well as the lack of corresponding inverted duplications, point to a mechanism that does not involve the fusion of sister chromatids. This failure to repair-breakage model adds an additional route to chromosomal changes, different from the covalent fusion-breakage model, which has broad experimental evidence in more differentiated cells, such as in cancer, where it can result in inverted duplications and amplifications^59,60^.

Centromeric to the Cas9 cut site, we observed secondary breakage at the centromere. This secondary breakage can result in megabase-scale losses, spanning the region between the Cas9 cut site and the centromere. Dicentric chromosomes are known to produce such outcomes^30^. In addition, we also observed attrition of dozens of kb at the break site. Only one of 18 instances showed no attrition centromeric to the Cas9 cut site, and this cell had failed cytokinesis, containing two nuclei. In stark contrast, there was little or no attrition of the DSB end telomeric of the Cas9 cut site. This asymmetry at the Cas9 cut site points to sister chromatid separation of the centric fragment as the enabler of the attrition. End joining of sister chromatid ends should equally protect the position of the DSB ends centromeric and telomeric to the Cas9 cut site. Dicentric chromosomes breaking in mitosis result in inverted duplications, which manifest as complementary duplications and deletions after separation into daughter cells. Though we did observe the losses, complementary duplications were rare, and those that were observed were not linked to a chromosome but present as a disjointed micronucleus or disjointed fragment (e.g., Fig. 4G). Instead of duplications, we also observed disjointed fragments spanning from the attritted break site to the centromere (e.g., Fig. 3B). This suggests that at least some of the chromosomal changes are not mediated by covalent end joining but potentially merely by unreplicated DNA at an unrepaired break, as proposed in the model presented in Fig. 7.

Previous studies have seen chromosome breakage and frequent segmental copy number changes in the human embryo^31^. These data were interpreted as breakage-fusion-bridge cycles according to the McClintock model^32^. However, the interpretation of SNP array and FISH data in interphase cells as breakage-fusion-bridge cycles and ring chromosomes is based on the premise that DSB ends have been repaired, rather than based on direct observation of circular or dicentric chromosomes. Instead, the presence of disjointed chromosome segments within the same cell, or present as an associated fragment and collected with the same cell, is also possible. While both this study and the work by Voet and colleagues^31^ agree regarding an active breakage cycle in the early human embryo, either fusion cycles or non-covalent mechanisms may lead to the destabilization of a chromosome. The latter mechanism arises from the more recent insight that DSB repair is surprisingly inefficient in the early human embryo and that DSBs can remain unrepaired for hours or even days^33^. An unrepaired break may be sufficient to produce chromosome loss. Though we did not observe inverted duplications characteristic of end joining, both of these mechanisms may be active in the early embryo. Interestingly, a recent study in mouse ESCs also found megabase-sized deletions between the Cas9 cut site and the centromere, and many of these events showed inverted duplications, indicating  end joining^34^. A difference between these experimental system is the proximity of the analysis to break induction. In this study, analysis was performed within 1-2 cell cycles after Cas9 injection, while the study in ESCs involved colony expansion.

The presence of an unrepaired DSB in S-phase is detrimental: a replication fork meeting an unrepaired DSB will collapse. Mechanisms to recover the fork cannot rely on an intact parent strand and are typically unproductive or detrimental. The persistence of unreplicated DNA or of recombination intermediates into mitosis can impede chromosome segregation (model in Fig. 7). Though the molecular actors affecting segregation are not known, there are several plausible candidates. Fork reversal at stalled replication forks can result in complex branched structures that are substrates of cleavage, such as by Mus81^35,36^. Furthermore, MRE11 binds to DSB ends, tethering them^37^ and thereby, MRE11 can facilitate the use of the sister chromatid as a template for repair^38^. However, if the break remains unrepaired, MRE11 may also directly contribute to aneuploidies by tethering sister chromatids. Furthermore, the recruitment or stabilization of cohesin at an unrepaired DSB^39^ may impair sister chromatid separation. Interestingly, a DSB can delay DNA replication at or near the break site^40^, resulting in unreplicated DNA. This kind of incomplete replication–breakage cycle may result in the progressive attrition as seen in Figs. 4 and 6. Through stalled replication fork processing, fork reversal, and migration of the recombination junction, repair intermediates may impair segregation at anaphase, resulting in lagging chromosomes and breakage at the site of sister chromatid entanglement near, but not identical to, the Cas9 cut site. Furthermore, secondary centromeric breakage can result. These consequences apply centromeric, but not telomeric of the Cas9 cut site due to the lack of spindle forces on the acentric arm. Such progressive chromosome instability will typically result in the elimination of the affected cell or of the embryo.

Preimplantation genetic testing for aneuploidy, which has been used to identify euploid embryos prior to transfer, has identified spontaneous segmental aneuploidy at appreciable frequencies, constituting a likely cause of developmental failure^31,41–46^. The findings presented here point to several shared features of Cas9-induced and spontaneous aneuploidy: chromosome lagging occurs spontaneously^47^; about 20% of spontaneous chromosome breakages are found at or near the centromere^8^, which may be the secondary consequence of breakage at a fragile site as seen in Fig. 6. Both events lead to chromosome-containing cellular fragments and thereby exclusion of genetic material from the embryo, resulting in a surprisingly similar ratio of chromosomal losses versus gains^8^.

Spontaneous chromosome breakages are not random; they are enriched at fragile sites consistent with a pattern characteristic of DNA replication stress^8,48^. Consistent with earlier findings, we describe new breakages at *WWOX* and *DPP10* and in the intergenic region adjacent to *LINGO2*. Both genes are located at known fragile sites that break spontaneously in tumor cells^49,50^ and have been implicated in developmental disorders of the nervous system^51,52^. Chromosome-containing cellular fragments have previously been described in both human and rhesus macaque embryos^10,53^, albeit without genomic coordinates. The coordinates of chromosome breakage in the early embryo point to difficult-to-replicate gene-poor regions. A plausible mechanism for spontaneous chromosome breakage is the collapse of DNA replication forks^8^. Single-ended DSBs at collapsed forks are not a suitable substrate for end joining due to lack of a partner end. Breakage cycles, as observed in Fig. 6, resulting downstream of replication stress-induced breakage, may thus be the result of unresolved replication intermediates rather than of end joining. An unrepaired Cas9-induced DSB will likewise impede replication completion; such shared molecular features of Cas9-induced and spontaneous breakages could underlie the observed shared patterns of chromosomal consequences. Further mechanistic understanding enabled through CRISPR-Cas-based models of DNA damage may ultimately provide a path to reduce genetic abnormalities, reduce embryo attrition, and improve fertility treatments.

### Limitations of the study

Limitations of this study include the potential presence of other genetic outcomes from CRISPR-Cas9 DSBs, such as complex genomic rearrangements and translocations not seen on SNP arrays or by PCR genotyping the targeted genomic locus. However, these other outcomes would be linked to the abnormality of another chromosome and do not explain single Cas9-induced aneuploidies in otherwise euploid embryos. Though other repair outcomes cannot be excluded, they do not constitute the most common mechanism for Cas9-induced chromosomal aneuploidies. Long read sequencing may potentially capture additional uncommon events. A limitation of SNP array-based attrition mapping is that it resolves the break site to the kilobase rather than base-pair level. However, as attrition events occur on a kilobase scale, this resolution is adequate for most analyses. Base-pair resolution was achieved in one sample (Fig. 3d–f) using PCR, which additionally permitted assessment of sister chromatid end joining—the result of which was negative. Lastly, the CRISPR-Cas9 activity in human embryos may have resulted in off-target indels, which were not evaluated systematically in this study.

## Methods

### Research samples

#### Human embryos

The Columbia University Institutional Review Board (IRB) approved all research involving human embryos, including the use of Cas9 to study DNA repair and the sharing of array-based genotyping data. The study was reviewed and approved by the embryonic stem cell research committee and the IRB in accordance with International Society for Stem Cell Research and American Society for Reproductive Medicine guidelines and with applicable laws^54,55^. No financial or other form of compensation was used for this study to obtain research samples. A potential requirement to recontact donors to reconsent for the specific research of the study, or to provide results of the study, was waived.

Cryopreserved human 2PN embryos were anonymously donated by couples who considered their fertility treatment complete and who provided informed consent for use of embryos in research. Embryos were cryopreserved between the years 2007 and 2012 using One Step (PB1, PG, 1 M sucrose) cryopreservation solution, Sage embryo freeze kit, or Quinn’s embryo freeze kit. Embryos were stored in liquid nitrogen until use. All samples were de-identified.

For the experimental procedures, embryos were thawed, then exposed to experimental conditions as outlined below. No blinding was applied. 2PN embryos are typically frozen on day 1. The timing from injection to mitosis is expected to be variable due to the variability in the duration of the first cell cycle and due to an unknown timing of freezing. All experiments were conducted during incubation at 37 °C with 5% CO_2_ and 20% O_2_. All human embryos were cultured for no more than 1–6 days, in accordance with the internationally accepted standards at the time of the conduct of the research, to limit progression to less than 14 days^36^. Experiments were done in groups of 5–10 embryos, initially to observe a biological signal, and then to increase numbers until statistical tests were supported by sample size.

### Method details

#### RNP preparation

2 nmol of sgRNAs were obtained from Integrated DNA Technologies (IDT) with the sequence listed in Supplementary Data 1 and dissolved in 20 μl to a concentration of 100 μM sgRNA. For RNP preparation, 3 μL of injection buffer, 2 μL of 63 μM IDT nlsCas9 v3, and 1.5 μL of 100 μM sgRNA were combined and incubated at room temperature for 5 min. Thereafter, 96.5 μL of an injection buffer was added. The injection buffer consists of 5 mM Tris-HCl, 0.1 mM EDTA, pH 7.8. The RNP solution was then centrifuged at 16,000 × *g* (RCF) for 2 min prior to loading into the injection needle and cytoplasmic injection.

#### Embryo manipulations

Embryo manipulations were performed in an inverted Olympus IX71 microscope using Narishige micromanipulators on a stage heated to 37 °C. Frozen 2PN (day 1) embryos were thawed using the One Step or Sage Embryo thaw kit, and pronucleus formation was confirmed. Embryo polar bodies were collected whenever possible. RNP was prepared as above. The tip of an injection needle was nicked, and small, visible amounts (~ 50 fL) of the Cas9 RNP were injected manually into the cytoplasm of thawed embryos using a Narishige micromanipulator. Embryos were then cultured in Global Total (Cooper Surgical) in an incubator (Thermo Scientific, Heracell 150i) at 6% CO_2_, 37 °C until collection.

#### Genome amplification and genotyping

Single blastomeres were collected on day 2 to day 3, or if indicated, on day 4, on the heated stage of an inverted Olympus IX71 microscope equipped with Narishige micromanipulators and a zona pellucida laser (Hamilton-Thorne). Trophectoderm biopsies were obtained on day 6 of development using 300 ms laser pulses to separate the trophectoderm from the inner cell mass. All samples were placed in single tubes with 2 or 4 μL of PBS. Amplification was performed using the REPLI-g single kit (Qiagen) according to the manufacturer’s instructions, using either a half reaction for 2 μL or a full reaction for 4 μL.

Genotyping was performed using primers for amplification and sequencing as listed in Supplementary Data 5. PCR was performed using AmpliTaq Gold. PCR products were run on a 1.5% agarose gel for visual inspection of product size and submitted to Genewiz for Sanger sequencing. Base changes were analyzed at the region of Cas9 target sites using SnapGene2 and ICE analysis (Synthego). Unprocessed gel images are available in the Source Data.

#### Genome-wide SNP array

Embryo biopsies were amplified at Columbia University using the REPLI-g single cell kit. according to the manufacturer’s instructions. Copy number and genotyping analysis was performed using gSUITE software (Genomic Prediction). For copy number analysis, raw intensities from the Affymetrix Axiom array are first processed according to the method described in ref. ^56^. After normalizing with a panel of normal males and females, the copy number is then calculated for each probeset. Normalized intensity is displayed. Mapping of endogenous fragile sites was done through visual evaluation of LOH. Breakpoints were mapped to chromosomal bands by visual analysis of SNP array chromosome plots, including analysis of both copy number signal and heterozygosity calls. The accuracy of mapping is between 100 and 500 kb. A segmental error was defined as the gain or loss of a chromosome arm or segment. Fragmented chromosomes were called if there were multiple break points within a given chromosome. Chromosomal coordinates were mapped using probe intensity data on samples where chromosomal changes included nullisomy or a difference of at least two copies.

#### Live-cell imaging with chromosomal analysis

2PN zygotes were thawed and injected as described above and incubated in SPY555-DNA or SPY650-DNA (Spirochrome) diluted at 1:2000. Zygotes were injected and transported in 1.5 ml cryovials containing 1 ml Global Total pre-equilibrated in 5% CO_2_ in a portable incubator (BioTherm, Cryologic, BT-RB1) warmed to 37 °C. Zygotes were placed in 1 μl droplets of the pre-equilibrated medium covered with Paraffin Oil (CooperSurgical) and mounted in a light-sheet microscope equipped with environmental temperature and gas controls (TruLive3D, Bruker/Luxendo). The imaging chamber was maintained at 37 °C and 5% CO_2_ throughout the entire imaging session. Either a 561 nm laser or a 642 nm laser was used to generate the light sheet at 10% intensity with a 10% attenuator, which is equivalent to ~1% laser intensity. Z-stack was set at 2 μm interval covering 200 μm total, and the exposure time was 120 ms for each slice. Pixel size was 0.208 μm with 31.25× detection objective. Images were acquired every 5–15 min. From initiating transfer to imaging required ~1.5 h. Staining, transport, preparation, and imaging from day 1 to day 3 did not impair developmental progression or genome stability after optimization of conditions (Supplementary Movie 5). All images were deconvolved using an empirical point spread function calculated from imaging Fluoresbrite® Multifluorescent Microspheres 0.20 µm (Polysciences) on the microscope. As cumulus cells around the embryo were overly bright, some cumulus cells were segmented using a simple machine-learning-based segmentation pipeline in ilastik (https://www.ilastik.org/), then subtracted from the raw image. Movies were generated from simple maximum intensity projection of each frame.

After two days, embryo samples were collected, transported at room temperature by City Bike to the Columbia Medical Campus, dissociated, and used for chromosomal analysis. Initial attempts to use Phi-29 DNA polymerase-based MDA methods failed to yield amplification and signal on the Affymetrix SNP array assay. Modification of the protocol to include a heating step prior to MDA was successful but not sufficiently reliable, yielding results for only a subset of cells. A total of 37 2PN zygotes were thawed, of which 28 survived the thaw and injections. Karyotyping was successful for 13 embryos using barcoded whole genome amplification, which uses extension of random barcoded primers using Klenow, followed by a PCR amplification step^57,58^.

#### DNA FISH

Heterozygous *EYS* delC/wt ESCs were used due to the paternal allele containing a delC mutation compared to the wt maternal allele^3^. Thus, DSBs were only induced on the paternal allele. Heterozygous *EYS* c.6794del (“delC”; p.Pro2265fs) embryonic stem cells (ESCs) were cultured on Geltrex Matrix (ThermoFisher Scientific)-coated plates in the StemFlex Medium (ThermoFisher Scientific). Cells were harvested with TrypLE Express Enzyme (ThermoFisher Scientific) and counted on the EVE Automatic Cell Counter (NanoEntek). 5 × 10^6^ cells were used for each nucleofection reaction, together with CRISPR-Cas9 RNP assembled after combining 30 μg Alt-R S.p. Cas9-GFP V3 protein (IDT) and 200 pmol *EYS* gRNA (IDT). Nucleofections were performed using SF Cell Line 4D-Nucleofector X Kit L (Lonza, Cat. No. V4XP-3024), the 4D-Nucleofector System (Lonza), and program CA-137. After nucleofections, cells were plated in Geltrex-coated 6-well culture plates containing StemFlex medium and ROCK inhibitor Y-27632 (Selleck Chemicals, Cat. No. S1049). After 18 h, cells were collected for fluorescence-activated cell sorting (Influx Cell Sorter) of Cas9-GFP-positive cell populations^31^. GFP-positive *EYS* ES cells were prepared according to the sample harvest protocol provided by Memorial Sloan Kettering Cancer Center’s Molecular Cytogenetics Core Facility. Cells were centrifuged for 5 min at 1200 rpm in Eppendorf tubes and resuspended in 1–2 mL pre-warmed 75 mM KCl. Samples were incubated at 37 °C for 8–9 min, ensuring swelling time never exceeded 10 min, and were then centrifuged at 1500 rpm for 8 min. KCl supernatant was removed, and 750 µL of freshly prepared fixative containing methanol and glacial acetic acid in a 3:1 ratio was added to resuspend the cell pellet. Samples were kept at room temperature for 15 min. Samples were centrifuged for 5 min at 5000 rpm, supernatant was removed, and cell pellets were resuspended again using 750 µL fixative and stored at −20 °C prior to DNA FISH hybridization. 20 µl of ES cell suspension was dropped on superfrost slides and air-dried at RT for one hour. Slides were kept in a dry environment at RT for 24 h before probe hybridization. The next day, prepared slides were equilibrated in 2 × SSC briefly, followed by dehydration in graded ethanol. 10 µl of prewarmed *EYS* break-apart (EYSBA) FISH probes (Empire Genomics; EYSBA-20-REGR), consisting of two short ssDNA sequences flanking either side of the *EYS* gene (coordinates within which two probes locate: chr6:63,720,136–65,707,214; hg38), were prepared. The probe proximal to the centromere was red; the probe distal was green. EYSBA FISH probes were hybridized to prepared slides at 37 °C overnight. Subsequently, samples were washed in 0.4× SSC/0.3% tween20 at 73 °C for two minutes, followed by another one-minute wash in 2 × SSC/0.1 Tween20 at RT. After a briefly rinse in 2× SSC at room temperature, cells were stained with 5 μg/ml DAPI/PBS for 5 min, and slides were mounted for imaging. The mounted slides were imaged using a Zeiss AxioObserver.Z1 (Zeiss, Germany) using a 63×/1.4 NA objective. 100 positions were evenly distributed across the slide, and 6.74 µm z-stacks were set up with a 0.24 µm step size at each position. Imaged slides were evaluated using Zeiss Zen Imaging software. Z-stacks for each cell image were analyzed for FISH probe signals. After identifying cells where at least two break-apart FISH probe signal pairs were present, distances between the probes centromeric and telomeric of the targeted *EYS* gene locus were measured.

#### Measuring allelic depression due to DSBs at the EYS locus

Heterozygous *EYS*delC/wt embryonic stem cells derived in a previous study^3^, were cultured under feeder-free conditions on Geltrex-coated plates in StemFlex medium (ThermoFisher Scientific, Cat. No. A3349401). For nucleofection, 6 × 10⁶ cells were used to provide ≥ 5 × 10⁵ cells per datapoint. Cas9 RNP complexes were assembled immediately before use by combining 5 µL SpCas9 Nuclease V3 (10 µg/µL; IDT, Cat. No 1081059) with 10 µL total gRNA (20 µg; IDT, Alt-R™ CRISPR-Cas9 gRNA) diluted in IDTE Buffer (10 mM Tris-HCl, 0.1 mM EDTA, pH 7.5). RNP complexes were incubated for 15 min at room temperature. Cells were pelleted at 1300 rpm for 4 min, the supernatant removed, and resuspended in 100 µL P3 Primary Cell Nucleofector Solution (Lonza, Cat. No. V4XP-3032; 82% P3 buffer + 18% supplement, v/v). The 100 µL cell suspension was then added to the preassembled RNP mixture, gently mixed, and transferred into a 100 µL Nucleocuvette Vessel. Nucleofection was performed using the Amaxa 4D-Nucleofector System with program CA-137. Immediately after nucleofection, cells were plated into two Geltrex-coated 6-well plates containing StemFlex medium supplemented with 10 µM Y-27632 ROCK inhibitor. Cells were harvested by TrypLE dissociation and centrifugation for genomic DNA extraction and PCR (primer sequences in Supplementary Data 7).

Genomic DNA was isolated using the DNeasy Blood & Tissue Kit (QIAGEN, Cat. No. 69504). Amplicons flanking the Cas9 cleavage site at *EYS* were amplified using high-fidelity PCR and submitted for Amplicon-EZ targeted sequencing (Genewiz). The relative abundance of the paternal allele was quantified by loss of the *EYS* c.6794del mutation paternal SNP across time points.

#### Quantification and statistical analysis

Statistical analysis was performed using Fisher’s exact test (two-sided) as indicated. A *p*-value of less than 0.05 was considered significant.

### Reporting summary

Further information on research design is available in the Nature Portfolio Reporting Summary linked to this article.

## Supplementary information

Supplementary Information

Description of Additional Supplementary Files

Supplementary Movie 1

Supplementary Movie 2

Supplementary Movie 3

Supplementary Movie 4

Supplementary Movie 5

Supplementary Movie 6

Supplementary Data 1

Supplementary Data 2

Supplementary Data 3

Supplementary Data 4

Supplementary Data 5

Supplementary Data 6

Supplementary Data 7

Reporting Summary

Transparent Peer Review file

## Source data

Source Data

## Data Availability

SNP array data have been deposited in NCBI’s Gene Expression Omnibus and are accessible through GEO Series accession number GSE186407. Low-pass whole-genome sequencing data are available from NCBI’s Sequence Read Archive (SRA) under accession number PRJNA1354093. Data from clinical samples (Fig. 6) is not available due to human subjects’ protection. Source data are provided with this paper.
