# Supplementary material for: Asymmetric attrition and secondary chromosome destabilization after double-strand breaks in human embryonic development
Source: Nat Commun. 2026 Jun 3;17:7140. doi: 10.1038/s41467-026-73891-7 (PMC13396663; doi:10.1038/s41467-026-73891-7)
Supplement: Supplementary file 1 — Supplementary Information [file 41467_2026_73891_MOESM1_ESM.pdf]

Supplementary Figure 1

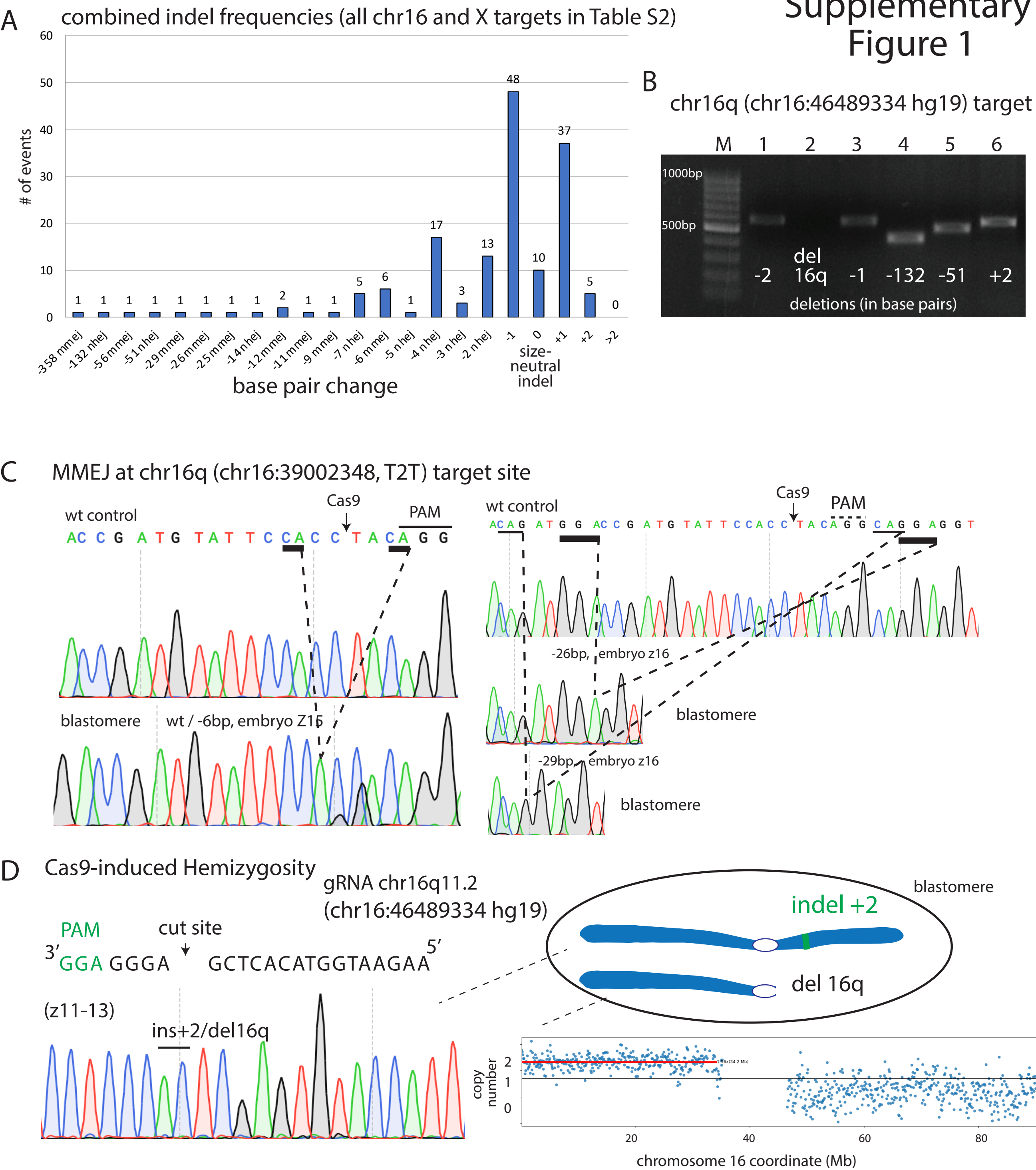

Supplementary Figure 1 | Quantification of indels in human embryos.

A) Quantification of combined indel frequencies at the pericentromeric target sites on chromosomes 16 and X according to Supplementary Data 2. MMEJ, microhomology-mediated end joining events. NHEJ, non-homologous end joining. **B**) Gel electrophoresis demonstrating representative examples of different PCR outcomes at the chromosome 16q location after injection of CRISPR-Cas9 and gRNA targeting chr16:46455422. Different sizes of PCR products show Cas9-generated indel formation at the target site. The failure to amplify, as seen for sample 2, represents complete allelic dropout after Cas9 cleavage. Cell IDs are from three different embryos: 18_Z12, 20_Z13, 16_Z12, 15_Z12, 14_Z11, 13_Z11 (Supplementary Data 2). **C**) Examples of MMEJ at gRNA target site (chr16:35126552) in zygote 15 (z15) and zygote 16 (z16). Regions of microhomology are underlined and the combination pattern indicated with dotted lines. **D**) Left panel: Sanger sequencing analysis of a blastomere, revealing a single indel with an insertion of two nucleotides (AC). Right panel: Chromosomal constitution in the same cell represented by SNP array probe intensity plot. Loss of the q arm results in loss of heterozygosity for any PCR across the break site, and telomeric to it.

Supplementary Figure 2

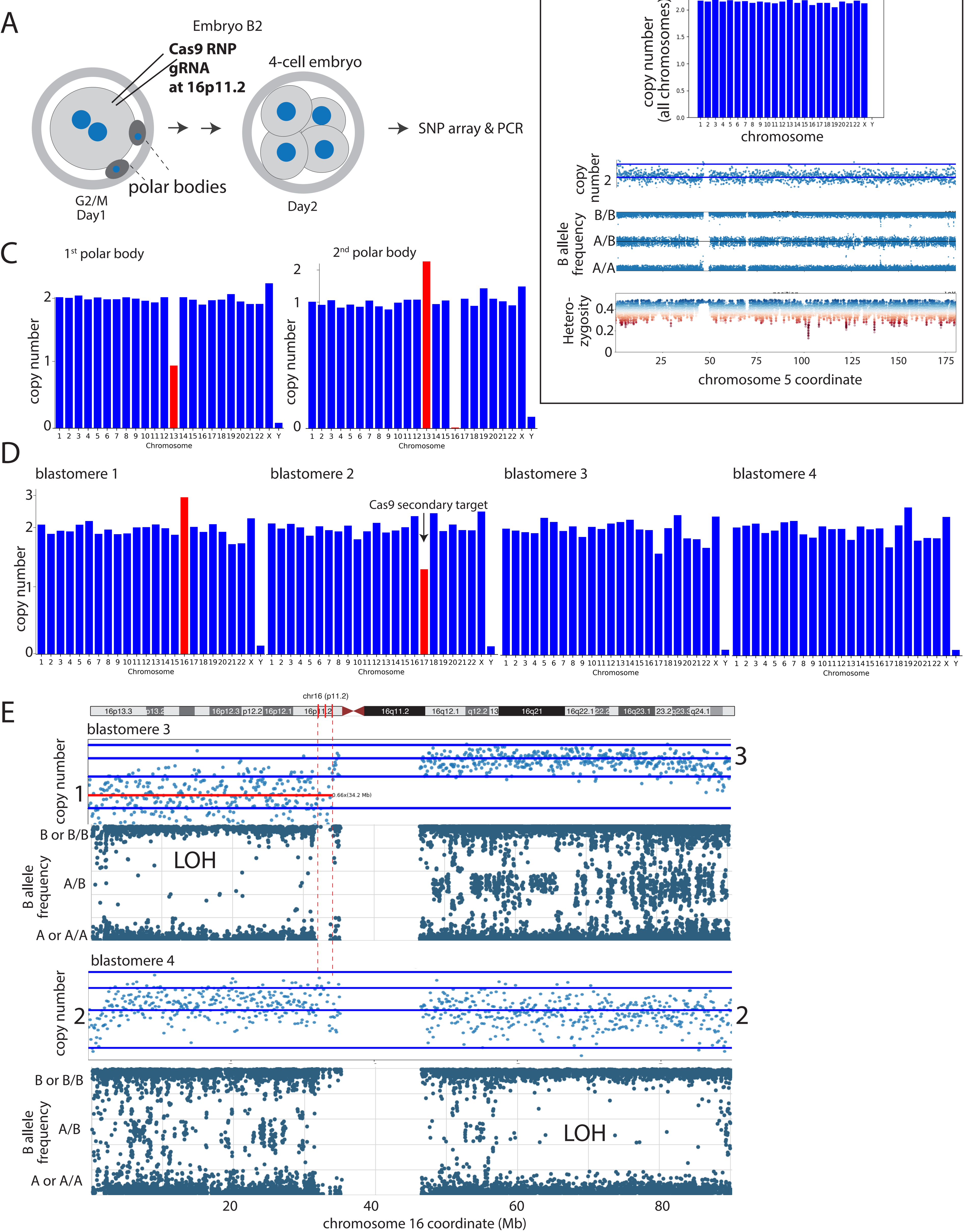

Supplementary Figure 2 | Mosaic correction of a trisomy 16 embryo.

A) Schematic of the experiment showing a female fertilized zygote with the polar body 1 and polar body 2 removed for genotyping. A single gRNA targeting 3 sites with identical sequence on chromosome 16p at chr16:33721043, chr16:32003016, and chr16:32946546 (hg19). After injection of Cas9 RNP, the zygote was allowed to develop to the four-cell stage and individual blastomeres were further analyzed using SNP array and PCR. Cas9 cleavage is expected to occur at the 2-cell stage after nuclear envelope breakdown and transition through mitosis. **B**) Example of normal copy number profiles through SNP array analysis using a female cell line. Upper panel: Bar diagram shows normal copy number across the genome for a male embryo (46, XX). Lower panel: An even copy number profile shown here for chromosome 5, demonstrating lack of segmental errors. B and A allele are randomly assigned designations for SNP variants. Two A alleles lead to B allele frequency (BAF) 0, two B alleles lead to BAF = 1, and heterozygous A/B alleles lead to BAF = 0.5. The homozygosity value is calculated as a moving average of absolute value (0.5-BAF). The closer to 0.5 (e.g., BAF = 1), the more consistently homozygous the region is (blue color). Red colored datapoints below ~0.4 show heterozygosity. BAF plots and homozygosity plots serve as validating datapoints for copy number profiles. In embryos, the data generated from single cells are typically noisier than in data from cell lines or blastocyst biopsies, and heterozygosity deviates from 0.5, because of amplification bias from single cells. **C**) Bar diagrams showing copy number analysis across the genome in two polar bodies. Note the loss of one chromosome 13 in polar body 1 (left panel), and the gain in polar body 2 (right panel), resulting in a normal chromosome 13 complement in the embryo; an example of a spontaneous meiotic correction. In contrast, chromosome 16 is absent in polar body 2, resulting in a gain in the embryo. **D**) Copy number analysis of all four blastomeres collected on day 2 of development, showing all chromosomes in bar diagrams. Note the normal copy number of chromosome 13, and mosaic correction of trisomy 16: Blastomere 3 and 4 have an apparently normal copy number of chromosome 16. **E**) Copy number

analysis of chromosome 16 in blastomeres 3 and 4. Blastomere 4 has a normal copy number, albeit with LOH of 16q, while blastomere 3 has imbalanced chromosome arms.

Supplementary Figure 3

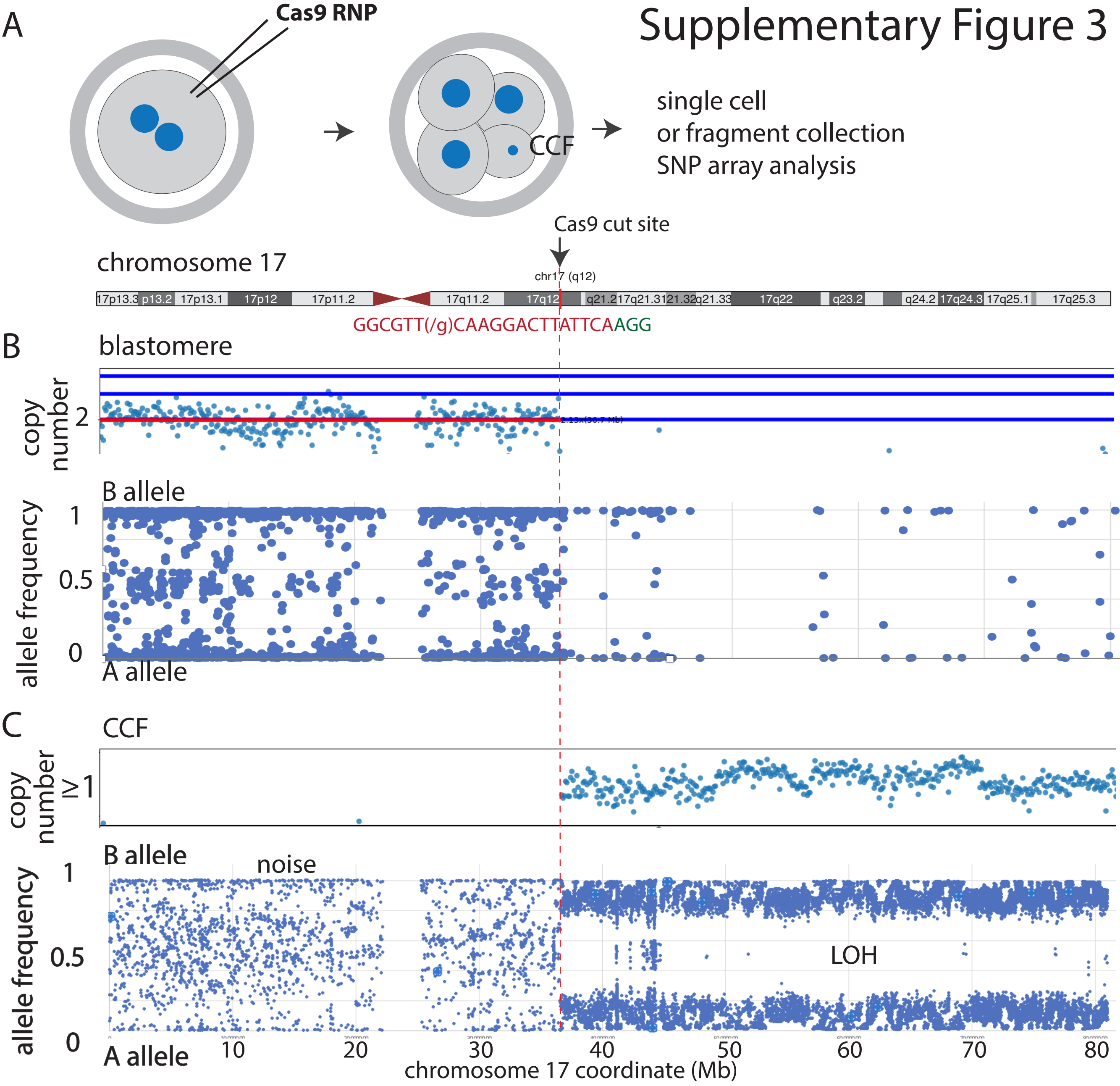

Supplementary Figure 3 | Exclusion of chromosomal segments in a cytoplasmic chromosome-containing fragment.

A) Schematic of the experiment showing CRISPR-Cas9 RNP injection into human 2PN zygote that develops into an embryo with 3 blastomeres and a chromosome containing fragment (CCF). Shown below is the location of an off-target site on chromosome 17 for the gRNA used in the experiment. **B, C**) Copy number and allele frequency plots showing reciprocal chromosome breakage in a blastomere (**B**) and a CCF (**C**) collected from the same embryo. The blastomere is heterozygous for two copies of the chromosome 17 p arm, while the cytoplasmic fragment is uniparental with loss of heterozygosity across the entire p arm. LOH, loss of heterozygosity.

Supplementary Figure 4

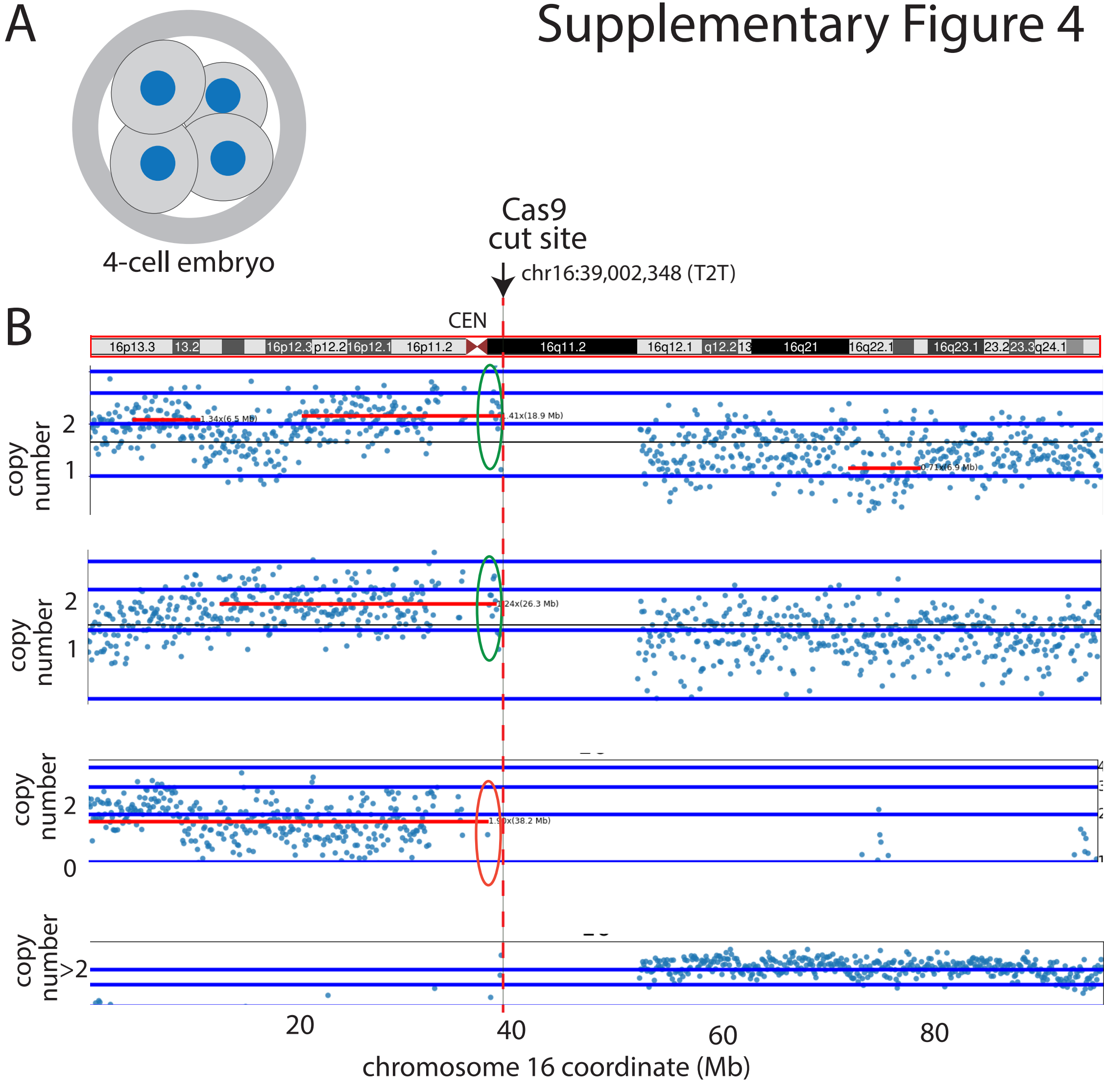

Supplementary Figure 4 | Position of the gRNA relative to the centromere can validate genome annotation.

A) Four-cell human embryo for which single cells and a fragment were analyzed using SNP arrays and copy number plots. Injection of Cas9 RNP with gRNAs targeting the indicated sites was performed at the 2PN stage.

B) SNP array copy number analysis of each of the four blastomeres. Vertical dotted red line indicates gRNA target site. Two blastomeres show a loss of one copy of the 16 q arm. Both blastomeres show no attrition or gain between the Cas9 cut site and the centromere (green ovals).

One cell (bottom) shows a centric arm with attrition of SNPs between Cas9 cut site and the centromere. The red oval indicates attrition of genomic DNA between the Cas9 cut site and the centromere. A fourth cell is an acentric 16q fragment.

Supplementary Fig. 5

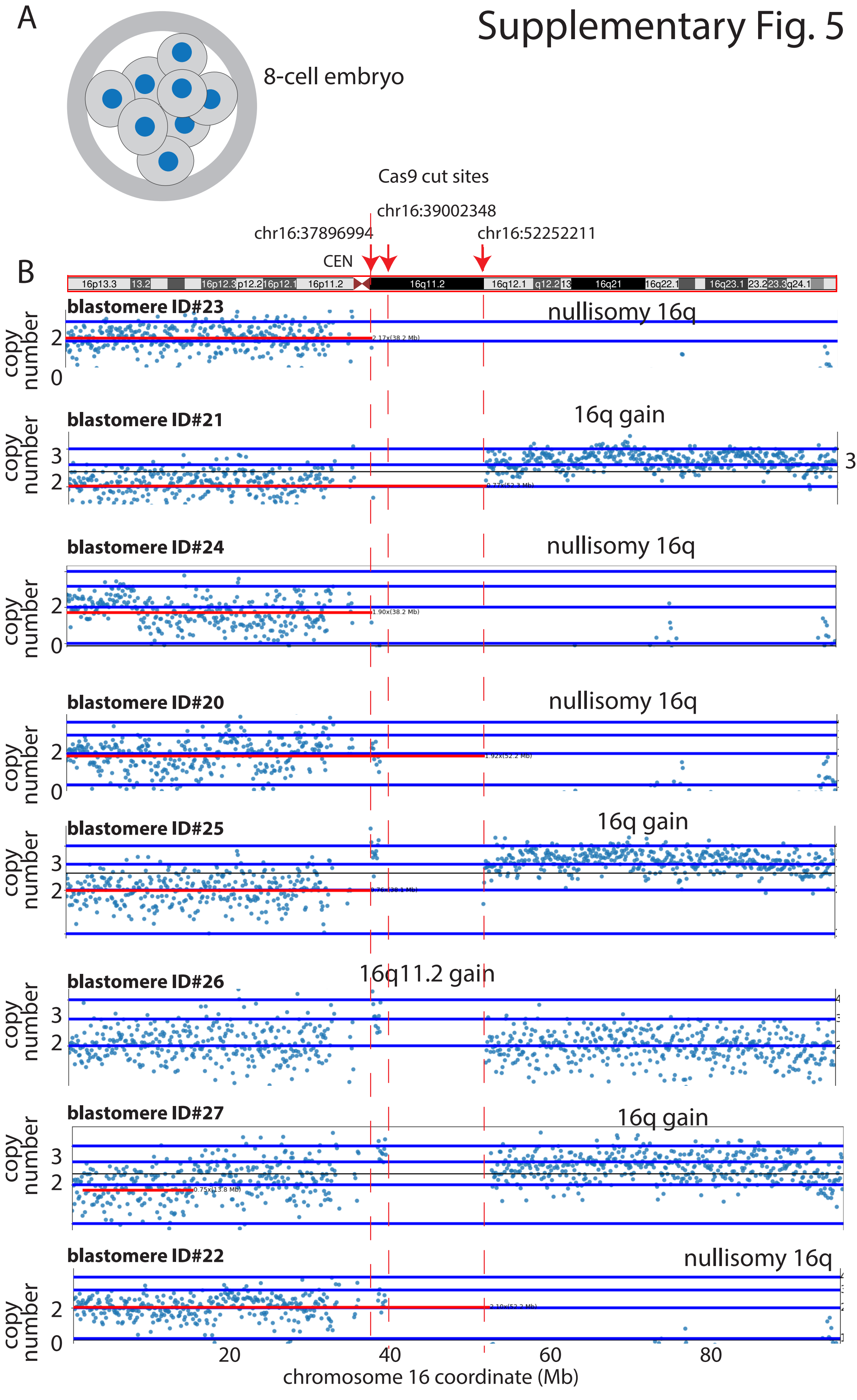

Supplementary Figure 5 | Pericentromeric cleavage results in copy number changes between Cas9 cut site and the centromere.

A) Eight-cell human embryo for which eight single cells were analyzed using SNP arrays and copy number plots. The embryo was injected with CRISPR-Cas9 RNP with three gRNAs targeting three different sites (vertical red lines) on chromosome 16, all at pericentromeric q arm at the 2PN stage.
B) SNP array copy number analysis of individual blastomeres for said eight-cell embryo (ID: z13). Complementary losses and gains of the chromosome 16 q arm are apparent. All chromosomal changes occur on the q arm, while the centromere-containing p-arm is present in two copies in each cell.

Supplementary Fig. 6

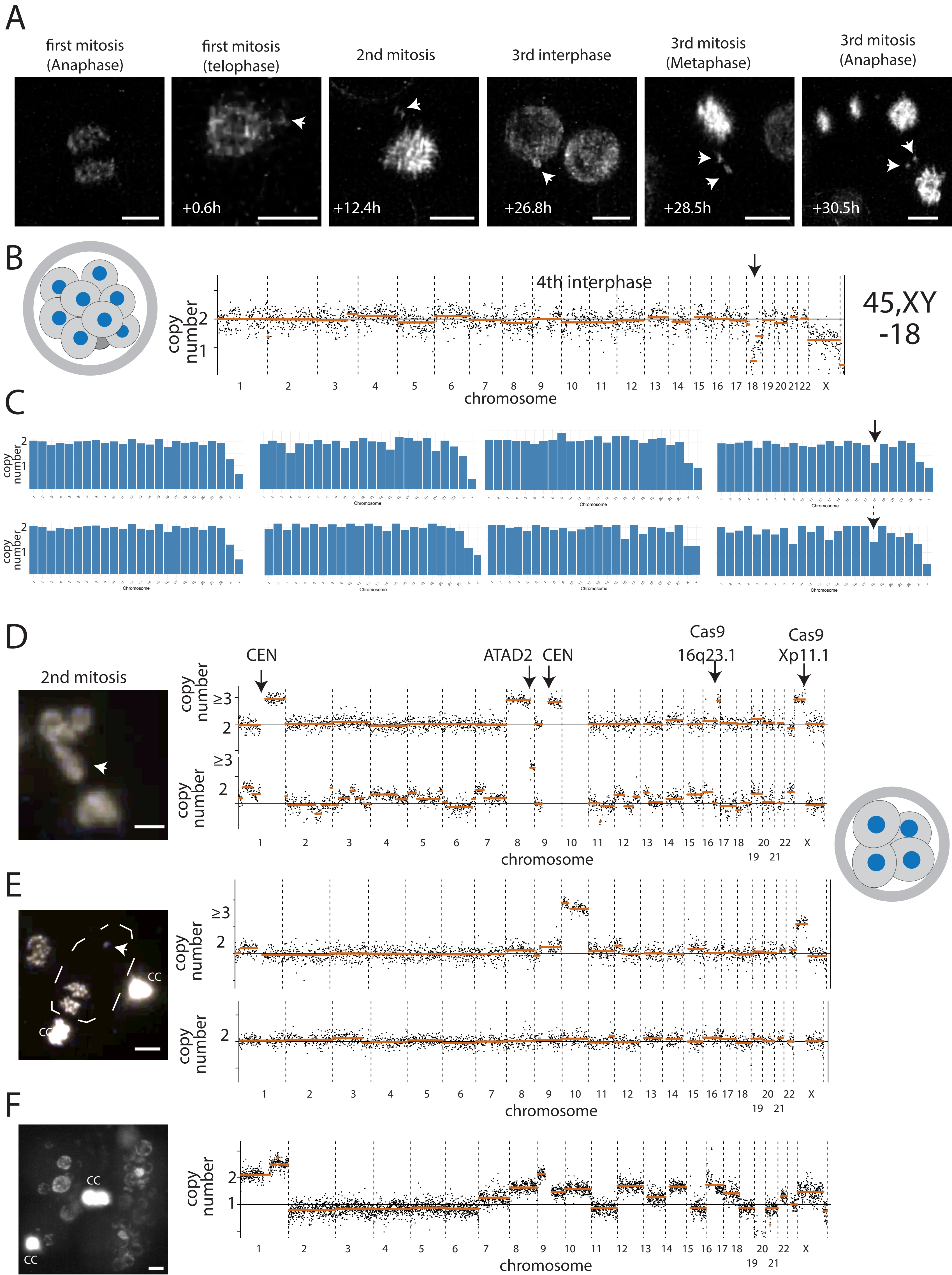

Supplementary Figure 6 | Live-cell imaging of chromosomal aneuploidies.

A) Imaging from the first mitosis ~10-12h post injection of an embryo to the eight-cell stage, showing the origin of a chromosome 18 aneuploidy. **B**) Karyotype plot for a single blastomere collected from the eight-cell stage human embryo on day 3 of development. Loss of chromosome 18 is indicated by an arrow. **C**) Chromosomal content of all eight cells collected from a day 3 human embryo. Arrow points to chromosome loss. The dashed arrow is a likely loss (the quality of data from the last cell on lower row was suboptimal). **D, E**) Four-cell embryo at the second mitosis with Cas9-induced and spontaneous abnormalities. Two images of second mitosis are shown with corresponding chromosome content in daughter nuclei. Panel (**D**) shows abnormal anaphase of a chaotic aneuploidy composed of both spontaneous and Cas9-induced breakages and panel (**E**) shows two additional cells of the same four-cell embryo, with the dashed line indicating the approximate outline of dividing two-cell blastomere. CC, cumulus cell. **F**) Example of an embryo with multinucleation from chaotic first and second cell division with numerous micronucleations. Karyotype of a single blastomeres (of four cells total) is shown, others are equally aneuploid (**Supplementary Data 5**). Note both whole chromosome aneuploidy as well as breakages on chromosomes 1, 9, and X. Scale bars, 10 μm.

Supplementary Movies

Supplementary Movie 1 | First mitosis until the two-cell stage. Related to **Figure 4A-C**.

Supplementary Movie 2 | First mitosis until the two-cell stage. Related to **Figure 4D, E**.

Supplementary Movie 3 | Development from the zygote to the eight-cell stage on day 3. The movie is a combination of three recording segments. Related to **Figure 4F-L**.

Supplementary Movie 4 | Development from the zygote to the eight-cell stage on day 3. Related to **Figure S6**.

Supplementary Movie 5 | Cell division with multinucleation and ensuing genome instability.

Supplementary Movie 6 | Normal developmental progression from the zygote to the eight-cell stage on day 3 without aneuploidies and with normal developmental kinetics. Control for culture conditions, staining and imaging. Related to **Supplementary Data 5**, embryo 4.

Supplementary Data Tables

Supplementary Data 1. sgRNA chromosome targets and sequences.

* Based on hg19 and hg38 annotation, this position is on the p arm of chromosome 16. Cleavage with this sgRNA shows the functional centromere is proximal to this site.

** Multiple secondary target sites with perfect match and a tolerated SNP on chr17:38487370, chr16:31991695, and chr16:32935225.

Bold: Pam site.

The activity of gRNA Chr16 centromere is compromised because of a SNP mismatch (see Table S2) and was thus used only in a single experiment or only 5/27 embryos, and data were not included in the indel efficiency analysis. The gRNA is relevant however to Fig. S5.

Supplementary Data 2. Results of SNP array analysis and on-target Sanger sequencing analysis of embryonic cells after Cas9 cleavage.

Indels at the targeted genomic sequence generated by Cas9 RNP with gRNA, based on Sanger sequencing. Samples with two complete homologous targeted chromosomes can have two different indels. Indels on maternal and paternal chromosome are separated by a dash (n/n) and indels where two chromosomes are present based on SNP arrays but no heterozygosity is detectable are indicated by double asterisk (**, n=22 instances). These may be two homozygous indels or only one of the two is detectable by PCR and Sanger sequencing. A size-neutral indel refers to a nucleotide insertion and deletion of equal value resulting in a net zero indel. Detailed chromosomal content based on SNP array analysis.

Polar bodies were used to evaluate meiotic aneuploidy.

A gRNA targeting chr16 centromere (hg38, chr16c:36221387, italics in column K and L) was excluded from indel efficiency analysis in Fig. 1B because of a frequent mismatch compromising its activity.

Supplementary Data 3. Analysis of cells or fragments.

Blastomere-like refers to the characterization at time of collection, meaning of adequate stage-specific size. These blastomeres-like cells may nevertheless have a low and abnormal or even no chromosome content. Some blastomere-like cells and fragments contain chromosomal content, while others failed amplification presumably because of a lack of genomic DNA. Other fragments contain spontaneous chaotic aneuploidy or haploidy. These were not included in the sample count of Fig. 1B but are included in Fig. 1F.

Supplementary Data 4. Mapping of Cas9-induced break points as well as spontaneous chromosome breakage.

Mapping was performed on samples with copy number transitions of 0-1 or greater, and 1-3 or greater. Samples with multiple gRNA targets on the same chromosome were not included in the calculation of DNA loss flanking the cut site.

Supplementary Data 5. Chromosomal analysis after live-cell imaging.

Table related to **Figure 4** and **Fig. S6**.

Supplementary Data 6. Blastocyst mosaic chromosomal breakages.

Table related to **Figure 6**.

Supplementary Data 7. Primer sequences used for genotyping.

Genomic coordinates according to hg38.
