# Supplementary material for: Asymmetric attrition and secondary chromosome destabilization after double-strand breaks in human embryonic development
Source: Nat Commun. 2026 Jun 3;17:7140. doi: 10.1038/s41467-026-73891-7 (PMC13396663; doi:10.1038/s41467-026-73891-7)
Supplement: Supplementary file 2 — Description of Additional Supplementary Files [file 41467_2026_73891_MOESM2_ESM.pdf]

Supplementary Movies

Supplementary Movie 1 | First mitosis until the two-cell stage. Related to **Figure 4A-C**.

Supplementary Movie 2 | First mitosis until the two-cell stage. Related to **Figure 4D, E**.

Supplementary Movie 3 | Development from the zygote to the eight-cell stage on day 3. The movie is a combination of three recording segments. Related to **Figure 4F-L**.

Supplementary Movie 4 | Development from the zygote to the eight-cell stage on day 3. Related to **Figure S6**.

Supplementary Movie 5 | Cell division with multinucleation and ensuing genome instability.

Supplementary Movie 6 | Normal developmental progression from the zygote to the eight-cell stage on day 3 without aneuploidies and with normal developmental kinetics. Control for culture conditions, staining and imaging. Related to **Supplementary Data 5**, embryo 4.

Supplementary Data Tables

Supplementary Data 1. sgRNA chromosome targets and sequences.

* Based on hg19 and hg38 annotation, this position is on the p arm of chromosome 16. Cleavage with this sgRNA shows the functional centromere is proximal to this site.

** Multiple secondary target sites with perfect match and a tolerated SNP on chr17:38487370, chr16:31991695, and chr16:32935225.

Bold: Pam site.

The activity of gRNA Chr16 centromere is compromised because of a SNP mismatch (see Table S2) and was thus used only in a single experiment or only 5/27 embryos, and data were not included in the indel efficiency analysis. The gRNA is relevant however to Fig. S5.

Supplementary Data 2. Results of SNP array analysis and on-target Sanger sequencing analysis of embryonic cells after Cas9 cleavage.

Indels at the targeted genomic sequence generated by Cas9 RNP with gRNA, based on Sanger sequencing. Samples with two complete homologous targeted chromosomes can have two different indels. Indels on maternal and paternal chromosome are separated by a dash (n/n) and indels where two chromosomes are present based on SNP arrays but no heterozygosity is detectable are indicated by double asterisk (**, n=22 instances). These may be two homozygous indels or only one of the two is detectable by PCR and Sanger sequencing. A size-neutral indel refers to a nucleotide insertion and deletion of equal value resulting in a net zero indel. Detailed chromosomal content based on SNP array analysis.

Polar bodies were used to evaluate meiotic aneuploidy.

A gRNA targeting chr16 centromere (hg38, chr16c:36221387, italics in column K and L) was excluded from indel efficiency analysis in Fig. 1B because of a frequent mismatch compromising its activity.

Supplementary Data 3. Analysis of cells or fragments.

Blastomere-like refers to the characterization at time of collection, meaning of adequate stage-specific size. These blastomeres-like cells may nevertheless have a low and abnormal or even no chromosome content. Some blastomere-like cells and fragments contain chromosomal content, while others failed amplification presumably because of a lack of genomic DNA. Other fragments contain spontaneous chaotic aneuploidy or haploidy. These were not included in the sample count of Fig. 1B but are included in Fig. 1F.

Supplementary Data 4. Mapping of Cas9-induced break points as well as spontaneous chromosome breakage.

Mapping was performed on samples with copy number transitions of 0-1 or greater, and 1-3 or greater. Samples with multiple gRNA targets on the same chromosome were not included in the calculation of DNA loss flanking the cut site.

Supplementary Data 5. Chromosomal analysis after live-cell imaging.

Table related to **Figure 4** and **Fig. S6**.

Supplementary Data 6. Blastocyst mosaic chromosomal breakages.

Table related to **Figure 6**.

Supplementary Data 7. Primer sequences used for genotyping.

Genomic coordinates according to hg38.
