# Supplementary figures and images for: Asymmetric attrition and secondary chromosome destabilization after double-strand breaks in human embryonic development
Source: Nat Commun. 2026 Jun 3;17:7140. doi: 10.1038/s41467-026-73891-7 (PMC13396663; doi:10.1038/s41467-026-73891-7)

Figure 2D

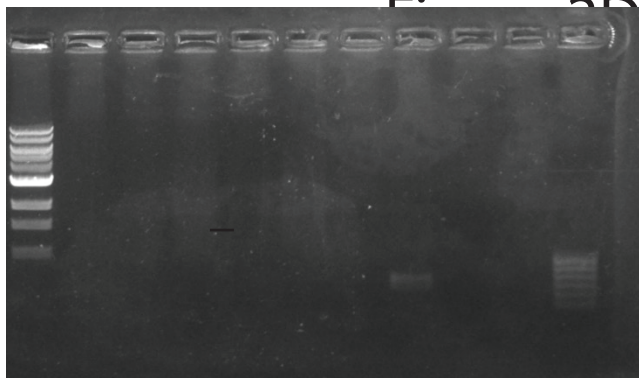

Figure 3F

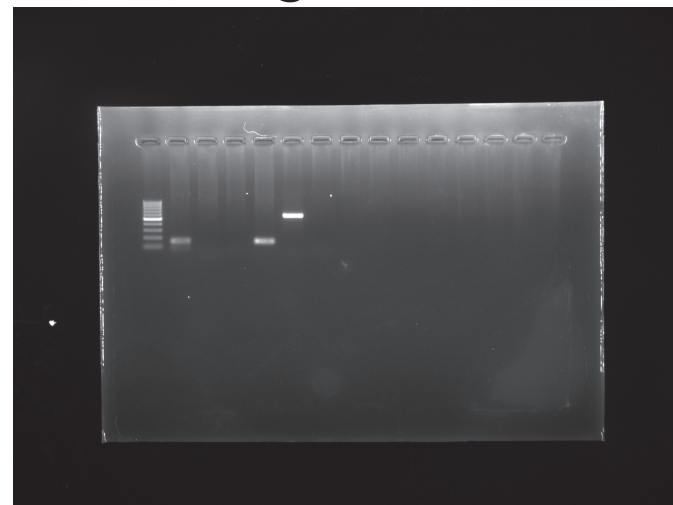

Supplementary Figure 1B

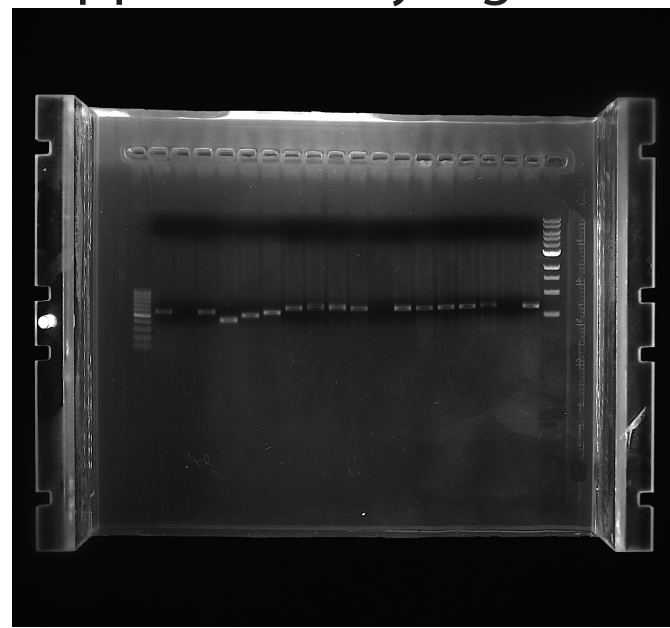

Supplement: Supplementary file 18 — Source Data [file 41467_2026_73891_MOESM18_ESM.pdf]
