# Supplementary material for: Asymmetric attrition and secondary chromosome destabilization after double-strand breaks in human embryonic development
Source: Nat Commun. 2026 Jun 3;17:7140. doi: 10.1038/s41467-026-73891-7 (PMC13396663; doi:10.1038/s41467-026-73891-7)
Supplement: Supplementary file 17 — Transparent Peer Review file [file 41467_2026_73891_MOESM17_ESM.pdf]

nature portfolio

Peer Review File

Asymmetric Attrition and Secondary Chromosome Destabilization after Double-Strand Breaks in Human Embryonic Development

Corresponding Author: Dr Dieter Egli

This file contains all reviewer reports in order by version, followed by all author rebuttals in order by version.

Attachments originally included by the reviewers as part of their assessment can be found at the end of this file.

Version 0:

Reviewer comments:

Reviewer #1

(Remarks to the Author)
Review
The article describes the consequences of CRISPR/Cas9 targeted editing in human embryos with respect to chromosomal stability. The authors demonstrate that targeted editing results in a significant increase in chromosomal instability in the edited chromosomes. This observation is important and will guide any future editing experiments in human embryos and set a standard to evaluate chromosomal losses in any CRISPR/Cas9 editing experiments. In addition, the analyses of the chromosomal changes occurring, the mechanisms leading to chromosomal loss and rearrangement are being deduced from the observed genotype profiles. Overall, this manuscript provides novel insights important for the fields of gene editing, genetics and IVF.

Major comments
- The statement/claim that the functional centromere can be mapped, seems preposterous. The data does not fully support the claims as other possible reasons for the observations can be deduced. The position of the guide is based on the reference genome. The pericentromeric regions are very variable amongs individuals. How sure are the authors that the pericentromeric region in the embryo genomes are similar to the reference genome? Structural variation in the region would change the outcome and lead to a different interpretation.
- Line346: Thus, noncovalent tethering rather than end joining of sister chromatids account for the majority of chromosomal changes. This extrapolation of the observation that no tandem duplications were observed is a leap: The mechanism which would lead to a tandem duplication following an intrachromosomal break would be different from a breakage-fusion bridge cycle as it would be a three step process: fusion and subsequent second cleavage and rejoining. Tandem repeats are probably generated by different mechanisms. In the discussion the potential for tethering is explained, which is interesting. However, I do not think the data provide evidenced discriminating either tethering or covalent fusion. Considering that this tethering is put forward in the title, either the data should become more convincing or should be tuned down. Also in figure 3 it is stated that the fusion is non-covalent. Also here, I think the data to distinguish between a covalent or non-covalent bridge formation is lacking.
- Figure 4 and the supplementary figures are lacking and have not been reviewed.

Minor comments
- Intro line 79 ‘Furthermore, microdeletions and microduplications near centromeres can give rise to disorders such as 16p11.2 microdeletion or 16p11.2 duplication syndrome which have been associated with an increased risk of intellectual disability and psychiatric disorders’: Mechanistically, those genomic disorders are the result of NAHR and do not contribute to the argument that pericentromeric regions are more frequently involved in rearrangements. Other, non-pericentric genomic disorders are even more frequent (e.g. 22q11).
- Line 74: I wonder even whether the whole paragraph on meiotic DSB is correct: What has been demonstrated is that the location of the recombination sites, the chiasmata, affects chromosome segregation. To extrapolate this to argue that meiotic DSBs cause chromosomal instability in a similar way as might occur by Crispr induced DSBs is one step too far. Maybe remove this paragraph.

- Line 86: guides were designed to unique sites on 16 and X. However, later it turns out they are not unique. Rewrite/reword.
- Line 117: End joining was tested by PCR. Data? How was this done?
- Line 171: We stained Cripsr injected embryos with Hoechst and found excluded cytoplasmic fragments. How about controls? What is the frequency in non-injected controls?
- Line 192: For centromere-containing chromosome segments, a role of bridge formation may play a role, analogous to what has been observed in cultured human cells. Maybe the authors should also refer to observations made in IVF embryos where the same mechanism has been deduced from the chromosomal segregation patterns observed (e.g. Voet et al.,Human mutation, 2011; Vanneste et al., Nat. Med., 2009). Similarly, paragraph on attrition: The authors write 'the telomeric segment seems to be protected by attrition and go on describing breakage fusion bridge cycles as the mechanism underlying centromeric attrition. The breakage-fusion could have occurred on both the telomeric and centromeric segment, but only the dicentric fragment is forced to break again, explaining the attrition.
- Line 235: Here it is stated that the chromosome removal can be enhanced by combining probes, whereas in the previous sentence the opposite is stated:' The difference in chromosomal 16 copy number changes per blastomere was not statistically significant whether one or three cut sites were used'. Maybe explicit/reword.
- The guide cuts also chromosome 17: what is the stability of chr 17?

Reviewer #2

(Remarks to the Author)
The authors investigated CRISPR-Cas9 nuclease impact on chromosome integrity and ploidy in human embryos when DSB in near the centromere.

Targeting pericentromeric region of Chr 16 and ChrX, they observed high rate of chromosomic abnormalities induced by CRISPR-Cas9-induced DSB. They deeply described genotoxicity at different scales: chromosomal scale, megabase scale, kilobase scale, and small Indels. This study is well designed, with appropriate tools, and very interesting.
They observed, just at the cut site, reciprocal gain and loss of acentric chromosome arms in daughter cells, but only loss of the centric chromosome arm. The changes are the result of DNA-repair processes harnessed by genome-editing tools. This study confirms the significant ON-target genotoxicity of DSB in human embryos, precisely quantified it, and provides hypotheses of the mechanisms involved in these unwanted effects. Centric losses and gains seem to be specific to pericentromeric targeting because they were absent in mid-arm targeting.They hypothesized that DNA Double Strand Breaks cause chromosome loss through sister chromatid tethering in human embryos. The newest part of the article demonstrates that CRISPRCas9 genotoxicity can be hijack to precisely define/map active centromere region in embryo, or to correct aneuploidy, by a single DSB, as tested here for trisomic Chr16 embryo. They also observed asymmetric attrition at the cut site, with large deletion at the centromeric side and propose an original mechanism for deletion between the cut site and the centromere.

The work is original and very significant for the field. Quality of data is good, with appropriate technique, analyses and interpretation. There provided strong evidence of chromosome fragment loss, asymmetric attrition targeting pericentromeric loci by CRISPR-CAs9 nuclease in human embryos.

However, some questions should be raised to improve its quality.

1/ To explain the high rate of chromosomal fragment losses, they observed excluded cytoplasmic fragments containing DNA. Micronucleation rate for Chr 6 and ChrX will reinforced the data.
2/ They affirm that one single DSB can induce high level of ON-target genotoxicity. It is mandatory to check that chromosome instability is not due to OFF-target secondary cleavage in Chr16 and X. By using HIFI Cas9 for example.
3/ In the paper, "sister chromatid tethering" is not directly shown. Title is for the moment speculative to explain asymmetric chromosome losses: bridge presence have to be shown to conserve this title. Alternatively, a more appropriate title is needed.
4/ Part on figure 4 is less original and does not correspond to title article. To transfer in supplementary data. I suggest to change this original full paper in short communication. Alternatively, sister chromatid tethering with bridge evidence, or MRE11 implication will complete the study for a full paper.

In the discussion:
Major ON-target genotoxicity papers in non-embryos are lacking in the discussion: Kosicki M et al Nat Biotech 2018; Cullot et al Nat com 2019; Shin HY Nat com 2017;Weisheit I Cell rep 2020; Owens DDG NAR 2019…. For example.
Ethic about human embryo editing have to be balanced in particular when alternative, as human embryo sorting, is possible.

Reviewer #3

(Remarks to the Author)
DNA Double Strand Breaks cause chromosome loss through sister chromatid tethering in human embryos

Turocy et al., 2022

This study tests the effects of CRISPR/Cas9 mediated genome editing on genome stability in human embryos. Given the potential of CRISPR/Cas9 mediated genome editing in treating inheritable genetic disorders it is important to evaluate its genomic effects in human embryos. This study reveals concerning results where 1/3 of the CRISPR/Cas9 targeted chromosomes in human embryos show either whole, segmented, or fragmented chromosome loss. The manuscript suggests that broken sister chromatids are covalently linked, leading to dicentric chromosomes or protection and tethering of acentric fragments. However, the results are very hard to follow and some of the claims are not entirely proven.

While the results of this study are important for the broad audience, the manuscript should be revised as many of the figures and accompanying text are not clear. I recommend major revision prior to reconsideration.

Major points:
1. Line 130: how did the author reach a combined 260 targeted chromosomes? This would imply that each embryo was treated with several gRNAs targeting different chromosome. A better approach in my view is to limit the treatment to one gRNA/embryo because this would better mimic a potential future CRISPR/Cas9 mediated treatment to either induce loss of a specific trisomy or to treat/prevent a specific inheritable genetic disorder.
2. Line 151: where are the 22 chromosome gains depicted in Fig. 1D? are they a part of the Indels?
3. Line 164: could the “cellular fragment” be a micronucleus?
4. Line 165: the authors suggest that such events can arise from sister chromatids fusion, resulting in dicentric chromosomes, bridge formation and breakage during mitosis. This is further suggested along the manuscript as a major mechanism inducing genome instability following CRISPR/Cas9 mediated genome editing. To strengthen this idea and prove this hypothesis, the author should repeat the experiment and follow the embryo using live cell imaging to detect the type of chromosome segregation aberrations. They should also perform IF staining with FISH probes to detect dicentric chromosomes, or break-fusion-bridge instances.
5. Line 172: “… cytoplasmic fragments containing DNA”. Do these have a nuclear membrane? Or a disrupted membrane? Are these micronuclei?
6. Fig. 1k – results do not seem to be statistically significant, as there are 5 breaks that are Cas9 induced and 8 spontaneous breaks. Please explain.
7. Line 192: “a role of bridge formation may play a role” – again live cell imaging would strengthen this argument.
8. Pages 11 – 13 related to figure 3 are very hard to follow. I’m not sure that the reference to certain figure panels is correct. I suggest re-writing this section. gRNAs should be numbered (i.e., simple nomenclature 1, 2, 3 etc together with a cytogenetic map that explains their location. The current nomenclature of the gRNAs makes it very hard to follow Figure 3 and relevant text.
9. The authors conclude in line 259 that “the annotated chr16 is not functional in human embryos”. In fact, the annotation of human centromeres in hg38 is not that clear/precise. Human centromeres in the human genome assembly (hg38) are represented by models that reflect the observed variation in human α-satellite repeat sequences but do not distinguish between centromeric and pericentromeric sequences, and arbitrarily assign the order of repeats in each centromeric array (Miga et al., Genome Research 2014). Those models are linear description of the variation within each centromere, but the borders between centromeres and pericentromeres are not defined. The authors might have cut in the middle of the centromere and not at the pericentromeric region. The first description of the accurate sequences of human centromeres was recently released as part of the T2T (telomere to telomere) genome assembly that uncovered the assembly of repetitive regions of the genome including the centromere (Nurk et al., Science 2022; Altemose et al., Science 2022). The authors should reevaluate the position of the guide RNA using this new genome assembly. It could be that the 0.7Mb of -satellite DNA is simply the active part of the centromere, where CENP-A binds, while the other repetitive -satellite DNA is the “dead” HOR that is not the active centromere. The authors should reanalyze their gRNA position based on the T2T genome assembly before making any conclusions.
10. Line 271 – “… acts as the functional centromere in human embryo..” and probably also in adult and somatic cells. The chance that a centromere will move places from the embryo later in development are very slim.
11. Line 284 – the description of the gRNA used here is so complicated! Please change the nomenclature so the reader can follow more easily. Are you referring here to gRNA 35126545?
12. Fig. 3i – please mark centromere location in the figure to be able to follow the text. Also mark where is the break. Figure is unclear.
13. If one cuts within a centromere that is highly repetitive, you could expect different secondary structures arising because of the repeats, trying to use homologous sequences for the repair but the alignment could be to a different repeat. That could result in deletions and duplications.
14. Line 301: what is the number of the cut site or gRNA used here?
15. Page 14, lines 301 – 311: very confusing, both text and relevant figures. Very hard to follow and understand. Needs further work to clarify.
16. Line 314: could it also be failure to repair because the cut site was in repetitive region within the centromere?
17. Line 317: “… the resulting dicentric chromosomes would break between the two centromeres…” this is an interesting hypothesis that should be further investigated using live cell imaging.
18. Line 333: “breakage between the cut site and the centromere would result in reciprocal and variably sized tandem duplications and deletions”. Why would the author expect that? Please explain. Why wouldn’t the authors expect to see just a simple loss? Please add schematics to be able to follow
19. Line 336: “… a total of 17 chr X targets”. How did you reach 17 Xs? Are some cells females and some males?
20. Line 345: “no tandem duplications were found in any sample”. This contrasts with the authors expectations in line 333. Please explain.

21. What does the author mean by “covalent linkage” or “noncovalent tethering”. Please add clear schematics.
22. Lines 346 – 352: the conclusion is not clear. Based on the next paragraph both options (noncovalent tethering and end joining of sister chromatids) occur at the experiment described in this study
23. In general, better schematics and explanations of figures within the figures themselves are needed across all the manuscript.
24. Fig. S1 – before showing examples of aberrations it would be great to see examples of a wt diploid cells. How does the baf analysis looks for a normal karyotype? And the SNP array? With a wt reference the reader can understand better all the other examples and data across the manuscript.

Minor points:
1. Line 58: “(reviewed in Turocy et al” – missing end parenthesis
2. Alpha – should be replaced with   along the manuscript
3. Line 265 – did the authors mean Fig. 3E?
4. Line 286 refers to Fig. 3g. I’m not sure if I downloaded the correct figure but I do not see 3g. There are 2 panels that are missing their annotations.
5. Line 292: “… apparent in 11/12 centromeric…” sentence seems unfinished
6. Line 295: refers to fig 3h. Annotation for Fig. 3H is missing in Fig. 3.
7. Line 317: no “K” annotation within Fig. 3.
8. Line 438 refers to Fig. 3J. There is no 3J in Fig. 3.
9. Fig. 3E: add “p arm” and “q arm” to the schematics. Color of hg19 annotated centromere is not obvious and needs to be changed.
10. Schematics at the bottom of Fig. 3 is not clear and is also missing a letter. Is this 3K? where is the dicentric chromosome and bridge formation in this model? What is the frequency of indels vs. noncovalent linkage/end protection?
11. noncovalent linkage/end protection can simply be called as fusion of sister chromatids at the cut site, to make it more understandable across the paper, together with schematics to show that.
12. Fig. 3i – what are the two lines at the bottom “embryo” and “gDNA control”
13. Fig. and Fig. legends: some have capital letters and some small letters across the paper. For example, Fig.S1 has S1a-g, but Fig. S4 has A-C panels.
14. Fig. S3 – I couldn’t find any B in the fig legends or in the manuscript. This Fig needs to be clarified more in the manuscript and in the legends too.

Version 1:

Reviewer comments:

Reviewer #2

(Remarks to the Author)
The authors present a revised version of their study describing the consequences of double-strand breaks (DSBs) in human embryos and their impact on chromosomal instability. They demonstrate that pericentromeric DSBs can induce heterogeneous chromosomal outcomes, including telomeric arm deletions or duplications, loss of centric chromosome arms, and interstitial centromeric deletions. They also propose a mechanism to explain the asymmetric genotypic profiles observed. Overall, this manuscript provides novel and important insights into the origin of asymmetric aneuploidy in embryos and highlights the potential risks associated with the use of CRISPR–Cas9 nucleases in human embryos. This is an original and significant contribution to the field.

Compared with the previous version, substantial revisions have been made, and the manuscript has been markedly improved. The experimental data now adequately support the authors’ conclusions.

• The title is now better aligned with the experimental evidence and accurately reflects the results.
• The proposed mechanism of non-covalent tethering (now referred to as “persistence of unrepaired DSBs”) between sister chromatids is more thoroughly investigated and is now convincing.
• Numerous additional experiments have been included. Live-cell imaging, PCR, and FISH analyses were performed to test and refine the proposed mechanistic hypothesis.
• The reference list has been expanded. However, references addressing spontaneous aneuploidy in human induced pluripotent stem cells (hiPSCs) during culture should be added.
• The original manuscript was difficult to follow. The revised text, together with the schematic in Figure 7, is now much clearer.

This revised version of the manuscript is suitable for publication.
However, I have several minor comments that could further improve the readability of the manuscript and the clarity of the figures:

• Figure 1G: To facilitate understanding, please define “CCF” in the figure legend.
• Figure 2A: Please indicate the DSB on chromosome X.
• Figure 2C: Please indicate the number of cells analyzed to clarify the correspondence with the 2D representation.
• Figure 4A: Please specify which chromosome(s) are targeted.
• Figure 4G, I, K, M: Please specify which chromosome is analyzed.
• Figure 5:
5A: Please specify hESCs in the experimental scheme.
5C: Please clarify what is meant by “distance between red/green probes.”
5D: Please clarify that “linked SNP” corresponds to the non-targeted allele, and that EYSfs corresponds to the targeted allele.
5E: Please clarify what is meant by “distance between …”.
• Figure S1A/B/C: Data from different loci are combined. Please specify the targeted loci directly in the figure.
• Figure S2: This figure is not sufficiently described or explained in the text (line 135). Please guide the reader through all panels.

Other minor comments
• Line 118: The term “chromosomal error” is unclear. Please clarify.
• Line 152: Embryos without Cas9 represent an appropriate control to assess spontaneous breakage events.
• Line 252: Please specify which PCR results are being referred to (1–2 or 1–3).
• Lines 219–223: The statement “Asymmetry … chromatids” represents a hypothesis rather than a conclusion and should be moved to a more appropriate section of the manuscript.
• Using consistent terminology for chromosome fragments may improve readability. Consider choosing between “centromeric-containing/centric” and “telomeric/acentric” throughout the manuscript.
• Do the authors observe chromothripsis in micronuclei? If so, please discuss this point.

Reviewer #3

(Remarks to the Author)
Asymmetric Chromosomal Consequences at Double Strand Breaks in Human Embryonic Development

Turocy et al., 2026

DNA repair mechanisms in the human embryo remain poorly understood and DSBs can have adverse consequences, including chromosome loss. In this revised study, the authors test the effects of CRISPR/Cas9 mediated genome editing on genome stability in human embryos. The study show concerning results where 1/3 of the CRISPR/Cas9 targeted chromosomes in human embryos show either whole, segmented, or fragmented chromosome loss. Moreover, the chromosomal alterations were asymmetric relative to an induced DSB: acentric arms showed complementary gains and losses, and overall were mostly lost, while centric arms were biased toward losses.
Using a variety of methods, the study shows that broken chromosomal fragments that contain centromeres are lagging during mitosis, and that their segregation is often abnormal and can result in exclusion of the chromosome fragment from the cell, secondary breakage and attrition of genomic material between the cut site and the centromere. In contrast, acentric chromosomal fragments do not show an additional break or attrition at the CRISPR/Cas9 cut site. These results highlight the risks of chromosomal-scale changes in CRISPR/Cas9 genome editing and reveal how a single DSB destabilizes am entire chromosome in the human embryo. Lastly, the author propose that these asymmetric differences are arising because the original DSB was not repaired. Given the potential of CRISPR/Cas9 mediated genome editing in treating inheritable genetic disorders it is important to evaluate its genomic effects in human embryos. This study reveals that clinical use of CRISPR/cas9 genome editing in the embryo is too genotoxic. They also show that DSBs occur spontaneously in the human embryos and that the consequence of induced DSBs are similar to spontaneous DSBs.

I appreciate the authors taking the time to significantly improve and substantiate their data using different approaches, including live-cell imaging that I proposed. I also think that they have answered most of my comments and have improved the work significantly in the revised version.
While the results of this study are important for the broad audience, and the experiments are mostly sound, I’m still left with several major issues that should be addressed.

The main issue in my view is novelty. I’m trying to understand what is the main take-home message and is it new? For example, line 335 says: ”thus, sister chromatids remain in proximity, while DSB ends are prone to separate from each other”. I’m not sure what is novel in these results. It is widely known that an acentric fragment will get lost, and if a fragment contains a centromere, the centromere will mediate segregation and separation. Can the author expand on the novelty of their results across the manuscript compared to what is known in the field as consequences of DSBs in somatic cells and in embryos. For example, do DSBs tend to be repaired more efficiently in somatic cells? Can they compare between the two and demonstrate inefficient repair in embryos?

In addition, while improved, figure legends and text need further review and refinement.

Major points:

1. Novelty –What is the main take-home message and is it new? Can the author expand on the novelty of their results across the manuscript compared to what is known in the field as consequences of DSBs in somatic cells and in embryos.
2. This work, as is, shows the consequences of DSBs in embryos, and the lack of their repair in many instances. However, there is no attempt to explain that mechanistically. What causes embryos to lack in their repair capacity.
3. Fig. 2D – Can a PCR amplify a pericentromeric repetitive region? A positive control from a wt cell is missing. Also, what is "other"? no explanation is given within the fig legend.
4. Figure legends across all figures should be improved to include all necessary details. For example, the legend of Fig. 2D is not sufficiently detailed to understand the data shown. The meaning of "other" is not explained. Fig legends should be detailed and include number of repetitions, and explanations of samples names, etc.
5. Line 209: can the attrition be attributed to technical difficulties in sequencing/SNP analysis of that region? Can some SNPs be harder to amplify? If yes, this should be included in the explanation of study limitations.
6. Line 249: "sister chromatids appeared as separate visual signals in mitosis … suggesting they were not a covalently linked entity". – why would the authors expect sister chromatids to be covalently linked? Sister chromatids are held together at the centromere by cohesin. The broken arms do not have a centromere and are not supposed to be held together. I'm not sure I understand the novelty of these results.
7. Additional schematics panels will improve the understanding of current Fig. 4E and 4G.
8. Fig. 5B is not annotated. What does each microscope image represent?? What is control, what is Cas9 treated, colors of probes etc. Basic Figure preparation and revision is absent.
9. The authors propose that if the DSB is not repaired, than it will likely progress into linkage of the centric fragments resulting in a lagging chromosome with two centromeres that is different than a dicentric chromosome that arise through break-fusion-bridge mechanism. Indeed, the break-fusion-bridge mechanism should lead to duplications, which are not observed in the results presented here, reducing the chances that what we are seeing are indeed dicentric chromosomes. Perhaps long-read sequencing could help resolve the structure of the derivative chromosome observed here.
10. The author should clarify across the text what is the difference between fusion of the centric fragments that lead to dicentric chromosome compared to linkage of the sister chromatids that also results in a dicentric structure as their model propose. This is central to their work and their model but is not easy to understand and discriminate between the two.

Minor points:
1. Line 118 "… due to a chromosomal error on the other chromosome". I suggest rephrasing 'chromosomal error' to arm loss or segmental loss.
2. Line 153: "breakpoints .. are located at WWOX, an olfactory receptor gene cluster, WDR25, DPP10, RPTOR…". Are these known as fragile sites? if yes, it would be worth to mention it in the text.
3. Fig. S4 – no individual panels (A, B, C…) make it harder to follow the text. Also, addition of chromosomal schematics can be helpful in interpreting the sequencing data.
4. Fig. S5 - addition of chromosomal schematics can be helpful in interpreting the sequencing data.
5. Legend of Fig. 4D is not complete and is unclear. Many details are missing.
6. Lines 261 – 263:" not all expected genomic material…" sentence is not clear. Please rephrase.
7. Paragraph related to Fig. 4F-M (p. 13-14) is unclear. Please rephrase.
8. Line 289: "two chromosomes were again apparent" – please change to DNA entities or fragments as one cannot know from microscopy if these are full chromosomes or fragments.
9. Line 292: "… can get expelled from dividing cells, resulting in bias of spontaneous … towards chromosome loss" – micronuclei do not have to be expelled. The DNA they contain can also be fragmented and lost.
10. It is not clear to me what can be learned from Fig. S6D. It is completely blurry.
11. Fig. 5D missing Y axis
12. Line 409: "the nature of this linkage may be end-joining of sister chromatids resulting in bicentric chromosomes". The correct term is 'dicentric'. In addition, lagging chromosomes can be a result of erroneous microtubule attachment (merotelic attachment). They do not necessarily represent dicentric chromosomes.

Reviewer #4

(Remarks to the Author)
The article addresses the consequences of CRISPR/Cas9 editing in human embryos and the subsequent small and large scale errors produced. The induced chromosomal instability via DSB formation provides an excellent view into the limitations of DNA repair within the human embryo. The authors have done a thorough job addressing the comments from Reviewer #1, however, there are a few points within the revised draft that I would like to see resolved (see below).

Major Comments:
Reviewer #1 comment: The statement/claim that the functional centromere can be mapped, seems preposterous. The data does not fully support the claims as other possible reasons for the observations can be deduced. The position of the guide is based on the reference genome. The pericentromeric regions are very variable amongst individuals. How sure are the authors that the pericentromeric region in the embryo genomes are similar to the reference genome? Structural variation in the region would change the outcome and lead to a different interpretation.

Author response: this section was extensively revised and is now a minor point. We now have access to a T2T genome assembly, and the gRNA which maps to the p arm on hg19 and hg38, maps to the q arm on T2T. Our data suggested that the gRNA targeted the q arm, as the q arm was primarily affected by segmental aneuploidies. We write: "One gRNA site was

located on the p arm of chromosome 16 according to the hg38 genome assembly (Chr16:35126545 at 16p11.1), and to the q arm according to the T2T genome assembly 16q11.2 at chr16:39002348. The q arm was primarily affected by Cas9 cleavage at that site, congruent with the location relative to the centromere according to T2T (Fig. S4). At the time of gRNA design, the T2T assembly was not yet available – frequent q arm changes pointed to an error in the annotation of hg38, and are validating T2T."

Reviewer #4 comment:
I have some concerns about this change. Conducting a BLAST search of the gRNA sequence provided in Table S1 against the T2T-CHM13v2.0 genome using the NCBI Genome Data Viewer, the browser places the sequence mostly within the centromeric region but is still located on the p-arm. However, when using the UCSC Genome Browser, the browser places the sequence in 16q11.2. My look into this was very quick (and likely simple to explain), but I would appreciate a slightly deeper description of this change.

Additionally, if we assume that the Cas9 cut-site has switched from the p-arm to the q-arm I am confused by some conclusions made with the SNP maps. When looking at chromosome maps in Fig. S4 (annotation from hg19 and centromere placement from T2T), it is stated that there has been a "loss of genomic material between the Cas9 cut site and the centromere…" (lines 196-197). However, with the hg19 and T2T combination this conclusion is based on a cluster of deleted SNPs that would (if located on the q-arm) theoretically fall within the highly repetitive proximal region of chromosome 16q which has been a clear gap in all historical assemblies. I am questioning whether combining the alignments like this is doable or if it is leading to skewed conclusions. I understand that it would be a particularly large undertaking, but for any conclusions based on the Chr 16p arm* gRNA the entire SNP map should be converted to the T2T alignment.

Minor comments:
Reviewer #1 comment: Intro line 79 'Furthermore, microdeletions and microduplications near centromeres can give rise to disorders such as 16p11.2 microdeletion or 16p11.2 duplication syndrome which have been associated with an increased risk of intellectual disability and psychiatric disorders': Mechanistically, those genomic disorders are the result of NAHR and do not contribute to the argument that pericentromeric regions are more frequently involved in rearrangements. Other, non-pericentric genomic disorders are even more frequent (e.g. 22q11).

Author response: We now include reference to deletion syndromes on chromosomal arms, including 22q11. The reason why we specifically mention 16p11.2, is because we have guide RNAs targeting those areas.

Reviewer #4 comment: The authors state that deletions and duplication at 16p11.2 are referenced due to the gRNAs used in this study and not as a pericentromeric argument. However, the 22q11.2 deletions are then listed as an example that they can "also occur on chromosomal arms…". I find this wording confusing because the breakpoints for 16p11.2 and 22q11.2 are nearly the same distance from their respective centromeres and are not necessarily different categories of breakpoints.

Additional Reviewer #4 comments:
The authors stated that between the initial submission and re-submission there have been changes in the scope and data of the manuscript, as well as in the personnel involved in the project. Unfortunately, this is visible in the manuscript. Throughout the text there are several points where values do not add up and/or do not match figures, incorrect figures are referenced, or in some cases reference figure panels that are no longer included. I have included a list of some edits below that need to be addressed before publication. It is incredibly difficult to maintain an overview on manuscripts of this complexity and prolonged duration, therefore I would suggest that the authors find a fresh set of eyes to comb through the text and perhaps rework the supplemental tables for clarity, allowing the reader to more easily validate findings.

Table S2:
• Cells J48 through J68 are showing incremental values and not the original gRNA value (ChrXp: 58089779)

Table S3:
• The numbers for this table do not match the text.
- In text – 10 LOH, 18 no amplification, 8 complex (lines 90-95)
- In table – 9 LOH, 21 no amplification, 6 complex
• Sequenced chromosome containing cellular fragments
- The "chromosome containing cellular fragments" are labelled in column D, however, these fragments do not correspond to the "sample type" reported in column B. 4 of the 5 "cellular fragments" are listed as blastomeres (C2-3_16qXq, D1-4_16qXq, G2_1_Xp, and G2_5_Xp).
- In text – 5 chromosome containing cellular fragments/cells (line 95-97)
- In text – 5 embryos with fragments (matches line 145)
- In table – 6 chromosome containing cellular fragments/cells from 5 embryos

Figure 1:
• (D) Did you look at your samples the other way? I.e. was loss of chromosomes X more likely to occur when embryos were injected with gRNA targeting chromosome X?
• (G):
- Again the "fragments" that are denoted here need to be somehow highlighted in Table S4.

- It took a while to determine which embryos are referenced here, but it looks like there is a fragment that has not been reported. Embryo I1_F_XDMD_Chr16pprox_82.CEL is listed as also containing a breakpoint on chromosome 8 in WWP1 (Table S4, Line 102). This fragment is not denoted in Fig. 1G, nor in the text describing the breakpoints (lines 152-158). When double checking the breakpoint (chr8:87386539-87404553) in T2T it doesn't seem to correspond to WWP1 but to a series of repeat sequences, which is also of interest due to increased potential for repair errors.

Minor text changes:
• Line 86: delete extra "in"
• Lines 90-95: double check numbers
• Line 95: double check numbers (6 chromosome containing fragments from 5 embryos)
• Line 102: I don't know where 260 comes from. By counting the number of gRNA in each of the 113 embryo cells (Table S2) I only count 244 (gRNA 1: 113, gRNA 2: 77, gRNA 3: 54). Am I calculating this incorrectly?
• Line 113: The text states that three Indels were larger than 100bp, but Fig. S1A only shows two events larger than 100bp (-358 mmej and -132 nhej).
• Line 121: update denominator to 260
• Line 144: Fig. 1E should be Fig. 1F.
• Line 147: Fig. 1F should be Fig. 1G.
• Line 221-223: Typo in sentence grammar.
• Line 251-252: There is no Figure 3D.
• Please provide more consistent labeling of stages in figure 4.
• Line 308: check punctuation
• Line 353: missing word
• Lines 380-382: I understand that the authors are attempting to broaden the context of these findings, however regarding human embryos as a model for other biological systems seems inappropriate and incorrect.
• Lines 388-391: Here you state that from your observations it is likely a 'lack' of repair rather than abnormal repair, but also shows evidence of 'inefficient' repair. With this word choice these two statements seem counter to each other. I would consider 'inefficient' repair to be present but not functioning properly and not a 'lack'. Consider re-wording.
• Line 392: Consider adding that these errors occur "in mouse embryos and in somatic cells" for clarity.

Version 2:

Reviewer comments:

Reviewer #2

(Remarks to the Author)
The authors have addressed my major comments, and the manuscript is now clearer and easier to read. Overall, I believe it could be suitable for publication, provided that the following remaining points are adequately addressed.

Major point:
The title remains somewhat vague and does not fully highlight the originality of the study. In particular, the key novel aspects appear to be asymmetric attrition and secondary chromosome destabilization. I would therefore encourage the authors to revise the title to better reflect these contributions. For example: "Asymmetric Attrition and Secondary Chromosome Destabilization after Double-Strand Breaks in Human Embryonic Development."

Line 78:
Single versus multiple double-strand breaks (DSBs) can trigger distinct repair mechanisms. However, the main conclusion of the manuscript focuses on the consequences of an unrepaired DSB (see Fig. 7). In this context, the experiment using a non-specific gRNA inducing multiple DSBs on chromosome 16 seems less appropriate and may not be fully consistent with the central message of the paper. I would suggest removing this experiment from both the main text and the figures, or alternatively, providing a stronger justification for its inclusion.

Minor points:

Line 102: "16s" should be corrected.
Lines 138–139: The sentence is unclear and would benefit from rephrasing.
Figure 1C: There appears to be a loss of the non-targeted arm of chromosome 16. Could this reflect an off-target effect or an issue with the gRNA/DSB location? Please clarify and provide an explanation.

Reviewer #3

(Remarks to the Author)
Asymmetric Chromosomal Consequences at Double Strand Breaks in Human
Embryonic Development

Turocy et al., 2026, revised version

I have reviewed the revised manuscript by Turocy, Jerabek et al. and the authors' responses to my previous comments. I am satisfied that the majority of my concerns have been addressed, and I appreciate the significant effort that has gone into this

revision.
The authors have substantially improved the manuscript. The addition of live-cell imaging, FISH analysis, and the revised mechanistic model in Figure 7 have considerably strengthened the study. The figure legends are clearer, the terminology is more consistent, and the logic connecting the experimental observations to the proposed model is now easier to follow.

I am recommending acceptance of this manuscript, with one remaining comment for the authors to consider:

Regarding repair efficiency in embryos versus somatic cells: I had previously asked whether the authors could provide a direct experimental comparison of DSB repair efficiency between human embryos and somatic cells, as this would substantiate their claim that inefficient repair is the primary driver of the chromosomal consequences observed. The authors have addressed this point by citing relevant literature (refs. 25 and 28) showing that similar chromosomal outcomes occur in somatic cells when checkpoint kinases are inhibited, and that repair kinetics are slow in neurons and pluripotent stem cells. While I accept that a dedicated experimental comparison is beyond the scope of this study, I would encourage the authors to be explicit in the text that the comparison to somatic cell repair is based on cited evidence rather than data generated in this study. A brief clarifying sentence in the Discussion to this effect would help readers calibrate the strength of this specific claim.

Overall, this is an important and timely study that provides novel mechanistic insight into the chromosomal consequences of double-strand breaks in human embryos, with clear implications for the safety of heritable genome editing. I support publication in Nature Communications.

Reviewer #4

(Remarks to the Author)
I thank the authors for addressing the discrepancies in numbers and for including additional text for clarity. There are a few minor final points that I would like to see addressed prior to publication.

Changes to figures:
• Figure 1D – change y-axis to include the whole bar
• Figure 2C cell 4 trace screenshot includes a text pop-up about the horizontal axis
• Many of the copy number traces throughout the manuscript have been cut off at the bottom and are not showing the x-axis (figure 1C, 2C, S2B, S2E, S3B, S3C, S4B, S5B, S6B)

Suggestion for text:
I stated earlier concerns about variation in the location of the chromosome 16 cut-site in relation to the centromere identified in different genome browsers. While I feel this point was not fully addressed, I appreciate the authors re-running their analysis for verification. While I initially found the variation in centromere location surprising, further searching unearthed previous evidence in cell lines for centromeric shifts between the hg38 and T2T alignments (DOI: https://doi.org/10.26508/lsa.202201807). I think it would be a nice addition to state that this centromeric change is not as unexpected as I believe many readers will think.

Open Access This Peer Review File is licensed under a Creative Commons Attribution 4.0 International License, which permits use, sharing, adaptation, distribution and reproduction in any medium or format, as long as you give appropriate credit to the original author(s) and the source, provide a link to the Creative Commons license, and indicate if changes were made.
In cases where reviewers are anonymous, credit should be given to 'Anonymous Referee' and the source.
The images or other third party material in this Peer Review File are included in the article’s Creative Commons license, unless indicated otherwise in a credit line to the material. If material is not included in the article’s Creative Commons license and your intended use is not permitted by statutory regulation or exceeds the permitted use, you will need to obtain permission directly from the copyright holder.
To view a copy of this license, visit https://creativecommons.org/licenses/by/4.0/

REVIEWER COMMENTS on NCOMMS-22-13624

We thank the reviewers for the detailed reading of the manuscript, which has led us to perform new experiments with additional insight into the mechanisms of chromosome loss after a DSB. The basic conclusion is that DSB repair in the human embryo is inefficient and that progression through the cell cycle with a persistent break impairs sister chromatid separation at mitosis, producing asymmetric attrition and breakage centromeric, but not telomeric to the break site. These Cas9 induced chromosomal events mirror spontaneous breakages at fragile site, which also destabilize an entire chromosome. We now show new live cell imaging data in Fig. 4, new data on separation of DNA ends using FISH in Fig. 5, and new data on spontaneous breakages in Fig. 6.

Reviewer #1 (Remarks to the Author):

Review
The article describes the consequences of CRISPR/Cas9 targeted editing in human embryos with respect to chromosomal stability. The authors demonstrate that targeted editing results in a significant increase in chromosomal instability in the edited chromosomes. This observation is important and will guide any future editing experiments in human embryos and set a standard to evaluate chromosomal losses in any CRISPR/Cas9 editing experiments. In addition, the analyses of the chromosomal changes occurring, the mechanisms leading to chromosomal loss and rearrangement are being deduced from the observed genotype profiles. Overall, this manuscript provides novel insights important for the fields of gene editing, genetics and IVF.

Thank you for the detailed comments on the manuscript. It took us longer than expected to address these important comments, both because of the scope of the changes, and because the addition of new data required an IRB review, as well as because of personnel changes. We appreciate the willingness to evaluate a revised manuscript addressing the points raised.

Major comments
- The statement/claim that the functional centromere can be mapped, seems preposterous. The data does not fully support the claims as other possible reasons for the observations can be deduced. The position of the guide is based on the reference genome. The pericentromeric regions are very variable amongs individuals. How sure are the authors that the pericentromeric region in the embryo genomes are similar to the reference genome? Structural variation in the region would change the outcome and lead to a different interpretation.

Response: this section was extensively revised and is now a minor point. We now have access to a T2T genome assembly, and the gRNA which maps to the p arm on hg19 and hg38, maps to the q arm on T2T. Our data suggested that the gRNA targeted the q arm, as the q arm was primarily affected by segmental aneuploidies. We write:
"One gRNA site was located on the p arm of chromosome 16 according to the hg38 genome assembly (Chr16:35126545 at 16p11.1), and to the q arm according to the T2T

genome assembly 16q11.2 at chr16:39002348. The q arm was primarily affected by Cas9 cleavage at that site, congruent with the location relative to the centromere according to T2T (Fig. S4). At the time of gRNA design, the T2T assembly was not yet available – frequent q arm changes pointed to an error in the annotation of hg38 [1], and are validating T2T."

- Line346: Thus, noncovalent tethering rather than end joining of sister chromatids account for the majority of chromosomal changes. This extrapolation of the observation that no tandem duplications were observed is a leap: The mechanism which would lead to a tandem duplication following an intrachromosomal break would be different from a breakage-fusion bridge cycle as it would be a three step process: fusion and subsequent second cleavage and rejoining. Tandem repeats are probably generated by different mechanisms. In the discussion the potential for tethering is explained, which is interesting. However, I do not think the data provide evidenced discriminating either tethering or covalent fusion. Considering that this tethering is put forward in the title, either the data should become more convincing or should be tuned down. Also in figure 3 it is stated that the fusion is non-covalent. Also here, I think the data to distinguish between a covalent or non-covalent
bridge formation is lacking.
- Figure 4 and the supplementary figures are lacking and have not been reviewed.

RESPONSE: We changed our title to: "Asymmetric Chromosomal Consequences at Double Strand Breaks in Human Embryonic Development"
The noncovalent mechanism for chromosome breakage is not the main conclusion of the article, but one aspect of the discussion section.
We now add the following new data in the manuscript, consistent with noncovalent mechanisms:

- **Secondary breakages, which occur distant from the break site at or near the centromere are reminiscent of 'breakage fusion bridge cycles'. But they differ in important ways:**
- **The strongest support for noncovalent linkage between sister chromatids is the asymmetric attrition centromeric and telomeric of the cut site, simultaneous WITH secondary breakage in the (peri-) centromeric region (Fig. 3B). Covalent fusion protects the break point position, noncovalent linkage does not. We add additional numbers of attrition coordinates and the difference is highly significant. (Fig.3A,B).**
- **Live cell imaging shows that acentric fragments can separate to two fragments during the following interphase, suggesting that the two sister chromatids remain separable and are thus not covalently linked (Fig. 4A).**
- **We add new FISH data using breakage probes, showing that sister chromatids remain in proximity, while the broken chromosome ends dissociate from each other, consistent with inefficient re-joining of DSB ends (Fig. 5).**
- **There are other, supportive data of noncovalent linkage, e.g. the lack of PCR products using primers to amplify inverted duplications at conserved break points.**

In the discussion, we propose a simple mechanism of that linkage: it is based on the persistence of a DSB into S-phase. "The presence of an unrepaired DSB in S-phase is detrimental: a replication fork meeting a DSB will collapse. Mechanisms to recover the fork cannot rely on an intact parent strand and are typically unproductive or even detrimental. The persistence of unreplicated DNA or of recombination intermediates into mitosis can impede chromosome separation (Fig. 6)…."

The persistence of unrepaired DSBs is the most parsimonious and unifying interpretation of essentially all observations of this study.

Minor comments
- Intro line 79 'Furthermore, microdeletions and microduplications near centromeres can give rise to disorders such as 16p11.2 microdeletion or 16p11.2 duplication syndrome which have been associated with an increased risk of intellectual disability and psychiatric disorders': Mechanistically, those genomic disorders are the result of NAHR and do not contribute to the argument that pericentromeric regions are more frequently involved in rearrangements. Other, non-pericentric genomic disorders are even more frequent (e.g. 22q11).

Response: We now include reference to deletion syndromes on chromosomal arms, including 22q11. The reason why we specifically mention 16p11.2, is because we have guide RNAs targeting those areas.

- Line 74: I wonder even whether the whole paragraph on meiotic DSB is correct: What has been demonstrated is that the location of the recombination sites, the chiasmata, affects chromosome segregation. To extrapolate this to argue that meiotic DSBs cause chromosomal instability in a similar way as might occur by Crispr induced DSBs is one step too far. Maybe remove this paragraph.

Response: indeed, this section was formulated inadequately, thank you for pointing it out. It was removed. We only comment on meiosis with regard to an embryo with an abnormal meiosis of chromosome 13 and 16 in supplementary Figure 2.

- Line 86: guides were designed to unique sites on 16 and X. However, later it turns out they are not unique. Rewrite/reword.

Response: thank you, the term unique was removed. One site is not unique on chromosome 16.

- Line 117: End joining was tested by PCR. Data? How was this done?

Response: We write in the results section: "InDels and large deletions were evaluated through PCR and Sanger sequencing."

- Line 171: We stained Cripsr injected embryos with Hoechst and found excluded cytoplasmic fragments. How about controls? What is the frequency in non-injected controls?
Response: our study shows both Cas9 induced fragments as well fragments with spontaneous chromosomal break points. There are more fragments with coordinates characteristic of spontaneous break points (Fig. 1G). While we do not show separate data on excluded fragments in uninjected controls, we cite the available literature which shows that human embryos and rhesus macaque embryos show frequent chromosome fragments [2,3]. The novelty of our data is in the provision of genomic break point coordinates of these fragments. We also cite a study on chromosomal fragility in uninjected controls showing frequent spontaneous chromosomal breakage [4].

- Line 192: For centromere-containing chromosome segments, a role of bridge formation may play a role, analogous to what has been observed in cultured human cells. Maybe the authors should also refer to observations made in IVF embryos where the same mechanism has been deduced from the chromosomal segregation patterns observed (e.g. Voet et al.,Human mutation, 2011; Vanneste et al., Nat. Med., 2009). Similarly, paragraph on attrition: The authors write 'the telomeric segment seems to be protected by attrition and go on describing breakage fusion bridge cycles as the mechanism underlying centromeric attrition. The breakage-fusion could have occurred on both the telomeric and centromeric segment, but only the dicentric fragment is forced to break again, explaining the attrition.

**Response: This is a key point of the manuscript which we strengthened through the addition of live cell imaging data, additional karyotypes (Fig. 4), and FISH analysis after Cas9 cleavage (Fig. 5), as well as allele-specific PCR at a Cas9 target (Fig. 5D). We concur with the reviewer: only centric to the DSB are there forces that will cause secondary breakage. We now cite the above mentioned study consistent with a breakage cycle [5].
The key conceptual advance of this study is that fusion through end joining of sister chromatids may not be required to impede segregation and force subsequent breakage and destabilization of a chromosome. Instead, the mere presence of an unrepaired DSB during S-phase would be sufficient. Previous studies did not consider this possibility as a basis for interpretation of the data. We do now know that DSBs can persist for extended time periods from this as well as other studies (e.g. [6]). This changes the interpretation of the data of earlier studies in basic ways. If a replication fork meets an unrepaired DSB, it will fail to progress and attempts to repair it using the sister chromatid are more likely to be detrimental than productive. In this study, the data are inconsistent with dicentric chromosomes fused through end joining for the following reasons:**

Covalent linkage should equally protect the DSB ends both centromeric and telomeric of the Cas9 break site. To relieve mechanical forces, a dicentric chromosome would only need to break once, e.g. near the centromere. There is no reason for an additional break at or near the original Cas9 break site, no more than at any other site between centromere and chromosome end. To achieve the products observed (Fig. 3B), a dicentric centromere would need to undergo two or three breakages, including near the original Cas9 site. The

knowledge of the position of the Cas9-induced DSB break allows for such evaluation, while previous studies of spontaneous breaks do not know the initial break point(s).

We write: "End joining of sister chromatid ends should equally protect the position of the ends centromeric and telomeric to the Cas9 cut site. Head-to-head fusion would result in covalently linked bicentric chromosomes that form bridges and break during mitosis. Breakage between the centromeres of dicentric chromosomes can result in inverted duplications, which manifest as complementary gains and losses after separation into daughter cells. Though we did observe the losses, we did not also observe complementary gains between the centromere and the cut site in sister cells. Instead of duplications, we observed disjointed fragments spanning from the attritted break site to the centromere. Attrition and secondary breakage near the centromere lead to the model shown in Fig. 7. Replication into an unrepaired DSB result in linkage between sister chromatids, leading to lagging chromosomes, breakage near the centromere and separation of linked sister chromatids, unprotecting the Cas9 break site on the centromeric, but not the telomeric side."

We cite both studies Voet et al. 2011 and Vanneste 2009 at several points throughout the manuscript and discuss them quite extensively, emphasizing congruence. For instance:

"While this and an earlier study[5] agree with regard to an active breakage cycle in the early human embryo, they differ with regard to the mechanism leading to the destabilization of the chromosome: covalent end joining versus noncovalent linkage."

"Though previous studies have seen chromosome breakage and frequent segmental copy number changes [5], the interpretation of the data as breakage fusion bridge cycles and ring chromosomes is based on the premise that DSB ends are in fact being repaired, rather than based on direct observation of circular or bicentric chromosomes. Instead, the data may also be interpreted as the presence and propagation of disjointed chromosome segments."

- Line 235: Here it is stated that the chromosome removal can be enhanced by combining probes, whereas in the previous sentence the opposite is stated:' The difference in chromosomal 16 copy number changes per blastomere was not statistically significant whether one or three cut sites were used'. Maybe explicit/reword.

Response: We removed this statement. We merely say that all sites incur frequent chromosome changes and that there may be differences in frequency but do elaborate on statistical comparison or mechanism of such potential differences. Citing from results section: "Though not all sites display equal frequency in causing segmental errors, they all showed the same pattern. While acentric segments showed both losses and gains, centric fragments were all infrequent losses."

- The guide cuts also chromosome 17: what is the stability of chr 17?

Response: As for chromosome 16, the off-target site on chromosome 17q incurs Cas9 induced breakage and frequent aneuploidy as indicated in Fig.2E., and supplemental Figure S3.

Reviewer #2 (Remarks to the Author):

The authors investigated CRISPR-Cas9 nuclease impact on chromosome integrity and ploidy in human embryos when DSB in near the centromere.

Targeting pericentromeric region of Chr 16 and ChrX, they observed high rate of chromosomic abnormalities induced by CRISPR-Cas9-induced DSB. They deeply described genotoxicity at different scales: chromosomal scale, megabase scale, kilobase scale, and small Indels. This study is well designed, with appropriate tools, and very interesting.
They observed, just at the cut site, reciprocal gain and loss of acentric chromosome arms in daughter cells, but only loss of the centric chromosome arm. The changes are the result of DNA-repair processes harnessed by genome-editing tools. This study confirms the significant ON-target genotoxicity of DSB in human embryos, precisely quantified it, and provides hypotheses of the mechanisms involved in these unwanted effects. Centric losses and gains seem to be specific to pericentromeric targeting because they were absent in mid-arm targeting. They hypothesized that DNA Double Strand Breaks cause chromosome loss through sister chromatid tethering in human embryos. The newest part of the article demonstrates that CRISPRCas9 genotoxicity can be hijack to precisely define/map active centromere region in embryo, or to correct aneuploidy, by a single DSB, as tested here for trisomic Chr16 embryo. They also observed asymmetric attrition at the cut site, with large deletion at the centromeric side and propose an original mechanism for deletion between the cut site and the centromere.

The work is original and very significant for the field. Quality of data is good, with appropriate technique, analyses and interpretation. There provided strong evidence of chromosome fragment loss, asymmetric attrition targeting pericentromeric loci by CRISPR-CAs9 nuclease in human embryos.

Response: Thank you very much for these encouraging comments. We further extended these studies with live cell imaging combined with blastomere karyotyping.

However, some questions should be raised to improve its quality.

1/ To explain the high rate of chromosomal fragment losses, they observed excluded cytoplasmic fragments containing DNA. Micronucleation rate for Chr 6 and ChrX will reinforced the data.
Is there micronucleation in FISH data which encompasses the targeted chromosome?
Response: This is an excellent point. We now provide live cell imaging data which show that micronucleation corresponds to Cas9 induced aneuploidies (Fig. 4B, C, Fig. 4D).

2/ They affirm that one single DSB can induce high level of ON-target genotoxicity. It is mandatory to check that chromosome instability is not due to OFF-target secondary cleavage in Chr16 and X. By using HIFI Cas9 for example.

Response: we do have off-target activity, such as on chromosome 17, and with another gRNA relevant to Fig. 4 on chromosomes 10 and 18. The number of off-target sites is small,

and typically on another chromosome than the primary target. For instance, we find show in Fig. S3 that cleavage on chromosome 17 resulted in a chromosome segment that was excluded from the cell.
In the context of this study, we consider it desirable, as it provides additional readouts. We emphasize in the introduction that the purpose here is not clinical use (Cas9 is too genotoxic. Clinical use in the context of reproduction is in our opinion entirely out of question).

3/ In the paper, "sister chromatid tethering" is not directly shown. Title is for the moment speculative to explain asymmetric chromosome losses: bridge presence have to be shown to conserve this title. Alternatively, a more appropriate title is needed.

Response: we changed the title to what we clearly document: "Asymmetric Chromosomal Consequences at Double Strand Breaks in Human Embryonic Development." The model underlying the asymmetric consequences is discussed in the discussion section: lack of end joining and persistence of an unrepaired DSB. Replication forks running into such breaks cannot progress, without opportunity to repair using an intact parental DNA strand. This is sufficient to produce the outcomes of secondary breakage at the centromere and attrition on the centromeric side of the DSB. See model in Fig. 7.

4/ Part on figure 4 is less original and does not correspond to title article. To transfer in supplementary data. I suggest to change this original full paper in short communication. Alternatively, sister chromatid tethering with bridge evidence, or MRE11 implication will complete the study for a full paper.

Response: former Figure 4 is now part of the supplemental material (Fig. S1). It does not play a major role in the narrative, providing supportive data for near complete Cas9 activity/cleavage. We provide new data on live cell imaging and FISH using breakage probes in pluripotent stem cells to elevate impact and to quality for a full paper.

In the discussion:
Major ON-target genotoxicity papers in non-embryos are lacking in the discussion: Kosicki M et al Nat Biotech 2018; Cullot et al Nat com 2019; Shin HY Nat com 2017;Weisheit I Cell rep 2020; Owens DDG NAR 2019.... For example.
Ethic about human embryo editing have to be balanced in particular when alternative, as human embryo sorting, is possible.

To address this comment, we emphasized the utility of Cas9 in the context of understanding DSB repair and aneuploidy in the early embryo, and de-emphasized the context to heritable editing in a clinical context. We also cite the above publications as follows. "Without even addressing considerations related to ethics and regulation, the genotoxicity of Cas9 alone makes it a highly implausible candidate for heritable editing. Genotoxicity of Cas9 is also well documented in other cell types, ranging from large

deletions to segmental chromosome truncations [7-12]"

Reviewer #3 (Remarks to the Author):

DNA Double Strand Breaks cause chromosome loss through sister chromatid tethering in human embryos

Turocy et al., 2022

This study tests the effects of CRISPR/Cas9 mediated genome editing on genome stability in human embryos. Given the potential of CRISPR/Cas9 mediated genome editing in treating inheritable genetic disorders it is important to evaluate its genomic effects in human embryos. This study reveals concerning results where 1/3 of the CRISPR/Cas9 targeted chromosomes in human embryos show either whole, segmented, or fragmented chromosome loss. The manuscript suggests that broken sister chromatids are covalently linked, leading to dicentric chromosomes or protection and tethering of acentric fragments. However, the results are very hard to follow and some of the claims are not entirely proven.

While the results of this study are important for the broad audience, the manuscript should be revised as many of the figures and accompanying text are not clear. I recommend major revision prior to reconsideration.

Response: we thank the reviewer for the particularly detailed comments, which encouraged us to design additional experiments and improve presentation. We strengthened the central element of chromosomal changes with detailed attention to asymmetric outcomes relative to the centromere and the cut site. We added new live cell imaging data to visualize Cas9-induced aneuploidies, followed by karyotyping, as well as FISH data in pluripotent stem cells.
Elements tangential to this narrative, including the size of InDels were relegated to supplemental materials. We are hoping this makes the manuscript also easier to follow.

Major points:
1. Line 130: how did the author reach a combined 260 targeted chromosomes? This would imply that each embryo was treated with several gRNAs targeting different chromosome. A better approach in my view is to limit the treatment to one gRNA/embryo because this would better mimic a potential future CRISPR/Cas9 mediated treatment to either induce loss of a specific trisomy or to treat/prevent a specific inheritable genetic disorder.

Response: the gRNAs target both alleles of the same chromosome, so the minimum is two chromosomes per embryo. For some embryos, we multiplexed, injecting RNPs for both chromosome 16 and chromosome X into the same embryo. This is now clarified in the results section and specified for each cell and embryo in Table S2.
We write: "Since blastomeres are diploid, each blastomere contains two homologous chromosome 16s and either one X chromosome in male embryos (XY) or two homologous X chromosomes in female embryos (XX). The gRNAs for chr16p and chrXq were not designed to be allele specific, thus targeting both homologous chromosomes, and were

injected either alone or in combination as indicated in Table S2. The combined number of autosomal and X chromosomal targets in female and male embryos is 260."

The main aim of this study is a mechanistic understanding; given the limiting material, a larger number of target sites allowed us to harness more data and improve our statistics (e.g. Fig.1D,E, Fig. 3A).
Notably, multiplexing is not a requirement to achieve chromosomal changes, a single gRNA is sufficient in this study, as well as in cited studies [13,14]. This suggests that we are looking at independent segregation errors.

2. Line 151: where are the 22 chromosome gains depicted in Fig. 1D? are they a part of the Indels?
Response: We now include the 22 gains in Fig. 1B. As they are dependent on the losses, they are not considered separate events.

3. Line 164: could the "cellular fragment" be a micronucleus?
Response: we now clarify the terminology: "Cellular fragments, defined as no nucleus in bright field microscopy and at least a factor of two smaller than nucleated blastomeres in the same embryo, were analyzed as well" Chromosomal segments in these fragments could be considered a micronucleus, albeit one that's excluded from a cell. Figure 1F provides an image of three such chromosome containing cellular fragments.

4. Line 165: the authors suggest that such events can arise from sister chromatids fusion, resulting in dicentric chromosomes, bridge formation and breakage during mitosis. This is further suggested along the manuscript as a major mechanism inducing genome instability following CRISPR/Cas9 mediated genome editing. To strengthen this idea and prove this hypothesis, the author should repeat the experiment and follow the embryo using live cell imaging to detect the type of chromosome segregation aberrations. They should also perform IF staining with FISH probes to detect dicentric chromosomes, or break-fusion-bridge instances.
Response: we now add live cell imaging data in a new Figure 4, Supplemental Figure 6 and supplemental movies 1-5. The main types of errors we find are chromosome fragments dissociated from the main spindle, as well as lagging chromosomes. Both can form micronuclei and can be lost from the embryo. Importantly, these findings lend support to a noncovalent linkage between sister chromatids, as one focus of a single acentric chromosome fragment can separate into two foci during the following interphase (Fig. 4A). We performed FISH using breakage probes in pluripotent stem cells. These data show that sister chromatids remain in proximity, while the appropriate repair partners move apart from each other after Cas9 cleavage (Fig. 5A-D). This finding is complemented with a depression of the targeted allele relative to a control (Fig. 5E).

5. Line 172: "… cytoplasmic fragments containing DNA". Do these have a nuclear membrane? Or a disrupted membrane? Are these micronuclei?
Response:
While we do not directly speak to this specific issue, we now show new data from live cell imaging experiments which show that even an acentric chromosome fragments are

packaged into a micronucleus with a clear rounded delineation towards the cytoplasm and with a diameter of ~5-7μm (Fig. 4B), or comparable in size to the nucleus of an ESC. Furthermore, these micronuclei replicate and amplify chromosome segments after unilateral segregation (Fig. 4F, G). We cite the relevant literature demonstrating unilateral segregation of micronuclei in mice [15].
Furthermore, genetic content of the micronuclei, which we determine, show amplification (e.g. Fig. 4G), which implies that these are nuclei with replication competence. Micronucleation is a common phenomenon in human embryos even spontaneously, and these micronuclei are still not that small, usually the size of the average somatic nucleus. Cells with micronucleation are genetically unstable (Fig. S6D, Supplemental Movie 5). Exploring both chromosome content and nuclear membrane integrity through immunostaining is challenging to do, as it involves fixing followed by DNA amplification from a single cell. This technique is not currently worked out technically.

6. Fig. 1k – results do not seem to be statistically significant, as there are 5 breaks that are Cas9 induced and 8 spontaneous breaks. Please explain.
Response: we are not seeking statistical significance in this data. The specificity break points now Fig. 1G and Table S4, is in the genomic coordinates, which match either fragile sites or Cas9 targets. The presence of both spontaneous and Cas9 induced broken chromosome segments point out that the exclusion as chromosome containing cellular fragments is a mechanism of chromosome loss from the embryo proper. We describe the results on page 9 and summarize as follows: "These results show that both spontaneous as well as Cas9 induced chromosome fragments can be excluded from the embryo proper in small cellular fragments."

7. Line 192: "a role of bridge formation may play a role" – again live cell imaging would strengthen this argument.
Response: we now add live cell imaging data. We found lagging chromosomes and chromosome fragments in mitosis. Though we do not observe bridges using SPY650 and live cell imaging, ultrafine bridges cannot be excluded.

8. Pages 11 – 13 related to figure 3 are very hard to follow. I'm not sure that the reference to certain figure panels is correct. I suggest re-writing this section. gRNAs should be numbered (i.e., simple nomenclature 1, 2, 3 etc together with a cytogenetic map that explains their location. The current nomenclature of the gRNAs makes it very hard to follow Figure 3 and relevant text.

Response: We restructured the paper in response to this comment, and with the inclusion of new data. All elements of Figure 3 with regard to the position of the centromere are no longer in the main figure because T2T corrected the issue; the gRNA is in reality located on the q arm, matching the results of frequent q arm changes. hg19 and hg38, which were available at the time, are incorrect in this regard.

The model of how DSBs produce segregation errors on either side of the break is no longer part of the results section but is now in a separate Figure referred to in the discussion section. Figure 3 is now focused entirely on attrition at the break site telomeric and centromeric of the cut site, and on secondary breakages due to spindle forces in mitosis.

9. The authors conclude in line 259 that "the annotated chr16 is not functional in human embryos". In fact, the annotation of human centromeres in hg38 is not that clear/precise. Human centromeres in the human genome assembly (hg38) are represented by models that reflect the observed variation in human α-satellite repeat sequences but do not distinguish between centromeric and pericentromeric sequences, and arbitrarily assign the order of repeats in each centromeric array (Miga et al., Genome Research 2014). Those models are linear description of the variation within each centromere, but the borders between centromeres and pericentromeres are not defined. The authors might have cut in the middle of the centromere and not at the pericentromeric region. The first description of the accurate sequences of human centromeres was recently released as part of the T2T (telomere to telomere) genome assembly that uncovered the assembly of repetitive regions of the genome including the centromere (Nurk et al., Science 2022; Altemose et al., Science 2022). The authors should reevaluate the position of the guide RNA using this new genome assembly. It could be that the 0.7Mb of α-satellite DNA is simply the active part of the centromere, where CENP-A binds, while the other repetitive α-satellite DNA is the "dead" HOR that is not the active centromere. The authors should reanalyze their gRNA position based on the T2T genome assembly before making any conclusions.

Response: the reviewer's reading of the data was indeed correct. The gRNA locates to the q arm on the T2T genome assembly. At the time of design and initial studies, T2T was not yet available. The pattern of chromosomal changes is now identical to other gRNAs: acentric losses and gains, and centric losses. We now de-emphasize the point of mapping centromere location relative to the gRNA based on chromosomal outcomes.

10. Line 271 – "… acts as the functional centromere in human embryo.." and probably also in adult and somatic cells. The chance that a centromere will move places from the embryo later in development are very slim.

Response: this is now a moot point because of T2T.

11. Line 284 – the description of the gRNA used here is so complicated! Please change the nomenclature so the reader can follow more easily. Are you referring here to gRNA 35126545?
Response: this is now addressed because of the correction provided by T2T. We also moved this material to supplemental, as the gRNA position is now correctly known on the q arm. In the supplemental, we explain that the SNP array analysis is performed based on hg19, and therefore some SNPs appear to be on the p arm, even as they are in reality on the q arm. This is relevant to Supplemental Figures 4 and 5, but does not impact the overall narrative.

12. Fig. 3i – please mark centromere location in the figure to be able to follow the text. Also mark where is the break. Figure is unclear.

Cas9 cut site and centromere are now marked.

13. If one cuts within a centromere that is highly repetitive, you could expect different secondary structures arising because of the repeats, trying to use homologous sequences for the repair but the alignment could be to a different repeat. That could result in deletions and duplications.
Response: we consider this unlikely to underly for the deletions we observed, for several reasons: i) directionality towards the centromere, ii) inefficient HDR in human embryos, iii) design of the gRNAs not directly in repetitive regions.
In response to this comment, we now cite literature showing that HDR in human embryos is inefficient, even in cis [16].

14. Line 301: what is the number of the cut site or gRNA used here?
We clarify that Figure 3A summarizes data from all gRNAs used. It includes nullisomic copy number transitions as these allow for precise mapping of copy number transitions. We write in the legend: "Chromosomal breaks due a unique Cas9 target site on chromosomes 16, 17, X and 6[14] were analyzed. Attrition was calculated based on specific gRNA position used and the genomic coordinates of chromosome copy number transitions as annotated in Table S4."

15. Page 14, lines 301 – 311: very confusing, both text and relevant figures. Very hard to follow and understand. Needs further work to clarify.
Response: We rephrased this section and added a schematic to figure 3 to help understanding.

16. Line 314: could it also be failure to repair because the cut site was in repetitive region within the centromere?
Response: indeed, the failure to repair while progressing through the cell cycle is what we think is the primary problem in the embryo, and discussed extensively in a new Fig. 6 and the discussion section. However, the observations are not limited to pericentromeric regions and are also seen on chromosome arms (chromosome 17, see Fig. S3, chromosome 16, see Fig. 4).

17. Line 317: "… the resulting dicentric chromosomes would break between the two centromeres…" this is an interesting hypothesis that should be further investigated using live cell imaging. FISH
Response: We now add live cell imaging data in Figure 4. (This study required new IRB approval to transport embryos between sites (Columbia and Memorial Sloan Kettering), as well as optimization of conditions, and hence, much time, which is why a revised version of the paper is only available now. The original first author was no longer working on this study.) These studies show lagging chromosomes and dissociated chromosomal fragments.

Together with karyotyping, they support a model of chromosomal attrition secondary to the initial Cas9 lesion. Chromosome bridges were not observed, though UFBs cannot be excluded.
We also added FISH studies in pluripotent stem cells. Performing these in embryos was not successful, but we were able to perform FISH in pluripotent stem cells using breakage probes, which show dissociation of the DSB ends, and lack of separation between sister chromatids.

18. Line 333: “breakage between the cut site and the centromere would result in reciprocal and variably sized tandem duplications and deletions”. Why would the author expect that? Please explain. Why wouldn’t the authors expect to see just a simple loss? Please add schematics to be able to follow
Response: this is indeed incorrect; we removed this statement. For dicentric chromosomes, breakage between centromeres, which can result in inverted duplications and reciprocal loss and gain upon segregation. We write: “Breakage between the centromeres of dicentric chromosomes can result in inverted duplications, which manifest as complementary losses and gains after separation into daughter cells.”
and “ Covalent end joining of sister chromatid ends should equally protect the position of the ends centromeric and telomeric to the Cas9 cut site. Head-to-head fusion would result in covalently linked bicentric chromosomes that form bridges and break during mitosis. Breakage between the centromeres of dicentric chromosomes results in inverted duplications, which manifest as complementary gains and losses after separation into daughter cells. Though we did observe the losses, we did not also observe complementary gains between the centromere and the cut site in sister cells. Instead of duplications, we observed disjointed fragments spanning from the attritted break site to the centromere. Attrition and secondary breakage near the centromere leads to the model shown in Fig. 7. Noncovalent linkage between sister chromatids leading to lagging chromosomes, breakage near the centromere and separation of linked sister chromatids, unprotecting the break site on the centromeric, but not the telomeric side.”

19. Line 336: “... a total of 17 chr X targets”. How did you reach 17 Xs? Are some cells females and some males?
Response: This text is no longer there. The number of targets are based on Table S2 as laid out above.

20. Line 345: “no tandem duplications were found in any sample”. This contrasts with the authors expectations in line 333. Please explain.
Response: the expectation of tandem duplications is simply wrong and was removed. We comment on inverted duplications as a possible result of dicentric chromosomes.

21. What does the author mean by “covalent linkage” or “noncovalent tethering”. Please add clear schematics.
We change our language to end joining, and non-covalent linkage. A schematic is now provided in Figure 7.

22. Lines 346 – 352: the conclusion is not clear. Based on the next paragraph both options (noncovalent tethering and end joining of sister chromatids) occur at the experiment described in this study

Thank you for pointing out this inconsistency. We removed this paragraph and replaced it with text provided above. We cannot exclude end joining does not occur, but what we can say that the events observed are consistent with inefficient repair and persistence of the DSB across multiple assays (molecular and live cell imaging). They are not consistent with covalent ligation of sister chromatids and dicentric chromosomes. Progression through the cell cycle with an unrepaired break and the resulting difficulties during S-phase and mitosis lead to the molecular outcomes observed and the model shown in Fig. 7.

23. In general, better schematics and explanations of figures within the figures themselves are needed across all the manuscript.

Response: We have added additional schematics to Figures, illustrating work flow and/or embryos analyzed.

24. Fig. S1 – before showing examples of aberrations it would be great to see examples of a wt diploid cells. How does the baf analysis looks for a normal karyotype? And the SNP array? With a wt reference the reader can understand better all the other examples and data across the manuscript.

Response: We now include a detailed explanation/examples of SNP array results in Figure S2B and the legend. Thank you for the suggestion.

Minor points:

1. Line 58: "(reviewed in Turocy et al" – missing end parenthesis

Fixed

2. Alpha – should be replaced with α along the manuscript

We do not have alpha any longer

3. Line 265 – did the authors mean Fig. 3E?

The callout was corrected

4. Line 286 refers to Fig. 3g. I'm not sure if I downloaded the correct figure but I do not see 3g. There are 2 panels that are missing their annotations.

Corrected

5. Line 292: "… apparent in 11/12 centromeric…" sentence seems unfinished

Corrected

6. Line 295: refers to fig 3h. Annotation for Fig. 3H is missing in Fig. 3.

Corrected

7. Line 317: no “K” annotation within Fig. 3.
Corrected

8. Line 438 refers to Fig. 3J. There is no 3J in Fig. 3.
Corrected

9. Fig. 3E: add “p arm” and “q arm” to the schematics. Color of hg19 annotated centromere is not obvious and needs to be changed.
This Figure is no longer relevant and was removed because of T2T.

10. Schematics at the bottom of Fig. 3 is not clear and is also missing a letter. Is this 3K? where is the dicentric chromosome and bridge formation in this model? What is the frequency of indels vs. noncovalent linkage/end protection?
Response: this Figure is now a revised Figure 7 mentioned in the discussion section.

11. noncovalent linkage/end protection can simply be called as fusion of sister chromatids at the cut site, to make it more understandable across the paper, together with schematics to show that.
Response: we contrast our model of linkage (e.g. simply through incomplete replication of a broken chromosome as indicated in Fig. 7) with covalent fusion through end joining.
To this point, we now add new data on spontaneous breakages at fragile sites, and how they resemble Cas9 induced outcomes (New Figure 6). Simultaneous cleavage by Cas9 on both sister chromatids may result in end joining and dicentric chromosomes. Sister chromatids at stalled or collapsed forks are not a readily amenable substrate for end joining. We discuss this as follows: “Single-ended DSBs at collapsed forks are not a suitable substrate for end joining due to lack of a partner end. Breakage cycles as observed in Fig. 6A resulting downstream of replication stress-induced breakage may thus be the result of unresolved replication intermediates, rather than of end joining.”

12. Fig. 3i – what are the two lines at the bottom “embryo” and “gDNA control”
Now clarified in Figure Legend.

13. Fig. and Fig. legends: some have capital letters and some small letters across the paper. For example, Fig.S1 has S1a-g, but Fig. S4 has A-C panels.
We now use capital letters throughout the paper.

14. Fig. S3 – I couldn’t find any B in the fig legends or in the manuscript. This Fig needs to be clarified more in the manuscript and in the legends too.
Response: The legend for this Figure, now Figure S5, was extended and a schematic added to understand the embryo that was analyzed. The value of this Figure is that it strengthens the finding of the q arm being affected when guide RNAs are located on the q arm, and pericentromeric changes between the Cas9 cut site and the active centromere location.

Reference:

1 Turocy, J. *et al.* DNA Double Strand Breaks cause chromosome loss through sister chromatid tethering in human embryos. *bioRxiv*, 2022.2003.2010.483502 (2022). https://doi.org/10.1101/2022.03.10.483502

2 Daughtry, B. L. *et al.* Single-cell sequencing of primate preimplantation embryos reveals chromosome elimination via cellular fragmentation and blastomere exclusion. *Genome Res* **29**, 367–382 (2019). https://doi.org/10.1101/gr.239830.118

3 Chavez, S. L. *et al.* Dynamic blastomere behaviour reflects human embryo ploidy by the four-cell stage. *Nat Commun* **3**, 1251 (2012). https://doi.org/10.1038/ncomms2249

4 Palmerola, K. L. *et al.* Replication stress impairs chromosome segregation and preimplantation development in human embryos. *Cell* **185**, 2988–3007.e2920 (2022). https://doi.org/10.1016/j.cell.2022.06.028

5 Voet, T. *et al.* Breakage-fusion-bridge cycles leading to inv dup del occur in human cleavage stage embryos. *Hum Mutat* **32**, 783–793 (2011). https://doi.org/10.1002/humu.21502 [doi]

6 Ramadoss, G. N. *et al.* Neuronal DNA repair reveals strategies to influence CRISPR editing outcomes. *bioRxiv* (2025). https://doi.org/10.1101/2024.06.25.600517

7 Weisheit, I. *et al.* Detection of Deleterious On-Target Effects after HDR-Mediated CRISPR Editing. *Cell Rep* **31**, 107689 (2020). https://doi.org/10.1016/j.celrep.2020.107689

8 Regan, S. B. *et al.* Megabase-scale loss of heterozygosity provoked by CRISPR-Cas9 DNA double-strand breaks. *bioRxiv* (2024). https://doi.org/10.1101/2024.09.27.615517

9 Owens, D. D. G. *et al.* Microhomologies are prevalent at Cas9-induced larger deletions. *Nucleic Acids Res* **47**, 7402–7417 (2019). https://doi.org/10.1093/nar/gkz459

10 Kosicki, M., Tomberg, K. & Bradley, A. Repair of double-strand breaks induced by CRISPR-Cas9 leads to large deletions and complex rearrangements. *Nature Biotechnology* **36**, 765–771 (2018). https://doi.org/10.1038/nbt.4192
https://www.nature.com/articles/nbt.4192#supplementary-information

11 Cullot, G. *et al.* CRISPR-Cas9 genome editing induces megabase-scale chromosomal truncations. *Nature Communications* **10**, 1136 (2019). https://doi.org/10.1038/s41467-019-09006-2

12 Shin, H. Y. *et al.* CRISPR/Cas9 targeting events cause complex deletions and insertions at 17 sites in the mouse genome. *Nature Communications* **8**, 15464 (2017). https://doi.org/10.1038/ncomms15464

13 Alanis-Lobato, G. *et al.* Frequent loss of heterozygosity in CRISPR-Cas9-edited early human embryos. *Proc Natl Acad Sci U S A* **118** (2021). https://doi.org/10.1073/pnas.2004832117

14 Zuccaro, M. V. *et al.* Allele-Specific Chromosome Removal after Cas9 Cleavage in Human Embryos. *Cell* **183**, 1650–1664.e1615 (2020). https://doi.org/10.1016/j.cell.2020.10.025

15 Vázquez-Diez, C., Yamagata, K., Trivedi, S., Haverfield, J. & FitzHarris, G. Micronucleus formation causes perpetual unilateral chromosome inheritance in mouse embryos. *Proc Natl Acad Sci U S A* **113**, 626–631 (2016). https://doi.org/10.1073/pnas.1517628112

16 Liang, P. *et al.* CRISPR/Cas9-mediated gene editing in human tripronuclear zygotes. *Protein Cell* **6**, 363–372 (2015). https://doi.org/10.1007/s13238-015-0153-5

REVIEWER COMMENTS

Reviewer #2

The authors present a revised version of their study describing the consequences of double-strand breaks (DSBs) in human embryos and their impact on chromosomal instability. They demonstrate that pericentromeric DSBs can induce heterogeneous chromosomal outcomes, including telomeric arm deletions or duplications, loss of centric chromosome arms, and interstitial centromeric deletions. They also propose a mechanism to explain the asymmetric genotypic profiles observed. Overall, this manuscript provides novel and important insights into the origin of asymmetric aneuploidy in embryos and highlights the potential risks associated with the use of CRISPR–Cas9 nucleases in human embryos. This is an original and significant contribution to the field.

Compared with the previous version, substantial revisions have been made, and the manuscript has been markedly improved. The experimental data now adequately support the authors' conclusions.

• The title is now better aligned with the experimental evidence and accurately reflects the results.
• The proposed mechanism of non-covalent tethering (now referred to as "persistence of unrepaired DSBs") between sister chromatids is more thoroughly investigated and is now convincing.
• Numerous additional experiments have been included. Live-cell imaging, PCR, and FISH analyses were performed to test and refine the proposed mechanistic hypothesis.
• The reference list has been expanded. However, references addressing spontaneous aneuploidy in human induced pluripotent stem cells (hiPSCs) during culture should be added.
• The original manuscript was difficult to follow. The revised text, together with the schematic in Figure 7, is now much clearer.

This revised version of the manuscript is suitable for publication.

Author's Response: We thank the reviewer for these encouraging comments. We also thank the reviewer for the highly detailed criticism of the manuscript. This is a significant contribution, which is much appreciated.

However, I have **several minor comments** that could further improve the readability of the manuscript and the clarity of the figures:

• Figure 1G: To facilitate understanding, please define “CCF” in the figure legend.

Author’s Response:
We explained CCF = cytoplasmic cellular fragment, in the legend related to **Figure 1G**.

• Figure 2A: Please indicate the DSB on chromosome X.

Author’s Response:
We now indicate a Cas9-induced DNA DSB on chromosome X to the scheme in **Fig. 2A**.

• Figure 2C: Please indicate the number of cells analyzed to clarify the correspondence with the 2D representation.

Author’s Response:
We added blastomere numbers to **Fig. 2B**, **C** to match each SNP array analysis with its corresponding schematic and with the gel in **Fig. 2D**.

• Figure 4A: Please specify which chromosome(s) are targeted.

Author’s Response:
We specified the targeted chromosome(s) in the schematic shown in **Fig. 4A**. It is chromosome Xp throughout the Figure.

• Figure 4G, I, K, M: Please specify which chromosome is analyzed.

Author’s Response:
We specified the targeted chromosome(s) in all panels of **Fig. 4**.

• Figure 5:
5A: Please specify hESCs in the experimental scheme.

Author’s Response:
We specified the experimental system in **Fig. 5A** as hESCs and the title of the figure legend.

5C: Please clarify what is meant by “distance between red/green probes.”

Author's Response: We added distance measurement to the schematic and re-labeled the vertical axis of Fig. 5C and E. We clarify in the text that the distance was measured between FISH probes centromeric and telomeric of the Cas9 cut site.

5D: Please clarify that "linked SNP" corresponds to the non-targeted allele, and that EYSfs corresponds to the targeted allele.

Author's Response:
We added a new schematic to **Fig. 5D** to indicate targeted EYSfs and linked SNP and PCR primers, clarifying linkage.

5E: Please clarify what is meant by "distance between …".

Author's Response:
We clarified that the distance was measured between either duplet signals of FISH probes (red-red) or (green-green), representing sister chromatids (**Fig. 5E**).

• Figure S1A/B/C: Data from different loci are combined. Please specify the targeted loci directly in the figure.

Author's Response:
We specified the targeted loci in panels of **Fig. S1**.

• Figure S2: This figure is not sufficiently described or explained in the text (line 135). Please guide the reader through all panels.

Author's Response:
"We added new text explaining this Figure. Quoting from manuscript: "Interestingly, one embryo, consisting of 4 cells (**Fig. S2A**) showed mosaic correction of meiotic trisomy 16 in SNP array analysis. This supplemental Figure also serves to explain SNP array data analysis: SNP signal intensity can be quantified as chromosomal copy number bar plots for the entire genome, for balanced signal intensity across a single chromosome, for allelic ratio, and for heterozygosity (**Fig. S2B**). Such analysis captures most relevant information regarding megabase-scale chromosomal changes, with on target analysis being performed by PCR and Sanger sequencing. For the aneuploid embryo with a gain of chromosome 16 originating from the second meiosis (**Fig. S2C**), trisomy correction occurred in blastomere 3 and 4 (**Fig. S2D, E**)."

Other minor comments:

• Line 118: The term “chromosomal error” is unclear. Please clarify.

Author’s Response: we specify segmental or whole chromosome loss.

• Line 152: Embryos without Cas9 represent an appropriate control to assess spontaneous breakage events.

Author’s Response:
The distinction of spontaneous breakages in chromosome-containing cellular fragments from Cas9 induced breakages is based on predicted potential off target gRNA targets. We state that “None of these [spontaneous] sites were in proximity to a predicted off-target site and are consistent with the pattern of spontaneous breakages at long neuronal genes (Palmerola et al., *Cell.* 2022).” This latter study serves as a reference point for unmanipulated embryos.

• Line 252: Please specify which PCR results are being referred to (1–2 or 1–3).

Author’s Response:
We clarified in the text and the figure legend that the point discussed relates to detection of potential head-to-head end joining of sister chromatids, for which the single primer (#1) was utilized in the PCR reaction.

• Lines 219–223: The statement “Asymmetry … chromatids” represents a hypothesis rather than a conclusion and should be moved to a more appropriate section of the manuscript.

Author’s Response:
This statement was deleted, as redundant with the discussion.

• Using consistent terminology for chromosome fragments may improve readability. Consider choosing between “centromeric-containing/centric” and “telomeric/acentric” throughout the manuscript.

Author’s Response:
We substitute centromere-containing with the more concise ‘centric’.
To indicate directionality relative to the cut site, we use ‘centromeric’ or ‘telomeric’ of the cut site.
For chromosome fragments without a centromere, we use ‘acentric’, and with a centromere, ‘centric’.

• Do the authors observe chromothripsis in micronuclei? If so, please discuss this point.

Author's Response: In Fig. 1B we quantify fragmented chromosome changes that resemble chromothripsis (lines 123-124). We did not specifically isolate micronuclei in this study to evaluate chromothripsis, nor evaluate potential stable rejoining of shattered chromosomes. A strong conclusion on this issue may need evaluation at different time points, which is why we are conservative in our statements.

Reviewer #3

Asymmetric Chromosomal Consequences at Double Strand Breaks in Human Embryonic Development

Turocy et al., 2026

DNA repair mechanisms in the human embryo remain poorly understood and DSBs can have adverse consequences, including chromosome loss. In this revised study, the authors test the effects of CRISPR/Cas9 mediated genome editing on genome stability in human embryos. The study show concerning results where 1/3 of the CRISPR/Cas9 targeted chromosomes in human embryos show either whole, segmented, or fragmented chromosome loss. Moreover, the chromosomal alterations were asymmetric relative to an induced DSB: acentric arms showed complementary gains and losses, and overall were mostly lost, while centric arms were biased toward losses.
Using a variety of methods, the study shows that broken chromosomal fragments that contain centromeres are lagging during mitosis, and that their segregation is often abnormal and can result in exclusion of the chromosome fragment from the cell, secondary breakage and attrition of genomic material between the cut site and the centromere. In contrast, acentric chromosomal fragments do not show an additional break or attrition at the CRISPR/Cas9 cut site. These results highlight the risks of chromosomal-scale changes in CRISPR/Cas9 genome editing and reveal how a single DSB destabilizes am entire chromosome in the human embryo. Lastly, the author propose that these asymmetric differences are arising because the original DSB was not repaired. Given the potential of CRISPR/Cas9 mediated genome editing in treating inheritable genetic disorders it is important to evaluate its genomic effects in human embryos. This study reveals that clinical use of CRISPR/cas9 genome editing in the embryo is too genotoxic. They also show that DSBs occur spontaneously in the human embryos and that the consequence of induced DSBs are similar to spontaneous DSBs.

I appreciate the authors taking the time to significantly improve and substantiate their data using different approaches, including live-cell imaging that I proposed. I also think that they have answered most of my comments and have improved the work significantly in the revised version.
While the results of this study are important for the broad audience, and the experiments are mostly sound, I'm still left with several major issues that should be addressed.

The main issue in my view is novelty. I'm trying to understand what is the main take-home message and is it new? For example, line 335 says: "thus, sister chromatids

remain in proximity, while DSB ends are prone to separate from each other". I'm not sure what is novel in these results. It is widely known that an acentric fragment will get lost, and if a fragment contains a centromere, the centromere will mediate segregation and separation. Can the author expand on the novelty of their results across the manuscript compared to what is known in the field as consequences of DSBs in somatic cells and in embryos. For example, do DSBs tend to be repaired more efficiently in somatic cells? Can they compare between the two and demonstrate inefficient repair in embryos?

Author response: The novelty of the study is at least threefold.
i) The finding that chromosomal aneuploidies are common when DSBs are introduced at different locations of the genome. Upon our initial publication Zuccaro et al. 2020, focused on a single location, many still expressed some reservations, pointing to studies concluding efficient interhomolog repair (we points out that the data in Ma, Mitalipov et al. could also be explained by chromosomal loss).

ii) the main novelty is in the asymmetric genetic consequences relative to the break site, which are linked to the presence or absence of a centromere, and the processing of DSB ends. This is enabled in this specific system through the proximity of karyotype analysis within a single cell cycle after DSB induction. The finding of secondary breakage and simultaneous attrition at the break sites shows a new mechanism of how an entire chromosome is destabilized in the absence of a fusion-breakage cycle. This is supported by Fig. 2 and Fig. 3 predominantly at a molecular level, and by Fig. 4 using live cell imaging.
We now emphasize these novel points in a revised abstract. We point out that it is not a priori known that these fragments are not attached to another chromosome through translocation. This is shown through both live cell microscopy in Fig. 4 and through analysis of excluded chromosome fragments from the embryo. We think that there is value in directly showing that these are dissociated, even if it is consistent with expectations from other cellular systems.

iii) there is not currently a comparison of DSB repair efficiency in different somatic cells. Generally DSB repair is slow. The main difference may be checkpoints. We discuss literature showing that somatic cells However, we do cite literature showing that chromosomal changes in somatic cells occur after G2 checkpoint inhibition similar to human embryos (REF. 25), and slow repair in neurons and ESCs (Ref. 28). Text on lines 537-546.

In addition, while improved, figure legends and text need further review and refinement.

Major points:

1. Novelty –What is the main take-home message and is it new? Can the author expand on the novelty of their results across the manuscript compared to what is known in the field as consequences of DSBs in somatic cells and in embryos.

Response: (also see above). In the revised abstract we now write:
"Secondary breakage at the centromere and simultaneous extensive attrition at the DSB site indicates that for at least some events, a DSB destabilizes the chromosome without requiring end joining of sister chromatids".
Please also note that we do not observe inverted duplications mirroring the losses/attrition towards the centromere. This is a significant addition to the known mechanism of fusion-breakage cycles, which involve covalent fusion of sister chromatids.

2. This work, as is, shows the consequences of DSBs in embryos, and the lack of their repair in many instances. However, there is no attempt to explain that mechanistically. What causes embryos to lack in their repair capacity.

Response: In the discussion, we link it to weak checkpoint control: "The absence of a delay points to weak DNA damage checkpoint control, allowing the cell to enter mitosis with one or even several broken chromosomes." We also point out that in somatic cells, weakening of checkpoints results in similar outcomes (REF28). However, experimentally manipulating checkpoints requires a dedicated body of work in a separate study.

3. Fig. 2D – Can a PCR amplify a pericentromeric repetitive region? A positive control from a wt cell is missing. Also, what is "other"? no explanation is given within the fig legend.

Response: the 'other' are positive controls, which are now appropriately labeled. Two cells from different embryos remain labeled as 'other', which is now explained in the Figure legend.

4. Figure legends across all figures should be improved to include all necessary details. For example, the legend of Fig. 2D is not sufficiently detailed to understand the data shown. The meaning of "other" is not explained. Fig legends should be detailed and include number of repetitions, and explanations of samples names, etc.

Response: we thoroughly screened and edited the Figure legends and improved details and descriptions.

5. Line 209: can the attrition be attributed to technical difficulties in sequencing/SNP analysis of that region? Can some SNPs be harder to amplify? If yes, this should be included in the explanation of study limitations.

Response: There are several lines of evidence arguing against artifacts. First, there is no reason why SNPs in close proximity on either side of the DSB should be more or less amplifiable. Second, there are clear distinctions between different samples. We show this for instance in sister cells of Fig. 2C. Two sister cells show attrition, the other pair does not. These are biologically meaningful differences rather than sporadic artifacts. Importantly, our calling of attrition does not rely on a single SNP, but on a transition of signal intensity between numerous SNPs. The transition point provides coordinates, the flanking SNPs validate the presence or absence of DNA. This adds robustness to the analysis.

6. Line 249: "sister chromatids appeared as separate visual signals in mitosis … suggesting they were not a covalently linked entity". – why would the authors expect sister chromatids to be covalently linked? Sister chromatids are held together at the centromere by cohesin. The broken arms do not have a centromere and are not supposed to be held together. I'm not sure I understand the novelty of these results.

Response: this is an important point, and apparently this was not well explained. We now write: "The physical proximity of sister chromatids as seen in FISH lend sister chromatid ends to illicit repair, resulting in head-to-head fusion, dicentric chromosomes and breakage fusion bridge cycles." Such cycles, a possible mechanism of chromosome destabilization as observed here, involve covalent fusion.
Our data such as those in Fig. 2 and 3, as well as the separation in Fig. 4, are inconsistent with head to head fusion and with breakage fusion bridge cycles.

7. Additional schematics panels will improve the understanding of current Fig. 4E and 4G.
Response: We added an additional schematic.

8. Fig. 5B is not annotated. What does each microscope image represent?? What is control, what is Cas9 treated, colors of probes etc. Basic Figure preparation and revision is absent.
Response: We added relevant info to the Figure panels.

9. The authors propose that if the DSB is not repaired, than it will likely progress into linkage of the centric fragments resulting in a lagging chromosome with two

centromeres that is different than a dicentric chromosome that arise through break-fusion-bridge mechanism. Indeed, the break-fusion-bridge mechanism should lead to duplications, which are not observed in the results presented here, reducing the chances that what we are seeing are indeed dicentric chromosomes. Perhaps long-read sequencing could help resolve the structure of the derivative chromosome observed here.

Response: We are glad this distinction came across. The hallmark of the broken chromosomes we observed is that they are not an intact DNA strand, neither joined to its proper repair partner, nor joined through a translocation disjointed from the proper repair partner. Sequencing reads are not expected to span across. Our analysis is focused on SNP arrays, which provide most, but not all structural information. We include this in the limitation of study.

10. The author should clarify across the text what is the difference between fusion of the centric fragments that lead to dicentric chromosome compared to linkage of the sister chromatids that also results in a dicentric structure as their model propose. This is central to their work and their model but is not easy to understand and discriminate between the two.

Response: We edited Figure 7, which shows the model of noncovalent linkage, based on an unrepaired DSB.

Minor points:

1. Line 118 "… due to a chromosomal error on the other chromosome". I suggest rephrasing 'chromosomal error' to arm loss or segmental loss.

Author's Response: We rephrased the text and specified the event as a segmental loss…

2. Line 153: "breakpoints .. are located at WWOX, an olfactory receptor gene cluster, WDR25, DPP10, RPTOR…". Are these known as fragile sites? if yes, it would be worth to mention it in the text.

Author's Response:
We now state that WWOX and DPP10 are known fragile sites, while the other sites match the pattern of spontaneous breakage in human embryos at long genes and OR gene clusters (**REF 8**).

3. Fig. S4 – no individual panels (A, B, C…) make it harder to follow the text. Also, addition of chromosomal schematics can be helpful in interpreting the sequencing data.

Author's Response:
We now changed the data to the T2T genome assembly, which makes the figure more streamlined and cleaner. We now have 3 panels.

4. Fig. S5 - addition of chromosomal schematics can be helpful in interpreting the sequencing data.

Author's Response:
Chromosome schematics are at the top the column of SNP array data for 8 blastomeres. Different blastomeres are now more clearly labeled.
As in Fig. S4, we performed a new analysis according to the T2T assembly, because of the inaccurate gRNA location in hg38. This simplifies the Figure and facilitates understanding.

5. Legend of Fig. 4D is not complete and is unclear. Many details are missing.

Author's Response:
We further improved both **Fig. 4D** and the corresponding legend.

6. Lines 261 – 263:" not all expected genomic material…" sentence is not clear. Please rephrase.

Author's Response:
We simplified the language: The loss of an acentric Xp fragment, as well as untargeted chromosomes 13, might have been due to the exclusion from the cell, or a consequence of mechanical cell splitting.

7. Paragraph related to Fig. 4F-M (p. 13-14) is unclear. Please rephrase.
Response: The section on live cell imaging was rephrased. The main point is that cells resulting from mitosis with lagging chromosomes show attrition of genomic material between the Cas9 cut site and the centromere.

8. Line 289: "two chromosomes were again apparent" – please change to DNA entities or fragments as one cannot know from microscopy if these are full chromosomes or fragments.
Response: Thank you, this is a good point. We write 'chromosomal entities'.

9. Line 292: "… can get expelled from dividing cells, resulting in bias of spontaneous … towards chromosome loss" – micronuclei do not have to be expelled. The DNA they contain can also be fragmented and lost.

Author's Response:
We now simply state: "The unstable genetic material in micronuclei is lost, contributing to the bias of spontaneous mitotic abnormalities towards chromosome losses[8]. We remove the term 'expel', as we cannot say if active or passive, and we also do not know if the material in Fig. S6A lost from the blastomere was fragmented or not.

10. It is not clear to me what can be learned from Fig. S6D. It is completely blurry.

Author's Response:
We converted the image to black and white (which is the color of recording) and adjusted contrast for both panels D and E.

11. Fig. 5D missing Y axis

Author's Response:
We added the missing axis label.

12. Line 409: "the nature of this linkage may be end-joining of sister chromatids resulting in bicentric chromosomes". The correct term is 'dicentric'. In addition, lagging chromosomes can be a result of erroneous microtubule attachment (merotelic attachment). They do not necessarily represent dicentric chromosomes.

Author's Response:
We corrected the text using the appropriate term 'dicentric'.
We also add: "lagging chromosomes **caused by Cas9 cleavage** observed by live cell microscopy…"
The second part is an important point. The combination of Cas9, live cell microscopy and karyotyping links these three aspects. While we cannot conclude that spindle-chromosome attachment is normal it would not be the primary event. This is an interesting question, which we suggest is best pursued in a separate study.

Reviewer #4

The article addresses the consequences of CRISPR/Cas9 editing in human embryos and the subsequent small and large scale errors produced. The induced chromosomal instability via DSB formation provides an excellent view into the limitations of DNA repair within the human embryo. The authors have done a thorough job addressing the comments from Reviewer #1, however, there are a few points within the revised draft that I would like to see resolved (see below).

Major Comments:
Reviewer #1 comment: The statement/claim that the functional centromere can be mapped, seems preposterous. The data does not fully support the claims as other possible reasons for the observations can be deduced. The position of the guide is based on the reference genome. The pericentromeric regions are very variable amongst individuals. How sure are the authors that the pericentromeric region in the embryo genomes are similar to the reference genome? Structural variation in the region would change the outcome and lead to a different interpretation.

Author's response:
This section was extensively revised and is now a minor point. We now have access to a T2T genome assembly. The gRNA which maps to the p arm on hg19 and hg38, maps to the q arm on T2T. Our data suggested that the gRNA targeted the q arm, as the q arm was primarily affected by segmental aneuploidies. We write: "One gRNA site was located on the p arm of chromosome 16 according to the hg38 genome assembly (Chr16:35126545 at 16p11.1), and to the q arm according to the T2T genome assembly 16q11.2 at chr16:39002348. The q arm was primarily affected by Cas9 cleavage at that site, congruent with the location relative to the centromere according to T2T (Fig. S4). At the time of gRNA design, the T2T assembly was not yet available – frequent q arm changes are validating T2T."
Of note, we now display SNP array data of Fig. S4 and S5 in the T2T assembly. This should make it significantly easier for the reader.

Reviewer #4 comment:
I have some concerns about this change. Conducting a BLAST search of the gRNA sequence provided in Table S1 against the T2T-CHM13v2.0 genome using the NCBI Genome Data Viewer, the browser places the sequence mostly within the centromeric region but is still located on the p-arm. However, when using the UCSC Genome Browser, the browser places the sequence in 16q11.2. My look into this was very quick (and likely simple to explain), but I would appreciate a slightly deeper description of this change.

Response: We did rerun the analysis and the coordinates and BLAT search on UCSC genome browser is congruent on 16q11.2 at chr16:39,002,348-39,002,370. We do not know the reason for the above discrepancy.

Additionally, if we assume that the Cas9 cut-site has switched from the p-arm to the q-arm I am confused by some conclusions made with the SNP maps. When looking at chromosome maps in Fig. S4 (annotation from hg19 and centromere placement from T2T), it is stated that there has been a "loss of genomic material between the Cas9 cut site and the centromere…" (lines 196-197). However, with the hg19 and T2T combination this conclusion is based on a cluster of deleted SNPs that would (if located on the q-arm) theoretically fall within the highly repetitive proximal region of chromosome 16q which has been a clear gap in all historical assemblies. I am questioning whether combining the alignments like this is doable or if it is leading to skewed conclusions. I understand that it would be a particularly large undertaking, but for any conclusions based on the Chr 16p arm* gRNA the entire SNP map should be converted to the T2T alignment.

Response: This is certainly correct. We have now reanalyzed the data using T2T and are now displaying both Figures S4 and S5 with T2T alignment. The display items are now far cleaner and easier to present and interpret. The conclusions do not change with this altered presentation.

Minor comments:
Reviewer #1 comment: Intro line 79 'Furthermore, microdeletions and microduplications near centromeres can give rise to disorders such as 16p11.2 microdeletion or 16p11.2 duplication syndrome which have been associated with an increased risk of intellectual disability and psychiatric disorders': Mechanistically, those genomic disorders are the result of NAHR and do not contribute to the argument that pericentromeric regions are more frequently involved in rearrangements. Other, non-pericentric genomic disorders are even more frequent (e.g. 22q11).

Author's response:
We now include reference to deletion syndromes on chromosomal arms, including 22q11. The reason why we specifically mention 16p11.2, is because we have guide RNAs targeting those areas.

Reviewer #4 comment: The authors state that deletions and duplication at 16p11.2 are referenced due to the gRNAs used in this study and not as a

pericentromeric argument. However, the 22q11.2 deletions are then listed as an example that they can “also occur on chromosomal arms…”. I find this wording confusing because the breakpoints for 16p11.2 and 22q11.2 are nearly the same distance from their respective centromeres and are not necessarily different categories of breakpoints.

Author’s response: we removed the reference to diGiorge syndrome and 22q11.2, as it is also close to the centromere. The relevant revision to the point of reviewer 4 is: “Deletions and duplications may arise through abnormal repair of DNA breaks introduced during either meiosis or during cell cycle progression in the early embryo.”

Additional Reviewer #4 comments:
The authors stated that between the initial submission and re-submission there have been changes in the scope and data of the manuscript, as well as in the personnel involved in the project. Unfortunately, this is visible in the manuscript. Throughout the text there are several points where values do not add up and/or do not match figures, incorrect figures are referenced, or in some cases reference figure panels that are no longer included. I have included a list of some edits below that need to be addressed before publication. It is incredibly difficult to maintain an overview on manuscripts of this complexity and prolonged duration, therefore I would suggest that the authors find a fresh set of eyes to comb through the text and perhaps rework the supplemental tables for clarity, allowing the reader to more easily validate findings.

Table S2:
• Cells J48 through J68 are showing incremental values and not the original gRNA value (ChrXp: 58089779)
Thank you for noticing, indeed, now corrected.

Table S3:
• The numbers for this table do not match the text.
- In text – 10 LOH, 18 no amplification, 8 complex (lines 90-95)
- In table – 9 LOH, 21 no amplification, 6 complex
Author’s response:
The near haploids are LOH and this label was added in the legend. It is 10
The no amplification is 20, corrected in text.
8 are complex, 2 of which are also LOH, which is now stated in the text.

• Sequenced chromosome containing cellular fragments
- The “chromosome containing cellular fragments” are labelled in column D, however, these fragments do not correspond to the “sample type” reported in column B. 4 of the 5

“cellular fragments” are listed as blastomeres (C2-3_16qXq, D1-4_16qXq, G2_1_Xp, and G2_5_Xp).
Author’s response: The sample type is called at time of collection by cytological appearance. Some fragments can be large and appear indistinguishable of a blastomere. We therefore changed the term to blastomere-like. We added additional text to the Table caption to clarify this.

- In text – 5 chromosome containing cellular fragments/cells (line 95-97)
Author’s response: This changes to six, one was missing.

- In text – 5 embryos with fragments (matches line 145) (now line 158)
Author’s response: This changes to six.

- In table – 6 chromosome containing cellular fragments/cells from 5 embryos
Author’s response: It is now 6 embryos

Figure 1:
• (D) Did you look at your samples the other way? I.e. was loss of chromosomes X more likely to occur when embryos were injected with gRNA targeting chromosome X?
Author’s response: Fig.1D was removed. While we do not observe whole chromosome 16 loss in chrX targeted embryos, the numbers are too small for statistical significance. We now only evaluate whole chromosome 16 loss in the context of a trisomic embryo with mosaicism for segmental aneuploidies (**Fig. S2**).

• (G):
- Again the “fragments” that are denoted here need to be somehow highlighted in Table S4.
Author’s response: Column D adds ‘Fig. 1F’ to the relevant samples.

- It took a while to determine which embryos are referenced here, but it looks like there is a fragment that has not been reported. Embryo I1_F_XDMD_Chr16pprox_82.CEL is listed as also containing a breakpoint on chromosome 8 in WWP1 (Table S4, Line 102). This fragment is not denoted in Fig. 1G, nor in the text describing the breakpoints (lines 152-158). When double checking the breakpoint (chr8:87386539-87404553) in T2T it doesn’t seem to correspond to WWP1 but to a series of repeat sequences, which is also of interest due to increased potential for repair errors.
Author’s response: the genome coordinates provided in Table S4 is from hg19 (indicated in the header of table). It is the break point in Fig. 1F (formerly 1G) at 8q21.3. We added the gene WWP1 name to panel Fig. 1F.

Minor text changes:
• Line 86: delete extra "in"
Author's response:
Thank you

• Lines 90-95: double check numbers
Response: same point as above

• Line 95: double check numbers (6 chromosome containing fragments from 5 embryos)
Response: same point as above

• Line 102: I don't know where 260 comes from. By counting the number of gRNA in each of the 113 embryo cells (Table S2) I only count 244 (gRNA 1: 113, gRNA 2: 77, gRNA 3: 54). Am I calculating this incorrectly?
Response: The count is based on molecularly distinguishable genetic outcomes on diploid embryos. We carefully recounted the data in Table S2. In total, the data of pericentromeric gRNAs amount to 273 distinguishable Cas9 induced outcomes and 15 wt outcomes (total 288 outcomes). This number differs from the total number of 394 evaluated chromosomal targets due to homozygosity or hemizygosity of indels, as well as lack of an on target PCR product. Fig. 1B is adapted to these changes, and is now also displaying chromosomal gains and losses separately as they are molecularly distinguishable. The conclusions remain unchanged. We explain this as follows:

"Since blastomeres are diploid, each blastomere contains two homologous chromosome 16s and either one X chromosome in male embryos (XY) or two homologous X chromosomes in female embryos (XX). The gRNAs for chr16 and chrX were not designed to be allele specific, thus targeting both homologous chromosomes, and were injected either alone or in combination as indicated in Table S2. Such targeting can result in a cell carrying heterozygous or homozygous indels, an indel combined with a chromosomal change, or no chromosomal material at all. The combined number of autosomal and X chromosomal targets in 108 embryo cells is 394 chromosomes, of which 288 molecularly distinguishable Cas9-induced outcomes were detected by PCR and Sanger sequencing (157/288) or chromosomal changes detected in SNP array analysis (131, composed of 107 losses and 24 gains) (Table S2).

Indels were found in 49% (142/288) of detectable and distinguishable outcomes, of which 67 were hemizygous or homozygous, representing either two identical indel

events, or loss of heterozygosity. 5% (15/288) were found to be wild type (Fig. 1B). Thus, the pericentromeric gRNAs used demonstrated a high on-target efficiency. By far the most frequent indel among all samples in our experiment using human embryos was a -1 base pair (bp) deletion and a +1 bp insertion, which may be the result of processing the stagger created by Cas9 cleavage 23 (Fig. S1A). Gel electrophoresis showed smaller PCR products (Fig. S1B), which were confirmed to be the result of CRISPR-Cas9-induced deletions by Sanger sequencing. Indels ranged from -358 bp to +2 bp (Fig. S1A). Using primer pairs of up to 1.3 kilobase (kb) PCR in size, only two Indels were larger than 100 bp (Fig. S1A). Deletions of 6 bp or larger in human embryos occurred primarily through microhomology-mediated end joining (MMEJ) (Fig. S1C). These studies confirm the high level of DSB induction and genetic modification at the evaluated sites and point to unrepaired or misrepaired chromosomes as a frequent outcome. Because both chromosomes were targeted, we observed indels with LOH due to a segmental loss on the other chromosome (Fig. S1D).

Lack of a PCR product was also common: 60 evaluated sites showed no specific product. The lack of a PCR product can be caused by several events, either technical or biological, including unrepaired chromosomes and aneuploidy. Lack of a PCR product and hemi- or homozygous indels account for the difference between 394 chromosomal targets, and the 288 molecularly identifiable genetic outcomes.

Chromosomal abnormalities were very common: 45% (131/288) among a total of 288 molecularly identifiable Cas9-induced outcomes, demonstrated a gain (8%, 24/288) or a loss (37%, 107/288). Losses were composed of whole chromosome loss 24/288 (8%); segmental chromosome loss 77/288 [27%]; 6/288 [2%] fragmented chromosome loss, resembling chromothripsis) (Fig 1B). Chromosomal changes were evidenced by both reduced copy number and loss of heterozygosity on SNP-array-based chromosome screening. Reduced signal intensity and altered allelic ratios on either side of the targeted locus indicated segmental chromosome abnormalities (e.g., Fig. 1C, Fig. S2). The break points were noted to occur at the Cas9 cut site; thus, segmental losses can readily be attributed to CRISPR-Cas9 activity."

Table S2 legend: Indels at the targeted genomic sequence generated by Cas9 RNP with gRNA, based on Sanger sequencing. Samples with two complete homologous targeted chromosomes can have two different indels. Indels on maternal and paternal chromosome are separated by a dash (n/n) and indels where two chromosomes are present based on SNP arrays but no heterozygosity is detectable are indicated by double asterisk (**, n=22 instances). These may be two homozygous indels or only one of the two is detectable by PCR and Sanger sequencing. A size-neutral indel refers to a

nucleotide insertion and deletion of equal value resulting in a net zero indel. Detailed chromosomal content based on SNP array analysis.
Polar bodies were used to evaluate meiotic aneuploidy.
A gRNA targeting chr16 centromere (hg38, chr16c:36221387, italics in column K and L) was excluded from indel efficiency analysis in Fig. 1B because of a frequent mismatch compromising its activity." This centromeric gRNA was also removed from the schematic of Fig. 1A.

• Line 113: The text states that three Indels were larger than 100bp, but Fig. S1A only shows two events larger than 100bp (-358 mmej and -132 nhej).
Response: It is two, thank you

• Line 121: update denominator to 260
Response: Now 288, corrected

• Line 144: Fig. 1E should be Fig. 1F.
• Line 147: Fig. 1F should be Fig. 1G.

Author's response:
We now refer to the respective **Fig. 1** panels correctly in the text.

• Line 221-223: Typo in sentence grammar.

Author's response:
We corrected the typo.

• Line 251-252: There is no Figure 3D.
Author's response:
We changed callouts and this is now panel E, now referred correctly in both the text and corresponding legend.

• Please provide more consistent labeling of stages in figure 4.
Author's response:
We added schematics and additional labels.

• Line 308: check punctuation
Author's response:
We corrected the text.

• Line 353: missing word

Author’s response:
We added the missing word.

• Lines 380-382: I understand that the authors are attempting to broaden the context of these findings, however regarding human embryos as a model for other biological systems seems inappropriate and incorrect.
Author’s response: We change to ‘experimental context’ to remove the word ‘model’.

We would like to point out that there has been basic insight into mammalian embryo biology based on work in the human embryo: for instance, mammalian embryo replication stress, and frequent chromosomal consequences based on a single DSB in work that we are citing. Traditional model systems like the mouse embryo or even somatic cells, followed suit in these cases (e.g. PMID:33125898; PMID:34615869; PMID:33846636, or Ref.8). Which is also the reason why initial studies on Cas9 induced mutation correction were so thoroughly misunderstood (PMID:28783728).

• Lines 388-391: Here you state that from your observations it is likely a ‘lack’ of repair rather than abnormal repair, but also shows evidence of ‘inefficient’ repair. With this word choice these two statements seem counter to each other. I would consider ‘inefficient’ repair to be present but not functioning properly and not a ‘lack’. Consider re-wording.
Author’s response: We reword to: “Failure to repair prior to mitotic entry is supported by the observation of dissociated chromosome fragments in live cell microscopy and the corresponding loss of chromosome segments from the embryo in karyotype assays.”

• Line 392: Consider adding that these errors occur “in mouse embryos and in somatic cells” for clarity.
Author’s response:
We included the suggested text in the revised manuscript.

REVIEWERS' COMMENTS

Reviewer #2 (Remarks to the Author):

The authors have addressed my major comments, and the manuscript is now clearer and easier to read. Overall, I believe it could be suitable for publication, provided that the following remaining points are adequately addressed.

Major point:
The title remains somewhat vague and does not fully highlight the originality of the study. In particular, the key novel aspects appear to be asymmetric attrition and secondary chromosome destabilization. I would therefore encourage the authors to revise the title to better reflect these contributions. For example: “Asymmetric Attrition and Secondary Chromosome Destabilization after Double-Strand Breaks in Human Embryonic Development.”

Response: thank you much for suggesting, this, wonderful title, which we adopted.

Line 78:
Single versus multiple double-strand breaks (DSBs) can trigger distinct repair mechanisms. However, the main conclusion of the manuscript focuses on the consequences of an unrepaired DSB (see Fig. 7). In this context, the experiment using a non-specific gRNA inducing multiple DSBs on chromosome 16 seems less appropriate and may not be fully consistent with the central message of the paper. I would suggest removing this experiment from both the main text and the figures, or alternatively, providing a stronger justification for its inclusion.

Response: We applied this gRNA in the context of removing a trisomy 16 (Supplementary Figure 2). We now write: “A guide RNA targeting 3 sites on chromosome 16p11.2 was used for targeting the trisomy. Targeting a chromosome at three sites simultaneously resulted in efficient chromosome changes (53%: 28 chromosomal changes of 52 target chr16 in 24 cells analyzed, of 8 diploid and 1 trisomy embryos, Supplementary Data 2). Nevertheless, only half of all targeted chromosomes showed altered copy number frequency. Thus, Cas9-induced whole chromosome loss is a consequence of on-target cleavage in human embryos and may potentially be useful for the correction of trisomies, but these will be mosaic. “

Minor points:

Line 102: “16s” should be corrected.

Corrected

Lines 138–139: The sentence is unclear and would benefit from rephrasing.
Rephrased as: "Chromosomal changes are apparent as reduced copy number and loss of heterozygosity on SNP-array-based chromosome screening. Reduced copy number and altered allelic ratios on either side of the targeted locus are segmental chromosome abnormalities (e.g., Fig. 1C, Supplementary Fig. 2)."

Figure 1C: There appears to be a loss of the non-targeted arm of chromosome 16. Could this reflect an off-target effect or an issue with the gRNA/DSB location? Please clarify and provide an explanation.
We now write: "Fig. 1C shows the presence of an acentric fragment and the loss of a centromere-containing chromosome segment." This is an on-target consequence.

Reviewer #3 (Remarks to the Author):

Asymmetric Chromosomal Consequences at Double Strand Breaks in Human Embryonic Development

Turocy et al., 2026, revised version

I have reviewed the revised manuscript by Turocy, Jerabek et al. and the authors' responses to my previous comments. I am satisfied that the majority of my concerns have been addressed, and I appreciate the significant effort that has gone into this revision.
The authors have substantially improved the manuscript. The addition of live-cell imaging, FISH analysis, and the revised mechanistic model in Figure 7 have considerably strengthened the study. The figure legends are clearer, the terminology is more consistent, and the logic connecting the experimental observations to the proposed model is now easier to follow.

Response: Thank you for the review of this work across different versions and your input.

I am recommending acceptance of this manuscript, with one remaining comment for the authors to consider:

Regarding repair efficiency in embryos versus somatic cells: I had previously asked whether the authors could provide a direct experimental comparison of DSB repair efficiency between human

embryos and somatic cells, as this would substantiate their claim that inefficient repair is the primary driver of the chromosomal consequences observed. The authors have addressed this point by citing relevant literature (refs. 25 and 28) showing that similar chromosomal outcomes occur in somatic cells when checkpoint kinases are inhibited, and that repair kinetics are slow in neurons and pluripotent stem cells. While I accept that a dedicated experimental comparison is beyond the scope of this study, I would encourage the authors to be explicit in the text that the comparison to somatic cell repair is based on cited evidence rather than data generated in this study. A brief clarifying sentence in the Discussion to this effect would help readers calibrate the strength of this specific claim.

Response: we now clarify this point. Though these referenced datasets are not directly comparable, using different gRNAs and delivery systems, the frequency of aneuploidy in human embryos described here is much higher. In somatic cells…

Overall, this is an important and timely study that provides novel mechanistic insight into the chromosomal consequences of double-strand breaks in human embryos, with clear implications for the safety of heritable genome editing. I support publication in Nature Communications.

Reviewer #4 (Remarks to the Author):

I thank the authors for addressing the discrepancies in numbers and for including additional text for clarity. There are a few minor final points that I would like to see addressed prior to publication.

Changes to figures:
• Figure 1D – change y-axis to include the whole bar
Adjusted

• Figure 2C cell 4 trace screenshot includes a text pop-up about the horizontal axis
Fixed, thank you

• Many of the copy number traces throughout the manuscript have been cut off at the bottom and are not showing the x-axis (figure 1C, 2C, S2B, S2E, S3B, S3C, S4B, S5B, S6B)
Response: We now added 0/noise in Fig. 2C for clarification. Added X axis labels in Fig. 1C, Fig. 3D, Fig. S3, Fig. S4, Fig. S5, Fig. S6B

Suggestion for text:
I stated earlier concerns about variation in the location of the chromosome 16 cut-site in relation to the centromere identified in different genome browsers. While I feel this point was not fully addressed, I appreciate the authors re-running their analysis for verification. While I initially found the variation in centromere location surprising, further searching unearthed previous evidence in cell lines for centromeric shifts between the hg38 and T2T alignments
(DOI: https://doi.org/10.26508/lsa.202201807). I think it would be a nice addition to state that this centromeric change is not as unexpected as I believe many readers will think.

Thank you for pointing out this important study, relevant to the results reported here. We are now citing it.

- The statement/claim that the functional centromere can be mapped, seems preposterous. The data does not fully support the claims as other possible reasons for the observations can be deduced. The position of the guide is based on the reference genome. The pericentromeric regions are very variable amongst individuals. How sure are the authors that the pericentromeric region in the embryo genomes are similar to the reference genome? Structural variation in the region would change the outcome and lead to a different interpretation.

Response: this section was extensively revised and is now a minor point. We now have access to a T2T genome assembly, and the gRNA which maps to the p arm on hg19 and hg38, maps to the q arm on T2T. Our data suggested that the gRNA targeted the q arm, as the q arm was primarily affected by segmental aneuploidies. We write: "One gRNA site was located on the p arm of chromosome 16 according to the hg38 genome assembly (Chr16:35126545 at 16p11.1), and to the q arm according to the T2T genome assembly 16q11.2 at chr16:39002348. The q arm was primarily affected by Cas9 cleavage at that site, congruent with the location relative to the centromere according to T2T (Fig. S4). At the time of gRNA design, the T2T assembly was not yet available – frequent q arm changes pointed to an error in the annotation of hg38, and are validating T2T."

I have some concerns about this change. Conducting a BLAST search of the gRNA sequence provided in Table S1 to the T2T-CHM13v2.0 genome using the NCBI Genome Data Viewer, the browser places the sequence mostly within the centromeric region but is still located on the p-arm. However, when using the UCSC Genome Browser, the browser places the sequence in 16q11.2. My look into this was very quick (and likely simple to explain), but I would appreciate a slightly deeper description of this change.

If we assume that the Cas9 cut-site <u>has</u> switched from the p-arm to the q-arm I am confused by some conclusions made with the SNP maps. When looking at chromosome maps in Fig. S4 (annotation from hg19 and centromere placement from T2T), it is stated that there has been a "loss of genomic material between the Cas9 cut site and the centromere…" (lines 196-197). However, with the hg19 and T2T combination this conclusion is based on a cluster of deleted SNPs that would (if located on the q-arm) theoretically fall within the highly repetitive proximal region of chromosome 16q which has been a clear gap in all historical assemblies. I am questioning whether combining the alignments like this is doable or if it is leading to skewed conclusions. I understand that it would be a particularly large undertaking, but for any conclusions based on the Chr 16p arm gRNA the entire SNP map should be converted to the T2T alignment.*

Genome Data Viewer

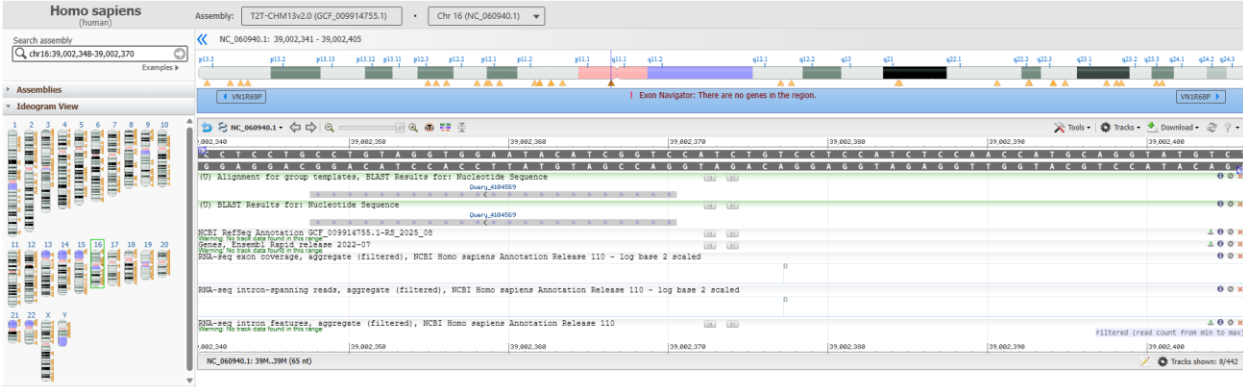

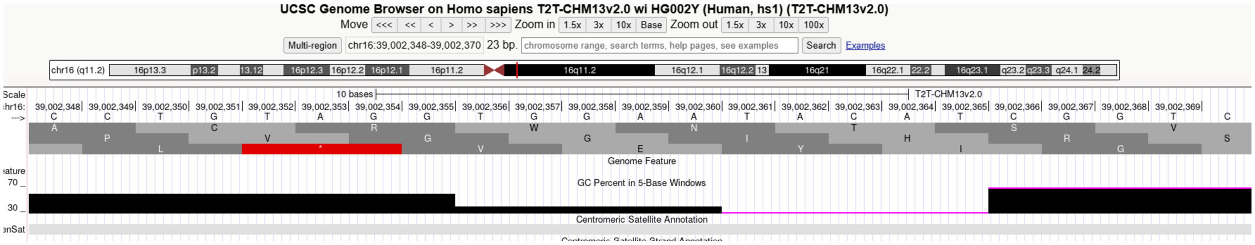

Asymmetric Chromosomal Consequences at Double Strand Breaks in Human Embryonic Development

Turocy et al., 2026

DNA repair mechanisms in the human embryo remain poorly understood and DSBs can have adverse consequences, including chromosome loss. In this revised study, the authors test the effects of CRISPR/Cas9 mediated genome editing on genome stability in human embryos. The study show concerning results where 1/3 of the CRISPR/Cas9 targeted chromosomes in human embryos show either whole, segmented, or fragmented chromosome loss. Moreover, the chromosomal alterations were asymmetric relative to an induced DSB: acentric arms showed complementary gains and losses, and overall were mostly lost, while centric arms were biased toward losses.
Using a variety of methods, the study shows that broken chromosomal fragments that contain centromeres are lagging during mitosis, and that their segregation is often abnormal and can result in exclusion of the chromosome fragment from the cell, secondary breakage and attrition of genomic material between the cut site and the centromere. In contrast, acentric chromosomal fragments do not show an additional break or attrition at the CRISPR/Cas9 cut site. These results highlight the risks of chromosomal-scale changes in CRISPR/Cas9 genome editing and reveal how a single DSB destabilizes am entire chromosome in the human embryo. Lastly, the author propose that these asymmetric differences are arising because the original DSB was not repaired. Given the potential of CRISPR/Cas9 mediated genome editing in treating inheritable genetic disorders it is important to evaluate its genomic effects in human embryos. This study reveals that clinical use of CRISPR/cas9 genome editing in the embryo is too genotoxic. They also show that DSBs occur spontaneously in the human embryos and that the consequence of induced DSBs are similar to spontaneous DSBs.

I appreciate the authors taking the time to significantly improve and substantiate their data using different approaches, including live-cell imaging that I proposed. I also think that they have answered most of my comments and have improved the work significantly in the revised version. While the results of this study are important for the broad audience, and the experiments are mostly sound, I'm still left with several major issues that should be addressed.

The main issue in my view is novelty. I'm trying to understand what is the main take-home message and is it new? For example, line 335 says: "thus, sister chromatids remain in proximity, while DSB ends are prone to separate from each other". I'm not sure what is novel in these results. It is widely known that an acentric fragment will get lost, and if a fragment contains a centromere, the centromere will mediate segregation and separation. Can the author expand on the novelty of their results across the manuscript compared to what is known in the field as consequences of DSBs in somatic cells and in embryos. For example, do DSBs tend to be repaired more efficiently in somatic cells? Can they compare between the two and demonstrate inefficient repair in embryos?

In addition, while improved, figure legends and text need further review and refinement.

Major points:

1. Novelty –What is the main take-home message and is it new? Can the author expand on the novelty of their results across the manuscript compared to what is known in the field as consequences of DSBs in somatic cells and in embryos.

2. This work, as is, shows the consequences of DSBs in embryos, and the lack of their repair in many instances. However, there is no attempt to explain that mechanistically. What causes embryos to lack in their repair capacity.
3. Fig. 2D – Can a PCR amplify a pericentromeric repetitive region? A positive control from a wt cell is missing. Also, what is "other"? no explanation is given within the fig legend.
4. Figure legends across all figures should be improved to include all necessary details. For example, the legend of Fig. 2D is not sufficiently detailed to understand the data shown. The meaning of "other" is not explained. Fig legends should be detailed and include number of repetitions, and explanations of samples names, etc.
5. Line 209: can the attrition be attributed to technical difficulties in sequencing/SNP analysis of that region? Can some SNPs be harder to amplify? If yes, this should be included in the explanation of study limitations.
6. Line 249: "sister chromatids appeared as separate visual signals in mitosis … suggesting they were not a covalently linked entity". – why would the authors expect sister chromatids to be covalently linked? Sister chromatids are held together at the centromere by cohesin. The broken arms do not have a centromere and are not supposed to be held together. I'm not sure I understand the novelty of these results.
7. Additional schematics panels will improve the understanding of current Fig. 4E and 4G.
8. Fig. 5B is not annotated. What does each microscope image represent?? What is control, what is Cas9 treated, colors of probes etc. Basic Figure preparation and revision is absent.
9. The authors propose that if the DSB is not repaired, than it will likely progress into linkage of the centric fragments resulting in a lagging chromosome with two centromeres that is different than a dicentric chromosome that arise through break-fusion-bridge mechanism. Indeed, the break-fusion-bridge mechanism should lead to duplications, which are not observed in the results presented here, reducing the chances that what we are seeing are indeed dicentric chromosomes. Perhaps long-read sequencing could help resolve the structure of the derivative chromosome observed here.
10. The author should clarify across the text what is the difference between fusion of the centric fragments that lead to dicentric chromosome compared to linkage of the sister chromatids that also results in a dicentric structure as their model propose. This is central to their work and their model but is not easy to understand and discriminate between the two.

Minor points:

1. Line 118 "… due to a chromosomal error on the other chromosome". I suggest rephrasing 'chromosomal error' to arm loss or segmental loss.
2. Line 153: "breakpoints .. are located at WWOX, an olfactory receptor gene cluster, WDR25, DPP10, RPTOR…". Are these known as fragile sites? if yes, it would be worth to mention it in the text.
3. Fig. S4 – no individual panels (A, B, C…) make it harder to follow the text. Also, addition of chromosomal schematics can be helpful in interpreting the sequencing data.
4. Fig. S5 - addition of chromosomal schematics can be helpful in interpreting the sequencing data.
5. Legend of Fig. 4D is not complete and is unclear. Many details are missing.
6. Lines 261 – 263:" not all expected genomic material…" sentence is not clear. Please rephrase.
7. Paragraph related to Fig. 4F-M (p. 13-14) is unclear. Please rephrase.
8. Line 289: "two chromosomes were again apparent" – please change to DNA entities or fragments as one cannot know from microscopy if these are full chromosomes or fragments.

9. Line 292: “… can get expelled from dividing cells, resulting in bias of spontaneous … towards chromosome loss” – micronuclei do not have to be expelled. The DNA they contain can also be fragmented and lost.
10. It is not clear to me what can be learned from Fig. S6D. It is completely blurry.
11. Fig. 5D missing Y axis
12. Line 409: “the nature of this linkage may be end-joining of sister chromatids resulting in bicentric chromosomes”. The correct term is ‘dicentric’. In addition, lagging chromosomes can be a result of erroneous microtubule attachment (merotelic attachment). They do not necessarily represent dicentric chromosomes.
